# Understanding How Cationic Polymers’ Properties Inform Toxic or Immunogenic Responses via Parametric Analysis

**DOI:** 10.1021/acs.macromol.3c01223

**Published:** 2023-09-08

**Authors:** Adam M. Weiss, Marcos A. Lopez, Benjamin W. Rawe, Saikat Manna, Qing Chen, Elizabeth J. Mulder, Stuart J. Rowan, Aaron P. Esser-Kahn

**Affiliations:** † Pritzker School of Molecular Engineering, University of Chicago, 5640 S Ellis Ave., Chicago, Illinois 60637, United States; ‡ Department of Chemistry, University of Chicago, 5735 S Ellis Ave., Chicago, Illinois 60637, United States

## Abstract

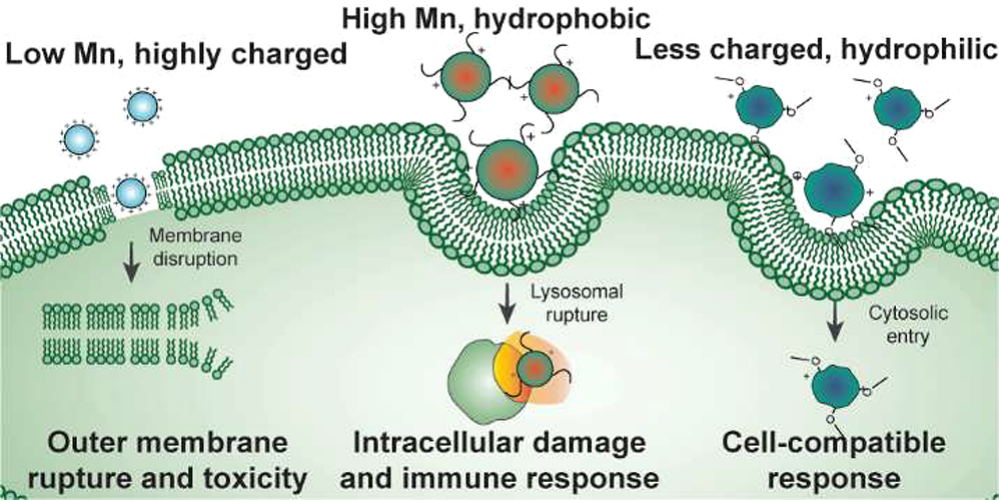

Cationic polymers are widely used materials in diverse biotechnologies. Subtle variations in these polymers’ properties can change them from exceptional delivery agents to toxic inflammatory hazards. Conventional screening strategies optimize for function in a specific application rather than observing how underlying polymer–cell interactions emerge from polymers’ properties. An alternative approach is to map basic underlying responses, such as immunogenicity or toxicity, as a function of basic physicochemical parameters to inform the design of materials for a breadth of applications. To demonstrate the potential of this approach, we synthesized 107 polymers varied in charge, hydrophobicity, and molecular weight. We then screened this library for cytotoxic behavior and immunogenic responses to map how these physicochemical properties inform polymer–cell interactions. We identify three compositional regions of interest and use confocal microscopy to uncover the mechanisms behind the observed responses. Finally, immunogenic activity is confirmed *in vivo*. Highly cationic polymers disrupted the cellular plasma membrane to induce a toxic phenotype, while high molecular weight, hydrophobic polymers were uptaken by active transport to induce NLRP3 inflammasome activation, an immunogenic phenotype. Tertiary amine- and triethylene glycol-containing polymers did not invoke immunogenic or toxic responses. The framework described herein allows for the systematic characterization of new cationic materials with different physicochemical properties for applications ranging from drug and gene delivery to antimicrobial coatings and tissue scaffolds.

## Introduction

Cationic materials have found extensive use in immunology and biomedicine on account of their physicochemical properties. They can traverse and disrupt cell and organelle membranes and are therefore used as transfection reagents and antimicrobial coatings^1−3^ They are also widely used in drug and gene delivery as they interact with negatively charged biomacromolecules.^3−5^ Clinically, cationic materials ranging from poly(ethyleneimine) to Lipofectamine-2000 to chitosan remain key components of gene editing technologies,^6,7^ drug delivery systems,^8,9^ and most recently as ionizable lipid–polymer hybrids that comprise lipid nanoparticles (LNPs).^10,11^

Despite excellent work optimizing these materials for each application, there is an inherent compromise among functionality, immunogenicity, and toxicity. Immunogenicity and toxicity can be unintended consequences of a desired cellular interaction such as endosomal disruption. These unintended consequences can limit the clinical translation of a polymer-based system. In this work, we explore what features of cationic polymers lead to immunogenic or toxic response. Polymers that disrupt cellular membranes for endosomolysis or transfection can cause cell membrane rupture, neutrophil recruitment, and necrosis in the tissue niche.^12−14^ Likewise, polymers that complex with negatively charged biomacromolecules for drug delivery can activate pattern recognition receptors or the complement system when unbound from their cargo.^15^ Specifically, cationic polymers activate the NOD-, LRR-, and pyrin-domain-containing protein 3 (NLRP3) inflammasome via lysosomal rupture, resulting in IL-1β secretion and pyroptosis.^16−22^ IL-1β can initiate productive or damaging immune responses, depending on delivery context.^23−28^ Notably, cationic polymers and lipids in mRNA vaccines induce high levels of IL-1β secretion.^23^ Currently, costly *in vivo* models of (pre)clinical biocompatibility are required before materials can be translated in the clinic, slowing research and leading to costly failures. We hypothesized that mapping the immunogenic and toxic chemical space of cationic polymers would allow researchers to be better informed while designing materials so they can avoid or generate a desired response.

To date, research on the interactions between cationic polymers and the innate immune system has been sporadic and application-driven, leading to conflicting conclusions about the ideal material properties for an application.^9^ In gene delivery, positive charge, hydrophobicity, molecular weight, and formation of self-assembled nanostructures can modulate toxicity.^29−33^ In innate immunology, the ratio of charged to hydrophobic groups in a copolymer can modulate lysosomal rupture, inflammasome activation, and IL-1β secretion.^18^ Charge, hydrophobicity, and p*K*_a_ can further inform lysosomal rupture and inflammasome activation.^17−20^ Finally, physicochemical properties can modulate hemolytic and antifouling capacities of antimicrobial polymer coatings.^1,13,34−39^ Paslay et al. assayed the antimicrobial properties of water-soluble poly(methacrylamides) and found that primary amines were superior to tertiary amines in neutralizing *E. coli* growth.^34^ While each result provides structure–function information related to one specific application, they do not connect the polymers’ properties to their interactions with cells at a mechanistic level.

**Figure 1:**
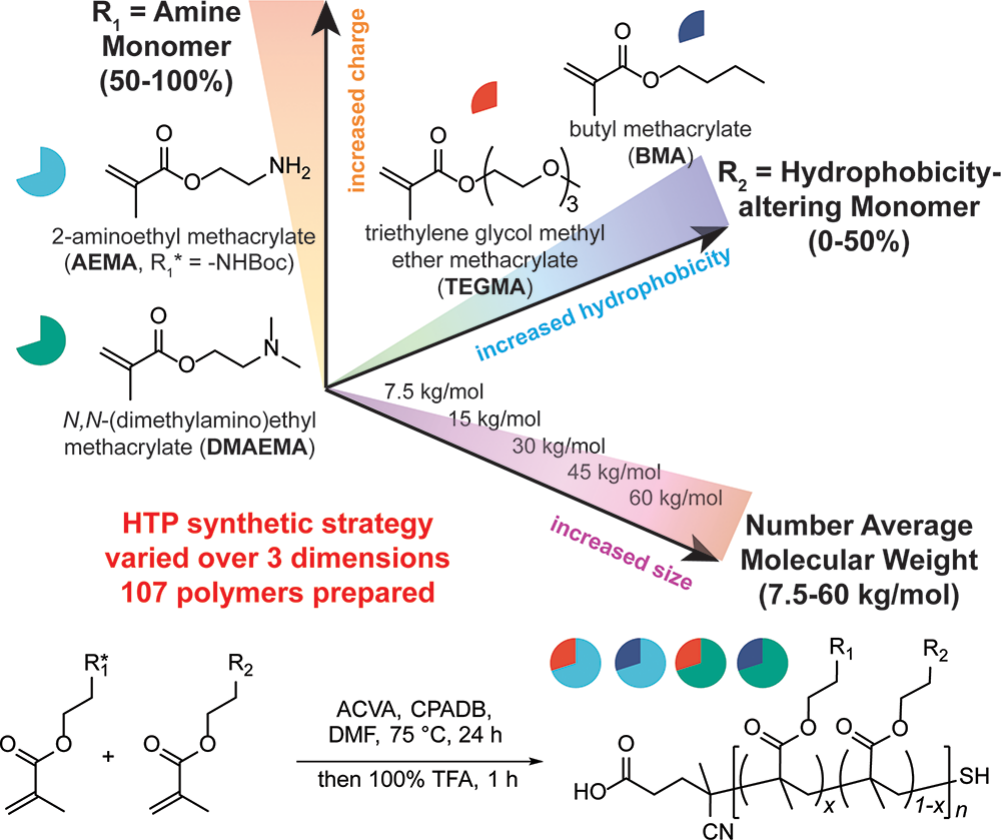
High throughput synthesis of 107 polymers. Statistical copolymers were prepared via RAFT polymerization using 50–100% of the amine-containing monomers (R_1_ = BocAEMA or DMAEMA) and 0–50% of the hydrophobicity-modifying monomers (R_2_ = TEGMA or BMA) at five different molecular weights (*M*_n_ = 7.5–60 kg/mol) to map a broad domain space of physicochemical properties.

In this work, our goal was to map the cationic polymer properties and cellular responses using high-throughput screening. Recent advances allow rapid, high-throughput synthesis of materials with controlled composition and molecular weight using living polymerization.^40,41^ We sought to probe how polymers with different charged groups, hydrophobicity-altering groups, and molecular weights induce immunogenicity via the NLRP3 inflammasome or toxicity via necrotic cell death. Using RAFT polymerization, we prepared 107 methacrylate-based statistical copolymers that varied charge, amine identity, and hydrophobicity in different proportions and ratios within the copolymers. We used two amine-containing monomers: *N*,*N*-(dimethylamino)ethyl methacrylate (DMAEMA) and 2-aminoethyl methacrylate (AEMA). These monomers varied in the net charge at biological pH and displayed different hydrogen-bonding capacities, allowing us to test if it was charge or amine identity that contributed to an observed response. To probe the effects of hydrophobicity, we included two additional monomers: triethylene glycol methyl ether methacrylate (TEGMA) and butyl methacrylate (BMA). A series of copolymers were synthesized that contain either of the amine monomers with 0–50 mol % of TEGMA or BMA statistically incorporated into the backbone to create a systematic sweep of this domain space. For each combination, polymers were prepared at five molecular weights (7.5, 15, 30, 45, and 60 kg/mol). The resulting polymer library is composed of water-soluble polymers that vary in charge (50–100 mol % amine monomer), hydrophobicity (0–50 mol % hydrophobicity-altering monomer), and size (7.5–60 kg/mol) (Figure 1). This parametric and minimalist design allowed us to probe how polymers’ properties impact the resultant immunogenic or immunotoxic responses of immune cells.

## Experimental Section

### Mouse, Cell, and Chemical Sourcing

All chemicals and cell culture reagents were obtained from Sigma-Aldrich or Thermo Fisher and used without further purification unless otherwise noted. Nigericin and phorbol 12-myristate 13-acetate (PMA) were obtained from Cayman Chemical Company. LysoView 633 was obtained from Biotium. Cytochalasin D was obtained from R&D Systems. THP-1, THP-1 NLRP3-KO, and THP-1 ASC-GFP cells were obtained from InvivoGen. A549 and HeLa cells were obtained from ATCC. HEK FIRE-pHLy cells^42^ were obtained as a generous gift from Aimee Kao (Stanford University). PBMCs were obtained from the Precision Medicine Group. All cells were maintained at 37 °C and 5% CO_2_ in RPMI-1640 or DMEM with 10% (v/v) heat inactivated fetal bovine serum (HI-FBS) and selection antibiotics according to the vendors’ specifications. Antibodies used for flow cytometry are listed in the Supporting Information. The 6-week-old C57Bl/6J or B6.129S6-Nlrp3^tm1Bhk^/J mice were purchased from Jackson Laboratory, housed under controlled conditions, and allowed to acclimatize for ≥1 week prior to use. MoDCs and BMDCs were isolated as described in the Supporting Information. All animal studies were conducted with the approval of the University of Chicago Institutional Animal Care and Use Committee, and animals were maintained in accordance with the National Institutes of Health guidelines. All data unless otherwise noted are analyzed and plotted in GraphPad Prism 9 or Igor Pro 7. One- or two-way ANOVA with residual multiple comparison testing was used for all statistical analyses shown.

### Synthesis of Polymer Library

The polymer library was synthesized on a 100 mg scale using an Unchained Junior process chemistry robot. Triethylene glycol methyl ether methacrylate (TEGMA) and butyl methacrylate (BMA) were passed through an alumina column prior to use. *N*,*N*′-(Dimethylamino)ethyl methacrylate (DMAEMA) was distilled under reduced pressure prior to use. 4-Cyanopentanoic acid dithiobenzoate (CPADB) and 4,4′-azobis(4-cyanovaleric acid) (ACVA) were recrystallized from methanol prior to use. Boc-(2-aminoethyl) methacrylate (BocAEMA) was synthesized as reported previously^43^ (see the Supporting Information) and recrystallized from 1:1 hexanes:DCM prior to use. Under a nitrogen atmosphere, purified BocAEMA, DMAEMA, BMA, and TEGMA were dissolved at 250 mg/mL in dry DMF, and CPADB and ACVA were dissolved at 10 mg/mL in DMF. All reagents were added in appropriate ratios to 2 mL vessels and diluted to a final volume of 1 mL in DMF. The vessels were then heated to 72 °C and shaken at 1500 rpm. After 24 h, the vessels were cooled and exposed to air to quench the reaction. From each sample, a 25 μL aliquot was collected for size exclusion chromatography. The polymers were treated with 500 μL of TFA, precipitated into 50 mL of 1:1 diethyl ether:hexanes, and collected by centrifugation. All polymers were then treated with 1 mL of TFA for 1 h to remove Boc protecting groups or as a control. After 1 h, the polymers were reprecipitated into 50 mL of 1:1 diethyl ether:hexanes, collected by centrifugation, and taken up in 5 mL dH_2_O. The polymers were dialyzed sequentially for 24 h each against 0.5 M NaCl and dH_2_O and freeze-dried to obtain the polymers as white or pink aerogels. Additional copolymers for analysis of monomer consumption or AF488 labeling were synthesized by hand using an analogous method; here, air-free conditions were achieved by bubbling argon through the reaction for 30 min prior to heating, and the reaction was stirred with a stir bar.

### Characterization of Polymer Library

Size exclusion chromatography (SEC) was conducted in DMF with 0.01 M LiBr additive at 50 °C using a Tosoh EcoSEC system equipped in series with Tosoh SuperAW3000 and Tosoh SuperAW4000 columns. 25 μL aliquots of the crude reaction mixture were diluted in 1 mL of DMF + 0.01 M LiBr, and 15 μL was injected for each chromatograph. The polymer molecular weight was calculated relative to PMMA standards by using the Tosoh EcoSEC analysis software. ^1^H NMR was conducted at 400 MHz on a Bruker DRX instrument equipped with a BBO probe using Topspin 1.3 and analyzed using MestreNova software (64 scans/polymer). All NMR spectra were referenced to the residual D_2_O solvent peak (4.79 ppm), and the relative integration ratio of diagnostic peaks was used to calculate the mol % of comonomers incorporated into the polymer scaffold (see the Supporting Information). DOSY-NMR molecular weight validation and kinetic analyses were conducted at 500 MHz on a Bruker Avance-II+ spectrometer equipped with a QNP probe using Topspin 2.1 as detailed in the Supporting Information. Aggregation analysis strategies including DLS, TEM, and UV–vis spectroscopy as well as methods for the determination of p*K*_a_ are described in the Supporting Information.

### *In Vitro* IL-1β and LDH Screening

Prior to analysis, polymers were dissolved at 1000 μg/mL in sterile PBS and serially diluted to achieve 10× stock solutions at the indicated concentrations. THP-1 cells were plated at 1.8 cells/well in a 96-well plate and primed with 100 EU/mL ultrapure LPS-EB (InvivoGen). After 3 h, cells were pelleted, washed with PBS, resuspended in THP-1 media, and treated in triplicate with a final concentration of 100, 50, 25, 12.5, or 6.25 μg/mL polymers (or 10 μM nigericin and PBS as positive and negative controls, respectively). After 5 h, the supernatant was collected and subjected to a human IL-1β ELISA Kit (Thermo Scientific) and CyQUANT LDH cytotoxicity assay (Thermo Fisher) according to the manufacturer’s procedures. Similar protocols were conducted with BMDCs and MoDCs as described in the Supporting Information. Principal component analysis was conducted using MATLAB 2020B, and data were plotted using RStudio with the scatterpie package (https://github.com/GuangchuangYu/scatterpie).

### Time Lapse Confocal Microscopy

For confocal imaging, 5 cells were plated in a four chambered, 35 mm glass bottom dish (Grenier Bio-One) and allowed to adhere for 24–48 h. THP-1, THP-1 ASC-GFP, and THP-1 NLRP3-KO cells were differentiated for 24–48 h with 25 nM PMA to induce an adherent macrophage-like phenotype. Cells were then washed and primed with 100 EU/mL ultrapure LPS-EB (InvivoGen) in medium for 3 h. In the last 30 min of priming, lysosomal dyes (2 μM LysoSensor Green DND-189 or 1:1000 dilution of LysoView 633) and 2.5 μg/mL cytochalasin D were added. Cells were then washed, treated with phenol red-free THP-1 or HEK medium containing 10 μg/mL Hoechst 33342 or 10 μg/mL propidium iodide, and placed into focus using a 3i Marianas confocal microscope (40× oil lens) with an OKO full environmental control chamber. The imaging chamber was held at 37 °C and supplemented with CO_2_ for the duration of imaging. After cells were focused within the imaging plane, polymers were added (100 μg/mL working concentration), and time-lapse images were collected in 2 min intervals for 60 min. Data were processed using 3i SlideBook 6 software. For studies with THP-1 ASC-GFP cells, specks and propidium iodide-stained cells were counted by a single blind volunteer.

### Polymer Labeling and Imaging

Polymers containing a 70:30 amine:TEGMA/BMA monomer ratio with molecular weights of 15 and 60 kg/mol were used for labeling studies. AEMA-containing polymers were directly labeled, while DMAEMA-containing polymers containing 5 mol % AEMA were synthesized as described above for functionalization with AF488 NHS Ester. For labeling, 20 mg of polymer was dissolved in 5 mL of ultrapure water, the vial was wrapped in foil, and 20 μL of 5 mg/mL AF488 NHS Ester (in DMSO) was added with stirring. The reaction was allowed to proceed overnight; unreacted AF488 was removed by dialysis against 4 L of water for 3 days in the dark (changing dialysate twice daily), and polymers were lyophilized to obtain yellow aerogels. THP-1 cells were then plated in a four chambered dish, differentiated with PMA, primed with ultrapure LPS-EB, and stained with LysoView 633 as described above. Cells were washed and incubated with 100 ug/mL final concentration of the AF488-tagged polymers, 10 μg/mL Hoechst 33342, and 10 μg/mL propidium iodide in phenol red-free THP-1 medium for 30 min. Cells were washed again, plated in 500 μL of phenol red-free medium, and imaged using a Marianas 3i confocal microscope (40× oil lens).

### Lysosomal pH Analysis Using FIRE-pHLy Construct

HEK FIRE-pHLy cells were plated at 5 cells/well in a 96-well plate, rested for 2 h, and then treated with 25 μg/mL of the indicated polymers for 1 h. Cells were then detached with EDTA, washed, resuspended in PBS + 2% FBS + 5 mM EDTA, and analyzed with a LSR Fortessa (BD Biosciences) flow cytometer. The median fluorescence intensity ratio of mTFP1 (measured on a BV510 detector) to mCherry (measured on a PE-CF594 detector) was used to determine the lysosomal pH.

### Biocompatibility Study with Polymers

The 8-week-old female C57Bl/6J mice were injected with 50 μL of 1 mg/mL of the indicated polymers in PBS and monitored for adverse effects for 30 min. Body weight was monitored at 1, 3, and 6 h after injection by using a digital, no-touch infrared thermometer (Home Depot). At 6 h, mice were bled via the submandibular vein and then sacrificed for spleen harvest. Systemic cytokines in the serum were assayed via LEGENDplex Mouse Inflammation 13-plex (BioLegend). Splenocytes were prepared, counted via a hemocytometer, and then plated at 2.5 × 10^6^ cells/well in a 96-well plate. Cells were stained for viability and cell surface markers, fixed using Cytofix/Cytoperm (BD Biosciences), stained for intracellular cytokines, and analyzed with a Novocyte Penteon (Agilent) flow cytometer. Antibodies used for staining are provided in the Supporting Information. Flow cytometry data were analyzed using FlowJo v10.8.1.

## Results and Discussion

### Cationic Polymer Library Synthesis via RAFT Polymerization

To evaluate the ability of cationic polymers with a breadth of physicochemical properties to invoke immunogenic or toxic responses, a library of 107 water-soluble polymers containing two amine monomers (*R*_1_ = 50–100 mol % DMAEMA or AEMA) and two hydrophobicity modifying monomers (*R*_2_ = 0–50 mol % BMA or TEGMA) at different monomer ratios and number-average molecular weights (*M*_n_ = 7.5–60 kg/mol) were synthesized. RAFT polymerization was employed with 4,4′-azobis(4-cyanovaleric acid) (ACVA) as initiator and 4-cyanopentanoic acid dithiobenzoate (CPADB) as the chain transfer agent (Figure 1). A Boc-protected form of AEMA (BocAEMA) was used as the monomer for AEMA-containing polymers to prevent side reactions with the primary amine (Figure S1).^43^ All polymers were synthesized on a 100 mg scale by using an automated process chemistry robot. In contrast to previous studies of self-assembled polymers,^19^ statistical copolymers lacking secondary structure were targeted to rule out effects of size, shape, or orientation of assemblies on the immune response.^9,44,45^ Analysis of the polymerizations of DMAEMA or BocAEMA with TEGMA or BMA in DMF confirmed similar rates of incorporation of amine monomers with hydrophobicity-modifying monomers into the polymer scaffold, and the target molecular weights were achieved after 18 h (Figure S2). In a typical synthesis, 50–70 mg of polymer was obtained after purification (50–70% yield) for further study.

To characterize the polymer library, size exclusion chromatography (SEC) and proton nuclear magnetic resonance (^1^H NMR) spectroscopy were used to evaluate the molecular weight and monomer composition, respectively (full characterization results are tabulated in Figure S3). While extensive characterization is impractical for a library of this size, our characterization efforts are consistent with previous reports of high throughput polymer screens for biological applications.^46,47^ The crude, Boc-protected form of AEMA-containing polymers was used for SEC analysis, as they were soluble in DMF. After synthesis, polymers were precipitated, deprotected by treatment with trifluoroacetic acid (TFA) for 1 h, precipitated again, and dialyzed for purification. DMAEMA-containing polymers were similarly treated with TFA, although they did not require Boc deprotection, as a control. Polymers were freeze-dried and analyzed via ^1^H NMR in D_2_O to determine the final molar ratio of monomers in the polymers (i.e., mol % of AEMA or DMAEMA and BMA or TEGMA after purification) (Figure S4). To confirm that TFA treatment did not result in decomposition or cross-linking of the polymers during deprotection, DOSY-NMR was conducted on a subset of the polymer library to determine diffusion constants (*D*) (Figure S5). Log(*D*) obtained via DOSY correlated linearly with *M*_n_, suggesting that polymers did not decompose during TFA treatment.^48^ Most experimentally determined *M*_n_ values and NMR compositional ratios were consistent with expectations, having a composition of monomers within 7% of the target, narrow dispersities (usually *Đ* < 1.4), and molecular weight within 30% of the target *M*_n_ (Figure S3). It must be noted that high *M*_n_, DMAEMA-containing copolymers had higher dispersities of *Đ* = 1.3–1.7. While consistent with previously reported trends^49^ and possibly related to polymer–column interactions, care must be taken in comparing 45–60 kg/mol DMAEMA-containing polymers in later biological assays.

After the polymers were successfully synthesized, additional characterization was conducted on a subset of polymers to elucidate their behavior in aqueous solution. Eight polymers were selected that spanned each of the four monomer combinations in a 70:30 molar ratio (AEMA_70_-BMA_30_, AEMA_70_-TEGMA_30_, DMAEMA_70_-BMA_30_, DMAEMA_30_-TEGMA_70_) and two *M*_n_ values (15 and 60 kg/mol) for analysis. First, to test whether the statistical copolymers display self-assembly behavior or aggregate with proteins, dynamic light scattering (DLS) was conducted in FBS-containing cell culture medium at 25 and 37 °C (Figure S6). While all polymer-containing solutions were found to have hydrodynamic radii (*R*_H_) < 20 nm at 25 °C, consistent with reports of single polymer chains and/or proteins in aqueous solution,^50^ those containing BMA were found to form larger structures (*R*_H_ > 50 nm) at biological temperatures of 37 °C. Further evaluation of polymers in cell culture medium via TEM failed to reveal any polymer self-assembly behavior, suggesting that these structures were caused by aggregation with serum proteins such as albumin (Figure S6). These aggregates must be considered in the interpretation of biological data. To confirm the ionization state of AEMA- and DMAEMA-containing copolymers in biological solutions, polymers were titrated with aqueous NaOH (Figure S7). AEMA-containing copolymers had p*K*_a_s ca. 7.1–7.2 while DMAEMA-containing copolymers had p*K*_a_s ca. 6.4–6.7. These p*K*_a_ values stand in agreement with previous reports and are notable as they suggest different net charge under biological conditions, though it should be noted that amine methacrylate copolymers are known to have higher p*K*_a_s in high salt buffers (ca. 8–10) resulting from electrostatic repulsion between charged amine groups.^51,52^ Finally, to confirm that polymers would not alter pH beyond a tolerable level when dissolved in cell culture medium, 100 μg/mL of the polymers (or controls) was added to phenol-red-containing culture medium, and the pH was monitored by absorbance spectroscopy (Figure S8). Again, no significant changes to pH were observed when polymers were added to the culture medium, supporting their use in biological settings. Having successfully synthesized and characterized 107 polymers, we then moved on to screen immune activity and cell death *in vitro*.

### Polymers’ Compositions Inform Immunogenicity and Toxicity

Synthetic polymers have the potential to interact with a large range of biomolecules and organelles to induce an immune response.^9,53^ This response might be characterized by protein adsorption, immune cell activation, or tissue necrosis dependent on the physicochemical properties of the polymer and the route of administration. Optimizing such responses for specific applications is important for the safe implementation of biomaterials. Gene editing tools, mRNA vaccines, and antibacterial coatings all employ cationic polymers.^3^ Many more polymer-based technologies that complex nucleic acids or other biomolecules are in development. Preventing unwanted toxicity and immunogenicity induced by the polymers while maximizing intended biological responses could improve patient outcomes and lower the cost of development. Most *in vitro* screens focus on maximizing biological responses, leading to the possibility for expensive, late-stage biocompatibility failures. As such, our initial studies sought to broadly map the ability of the synthesized polymers to induce an immunogenic or toxic response. With our library of 107 polymers that vary parametrically over several dimensions, we can correlate toxicity and immunogenicity with polymer properties, namely, charge, hydrophobicity, and molecular weight. Such a structure–property map can allow early and rapid identification of monomer compositions that generate (or avoid) desired biocompatibility features for a breadth of biological applications.

Our goal was to determine which characteristics of the polymer correlated to which types of responses. Activation of the NLRP3 inflammasome and subsequent IL-1β secretion are regulators of pro-inflammatory pathways, and many polymers have been shown to activate this immune sensor via disruption of homeostasis.^16−21,26^ Alternatively, many polymers induce toxic responses independently of inflammatory pathways (e.g., via direct membrane disruption).^13,34,54^ Cells were treated with each of the 107 polymers, and secretions of IL-1β and an intracellular enzyme, lactate dehydrogenase (LDH), were assayed in the supernatant. In this way, the polymers’ charge, hydrophobicity, and molecular weight could be mapped against their cellular responses, and emergent features could be observed. THP-1 cells (secondary human monocytes^55^) were treated with lipopolysaccharide (LPS) for 3 h to prime inflammasome formation and then with each of the 107 polymers at 6.25, 12.5, 25, 50, or 100 μg/mL for 5 h. Toxicity was evaluated by a colorimetric LDH secretion assay, and IL-1β secretion was evaluated by an enzyme-linked immunosorbent assay (ELISA). It is key to note that while IL-1β secretion was selected as the main readout in this study, this workflow is amenable to analysis of other pro-inflammatory cytokine (such as TNF-α) or multiplexed readouts. Analysis of other pro-inflammatory cytokines was of interest but outside of the time and cost scope of this proof-of-concept study.

**Figure 2:**
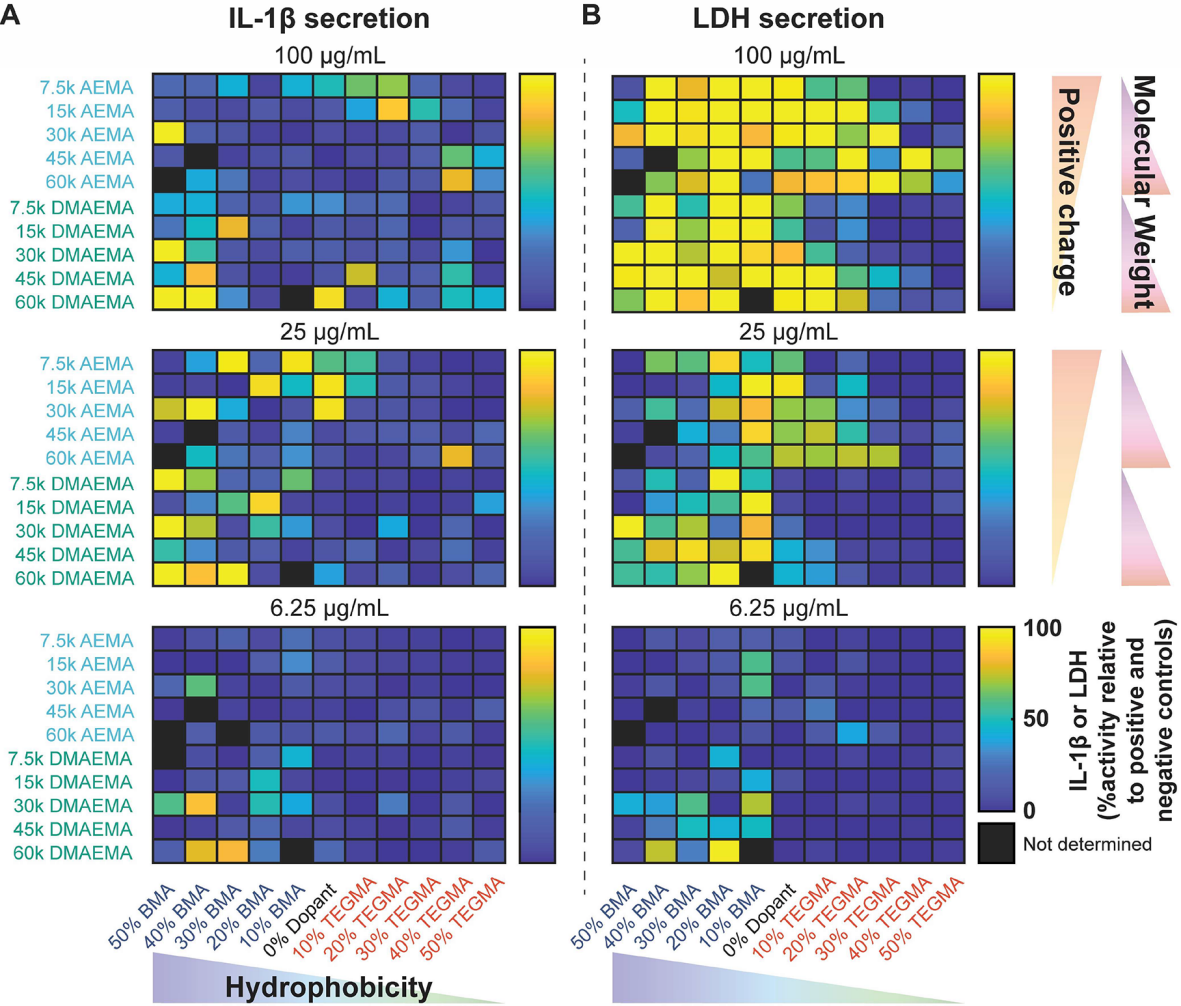
Results and analysis of high throughput immunological and toxicity screening. (A) IL-1β and (B) LDH screens at high (100 μg/mL), medium (25 μg/mL), and low (6.25 μg/mL) concentrations for each of the 107 polymer library entries.

**Figure 3:**
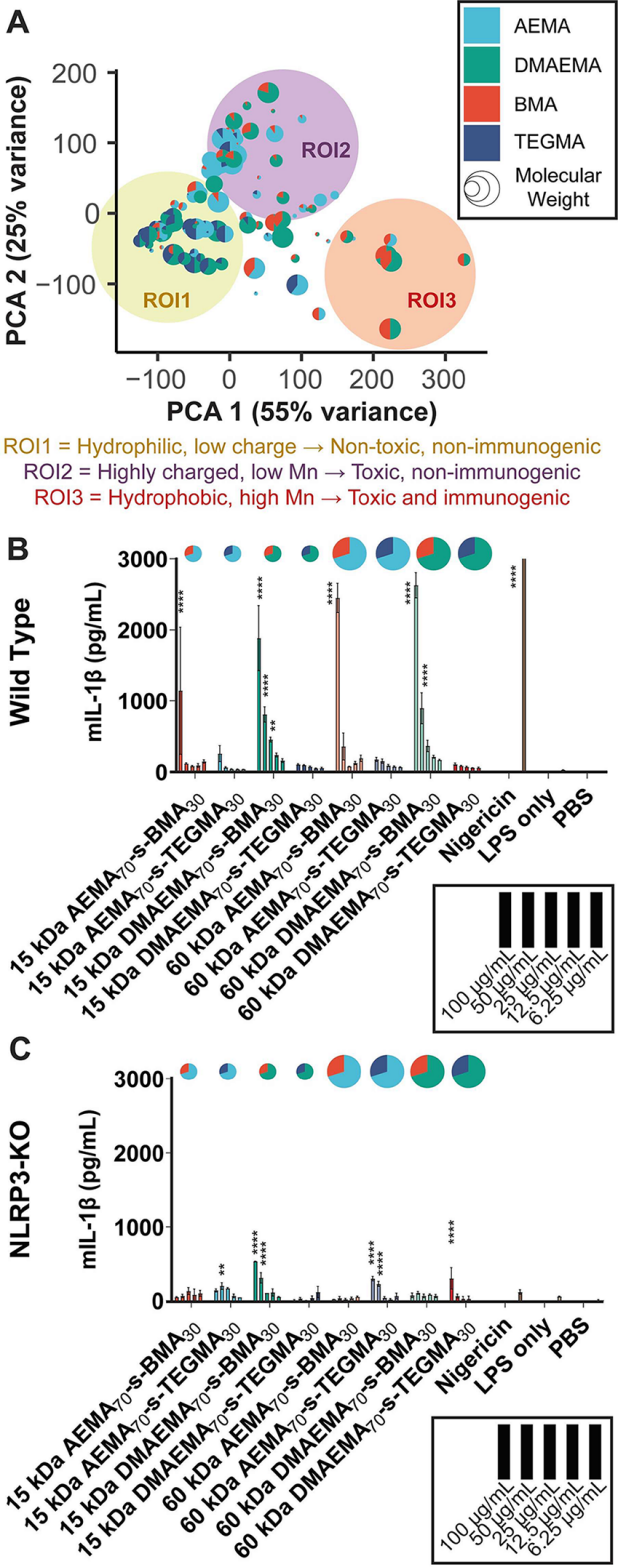
Identification of trends in the high throughput data set. (A) A principal component analysis was conducted on the high throughput screen to identify structure–property relationships. The first two principal component axes (PCA-1 and PCA-2) are plotted. Three regions of interest are identified as shown. (B, C) Validation of IL-1β secretion results in BMDCs from (B) wild-type and (C) NLRP3-deficient mice. Polymers were incubated with LPS-primed BMDCs at the indicated concentrations for 5 h, and IL-1β was analyzed via ELISA. A two-way ANOVA with Dunnett’s multiple comparison’s test was used to determine statistical significance relative to the LPS only treatment (no label = not significant).

The results of high-throughput *in vitro* immunogenicity and toxicity screening at three concentrations are presented in Figure 2, while the full results of this initial screen are provided in Figure S9. We quantified observed trends by assuming that the results at each of the five concentrations tested were independent (5 concentrations × 2 assays = 10 conditions). A principal component analysis was then used to map each polymer property against the observed biological response (Figure 3A). Principal component axes 1 and 2 (PCA-1 and PCA-2) comprised 80% of the variance in the data and revealed three regions of interest. First, the presence of the hydrophobicity-enhancing monomer, BMA, or the charged monomer, AEMA, resulted in toxic polymers regardless of molecular weight or comonomer identity. These results imply that the presence of ≥50 mol % of positively charged, primary-amine-containing monomers or ≥10 mol % hydrophobic monomers in a polymer will likely result in at least partial toxicity. The greatest toxicity was observed when either amine monomer, AEMA or DMAEMA, was copolymerized with 10–20 mol % BMA, demonstrating that positive charge and hydrophobicity interplay to inform toxic responses. These results are consistent with previous reports that highly charged, hydrophobic materials are effective as antimicrobial coatings and anticancer chemotherapies on account of their high toxicity.^34,35,56^ In most cases, toxicity is reduced as molecular weight decreases, particularly in hydrophobic compounds (≥30 mol % BMA). Second, high molecular weight (*M*_n_ = 45 or 60 kg/mol), hydrophobic copolymers composed of DMAEMA and BMA induced IL-1β secretion, particularly when >20 mol % of BMA was incorporated into the polymer scaffold. This result suggests that increasing the ratio of hydrophobic monomer (BMA) to tertiary amine monomer (DMAEMA) in high-*M*_n_ systems can shift the biological response from a toxic phenotype to an immunogenic phenotype. Finally, copolymers containing both the tertiary amine and hydrophobicity-reducing monomers, DMAEMA and TEGMA, only resulted in a toxic response at high molecular weight (*M*_n_ ≥ 45 kg/mol) and with <20 mol % TEGMA. This result demonstrates that in the design of polymers for nontoxic applications like gene delivery, a composition of DMAEMA and TEGMA with ≥20 mol % of TEGMA and/or molecular weights below 45 kg/mol is expected to be biocompatible. Polymers containing DMAEMA and TEGMA have lower p*K*_a_s, meaning that they have a lower degree of positive charge at biological pH, in tandem with higher water solubility. Others have reported that these features reduce protonation of the polymer backbone and enhance transport across (intra)cellular membranes while mitigating membrane rupture,^57,58^ perhaps accounting for these polymers’ biocompatible properties.

These combinations of features, placed in context, present the compelling observation that it is not a single feature that drives toxicity or immunogenicity. Rather, it is the ratio among hydrophobicity, charge, and molecular weight in a polymeric scaffold. While reducing positive charge density and reducing hydrophilicity through the incorporation of chemical groups (such as TEGMA) are known to enhance biocompatibility, our screening methods provide a map of cationic polymer properties that can afford different responses. With these principles and “map” of design parameters in place, a particular polymer might be adjusted to accommodate functional performance. These results are independent of aggregation propensity in cell culture media (Figure S6), highlighting complex polymer–cell interactions which may allow for these phenomena. We envision the broad applicability of this screening strategy to accelerate research in polymer-based drug delivery systems, antimicrobial materials, and tissue scaffolds.

After conducting this broad screen, we sought to determine whether these observed trends held in primary cells. Eight representative polymers representing each of the major categories of charge, hydrophobicity, and molecular weight (based on the preliminary screening data) were employed in primary cell studies. The eight polymers consist of each of the four comonomer combinations at a 70:30 ratio (AEMA_70_-BMA_30_, AEMA_70_-TEGMA_30_, DMAEMA_70_-BMA_30_, and DMAEMA_70_-TEGMA_30_) prepared at 15 and 60 kg/mol. Within this set, 15 kg/mol AEMA_70_-BMA_30_ is representative of polymers with the highest toxicity, 60 kg/mol DMAEMA_70_-BMA_30_ is representative of inflammasome-activating polymers, and 15 kg/mol DMAEMA_70_-TEGMA_30_ is representative of nontoxic polymers. LDH production and IL-1β secretion were assayed in monocyte-derived dendritic cells (MoDCs), isolated from peripheral human blood, and bone marrow derived dendritic cells (BMDCs), isolated from wild-type or NLRP3-deficient C57Bl/6J mice (Figures 3B,C and S10–S11). In MoDCs and wild-type BMDCs, comparable IL-1β and LDH secretion trends were observed relative to those in THP-1 cells, supporting the clinical relevance and cross-species translatability of the primary screen results. Repeating the assay in BMDCs from NLRP3-deficient mice reduced IL-1β secretion while having a minimal effect on toxicity (Figures 3C and S11). It is worthy of note that after treatment of NLRP3-deficient BMDCs with any of the polymers or nigericin, lower but significant levels of IL-1β are still observed in many cases, suggesting either incomplete knock out of NLRP3 gene or IL-1β secretion through alternative cell death pathways.^26^ Finally, we also assayed the other pro-inflammatory cytokines, IL-8 and TNF-α in PBMCs (Figure S11). No differences between groups in IL-8 secretion were observed, although treatment with most of the toxic or immunogenic polymers resulted in TNF-α release, especially at higher concentrations.

We then sought to test whether the map of physicochemical properties and immunotoxic responses generated in this screen would match those of other positively charged, hydrophobic polymers. Some of the most common polymers in this class are branched poly(ethylenimine) (PEI)-based systems used to deliver nucleic acids.^15^ These polymers are water-soluble and contain a dendrimer-like scaffold of secondary and tertiary amines. Based on our screening data, PEI should fall within the ratio of positive charge to hydrophobicity that induces IL-1β production and toxicity. LDH and IL-1β production were assayed when BMDCs were treated with branched PEI with three molecular weights: 60, 10, and 1.8 kg/mol. It was found that branched PEI behaved similarly to copolymers composed of DMAEMA and >20 mol % BMA. 60 kg/mol branched PEI-induced IL-1β secretion at all concentrations tested, while 10 and 1.8 kg/mol PEI induced toxicity in the absence of inflammasome activation except at the highest concentrations tested (Figure S12). Typical gene delivery protocols employ 10–20 μg/mL of PEI, so the lower concentrations tested hold insight into the immune responses generated by clinical gene delivery systems. These results suggest that polymer–cell interactions can induce cell death independent of NLRP3 inflammasome activation but that NLRP3 is required for IL-1β secretion. Given these results, mechanistic studies were undertaken to address why different classes of polymer-induced different responses. While multiple mechanisms are likely involved, a greater understanding of these responses would inspire design criteria for new materials in a breadth of applications.

### Cells Treated with Polymers Undergo Morphological Changes

**Figure 4:**
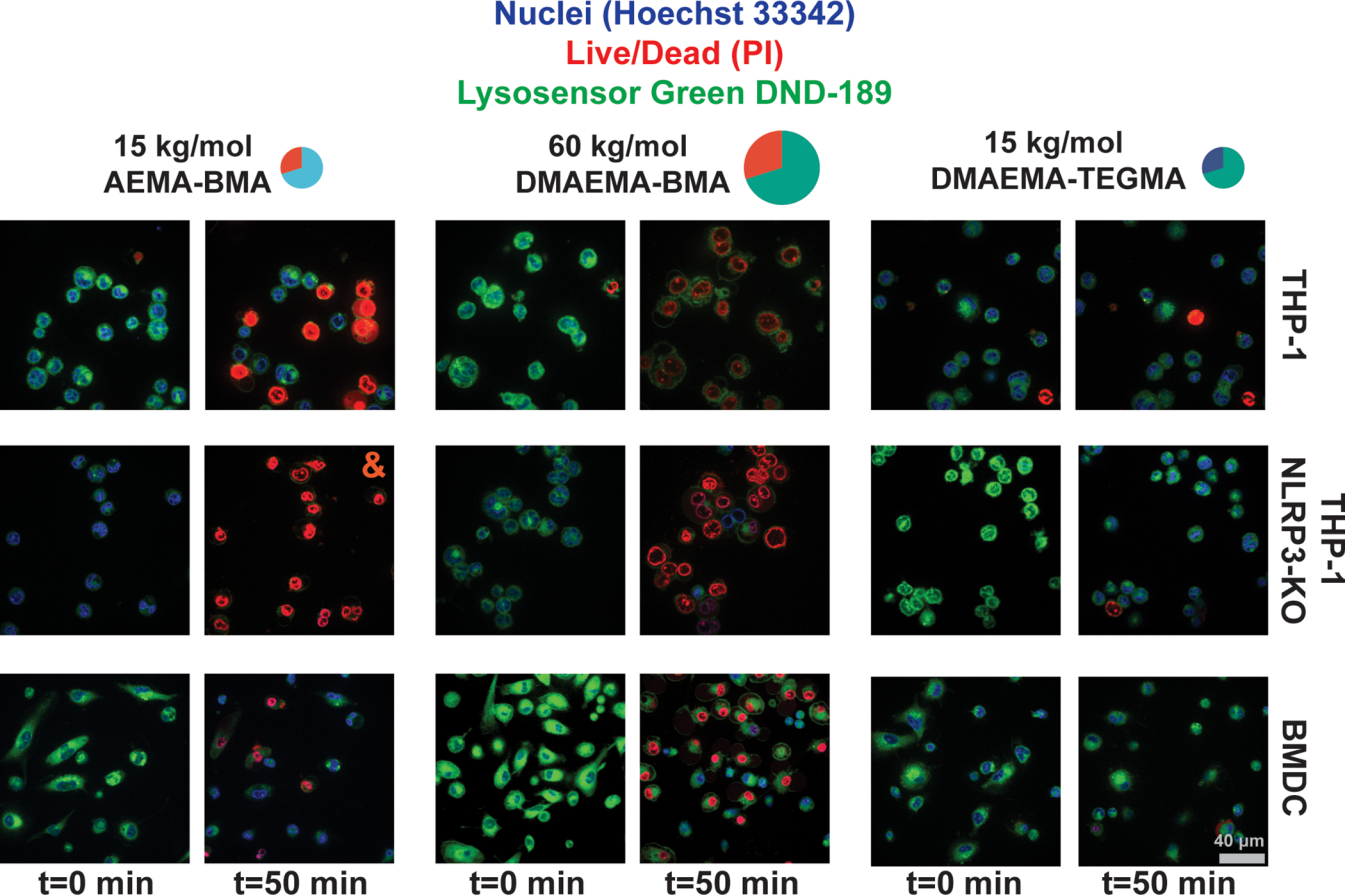
Imaging the rupture of cells treated with representative polymers reveals distinct modes of rupture and death. Cellular swelling was induced by the inflammasome-activating 60 kg/mol DMAEMA-BMA copolymer but not the toxic 15 kg/mol AEMA-BMA copolymer when treated with three different cell lines. This phenomenon was found to persist independently of NLRP3 using NLRP3-KO cells. Time lapse videos can be found in Movies S1–S3 and Figures S13, S15, and S16. For the image denoted with “&”, *t* = 40 min as all cells died and imaging was stopped. The scale bar is representative of all images.

After validating the activity of the polymers in primary cells, time-lapse microscopy was used to observe the polymer–cell interactions that mediated the immunogenic and toxic responses. Polymer-induced changes to organelle pH, lysosomal rupture, or cell membrane integrity could all play roles in toxic or immunogenic responses. Lysosomal rupture is a well-documented component of NLRP3 inflammasome activation and various cell death pathways, yet not all toxic polymers in the data set were found to induce inflammasome activation.^17−20^ To probe the mechanisms behind observed immunogenicity and toxicity, THP-1 cells were treated with phorbol 12-myristate-13-acetate (PMA) to induce an adherent, macrophage-like phenotype and provide stability for time-lapse imaging.^55^ DND-189 was then used to probe lysosomal pH.^59^ PMA-differentiated THP-1 cells were primed with LPS, stained with DND-189, treated with the indicated polymers, and imaged for 1 h in 2 min intervals (Figures 4 and S13). No changes in lysosomal pH were found in live cells prior to death; however, large differences in cellular morphology upon death were observed in cells treated with polymers that induced noninflammatory cell death relative to those that induced inflammatory cell death. The 60 kg/mol DMAEMA_70_-BMA_30_ polymers both swelled and formed blebs upon death (consistent with NLRP3-mediated pyroptosis^20^), whereas 15 kg/mol AEMA_70_-BMA_30_ polymers did not swell upon death, instead undergoing nonspecific lysis (Figure S14). The polymers that swelled upon death were the same polymers that induced IL-1β production in the preliminary screen, suggesting that this lysis could be related to osmolytic lysis and inflammasome activation.

To test whether this result occurred due to polymer–cell interactions or NLRP3-driven pore formation, the experiment was repeated in NLRP3-deficient THP-1 cells (Figures 4 and S15). Similar blebbing was observed for the wild-type THP-1 cells, supporting the hypothesis that differences in polymer–cell interactions underlie the cell death observed in our high-throughput screen. Finally, the assays were repeated in A549, HeLa, and murine BMDCs to evaluate the translation of these results to different cell lines and species, which have different lysosomal characteristics. Similar swelling phenomena were observed in all cell lines (Figures 4 and S16), demonstrating that swelling is a consistent feature of cells treated with hydrophobic, high*-M*_n_ polymers. The observation that cell swelling and inflammatory death are independent of changes to lysosomal pH was further validated using HEK-293T cells containing a genetically encoded biosensor for lysosomal pH called FIRE-pHLy.^42^ When HEK FIRE-pHLy cells were treated with the eight polymers of interest, and the pH was analyzed via flow cytometry 1 h later, no significant changes in lysosomal pH were observed in live cells prior to death (Figure S17). With these results, we conclude that polymers do not directly modulate lysosomal pH and therefore induce osmolytic swelling through alternative means. We therefore explored alternative mechanisms which could explain differences in polymer-induced lysis by different classes of polymers.

### Cationic Polymers Disrupt Cell Membranes to Induce Cell Death and Enter the Cytosol for Immunogenic Responses

**Figure 5:**
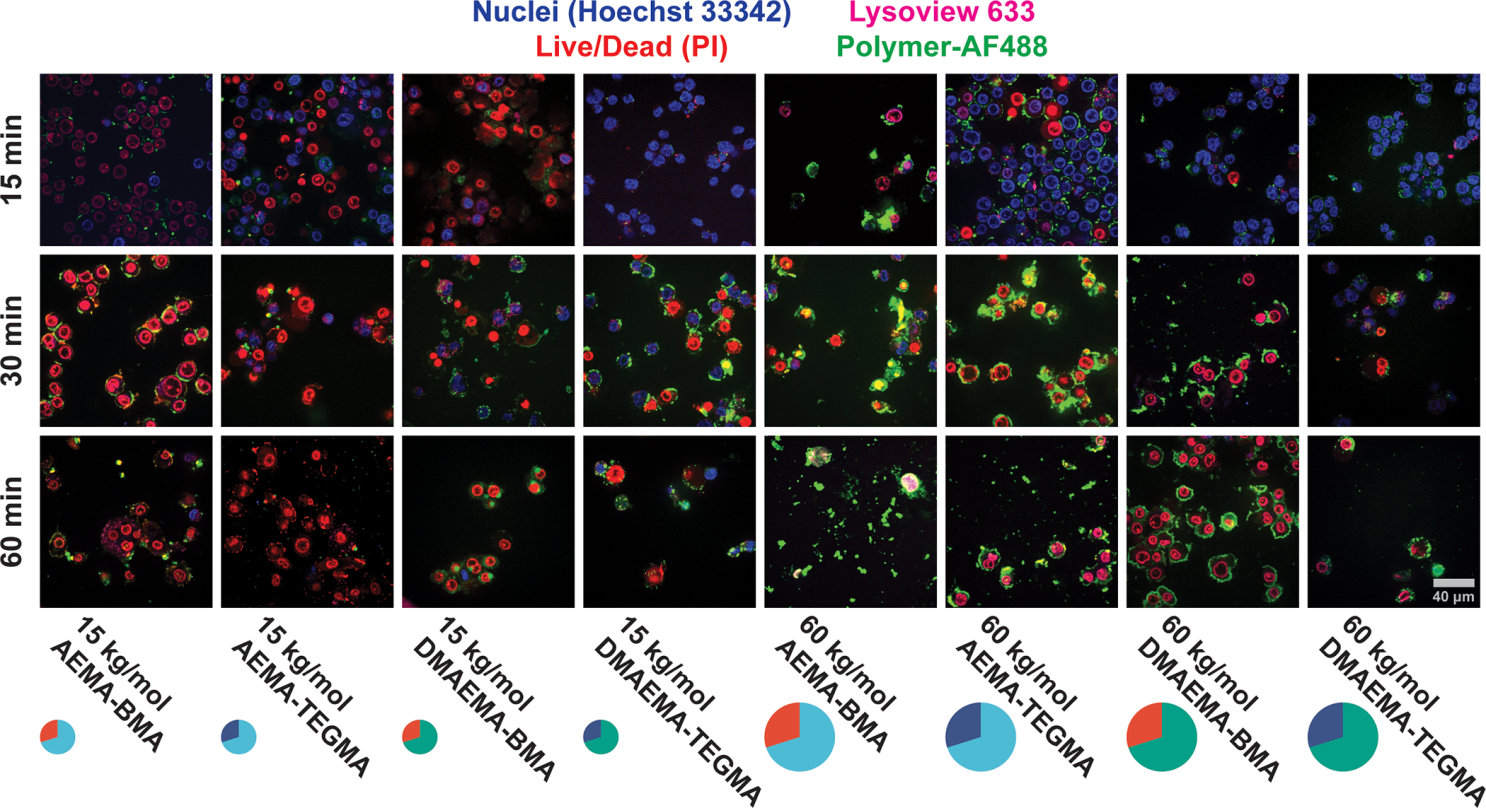
Analysis of cationic polymer localization in the cell for 8 representative polymers. Treatment of THP-1 cells with AF488-labeled polymers was employed to determine cellular localization of polymers at 15, 30, and 60 min after treatment with cells. Cells were LPS primed, stained with the indicated dyes, treated with 100 μg/mL polymers for the indicated times, and then washed and immediately imaged using a confocal microscope (40× oil lens). The scale bar is representative of all images.

Having observed that DND-189-stained THP-1 cells treated with polymers induced cell swelling responses in a manner dependent upon the polymer composition, it was important to identify where polymers with different compositions localize in the cell. To do so, fluorescently tagged versions of the eight polymers of interest (Figure S18) were generated. PMA-differentiated, LPS-primed THP-1 cells were then treated with the fluorescently tagged polymers for 15, 30, and 60 min, washed to reduce the background AF488 signal, and imaged by confocal microscopy (Figure 5). Toxic polymers (e.g., 15 kg/mol of AEMA_70_-BMA_30_) did not enter the cell and instead adhered to the cellular plasma membrane. In contrast, high molecular weight, hydrophobic copolymers such as 60 kg/mol DMAEMA_70_-BMA_30_ entered the cell prior to rupture. Finally, there were differences in the rate of uptake, as highly toxic 15 kg/mol AEMA_70_-BMA_30_ copolymers induced toxicity after only 15 min of treatment. It should be noted that ∼50% of cells died after treatment with the nontoxic, 15 kg/mol DMAEMA_70_-TEGMA_30_ copolymer, which stands in contrast to Figure 4 and is likely an artifact of the imaging environment in this study. These data suggest that differences in immunogenic or toxic behavior could be explained by polymer–cell interactions independently of aggregation propensity in cell culture media. Highly charged polymers disrupt the cellular plasma membrane to induce toxic responses, while less charged, hydrophobic polymers enter the cell and disrupt internal endolysosomal membranes to induce immunogenic responses.

**Figure 6:**
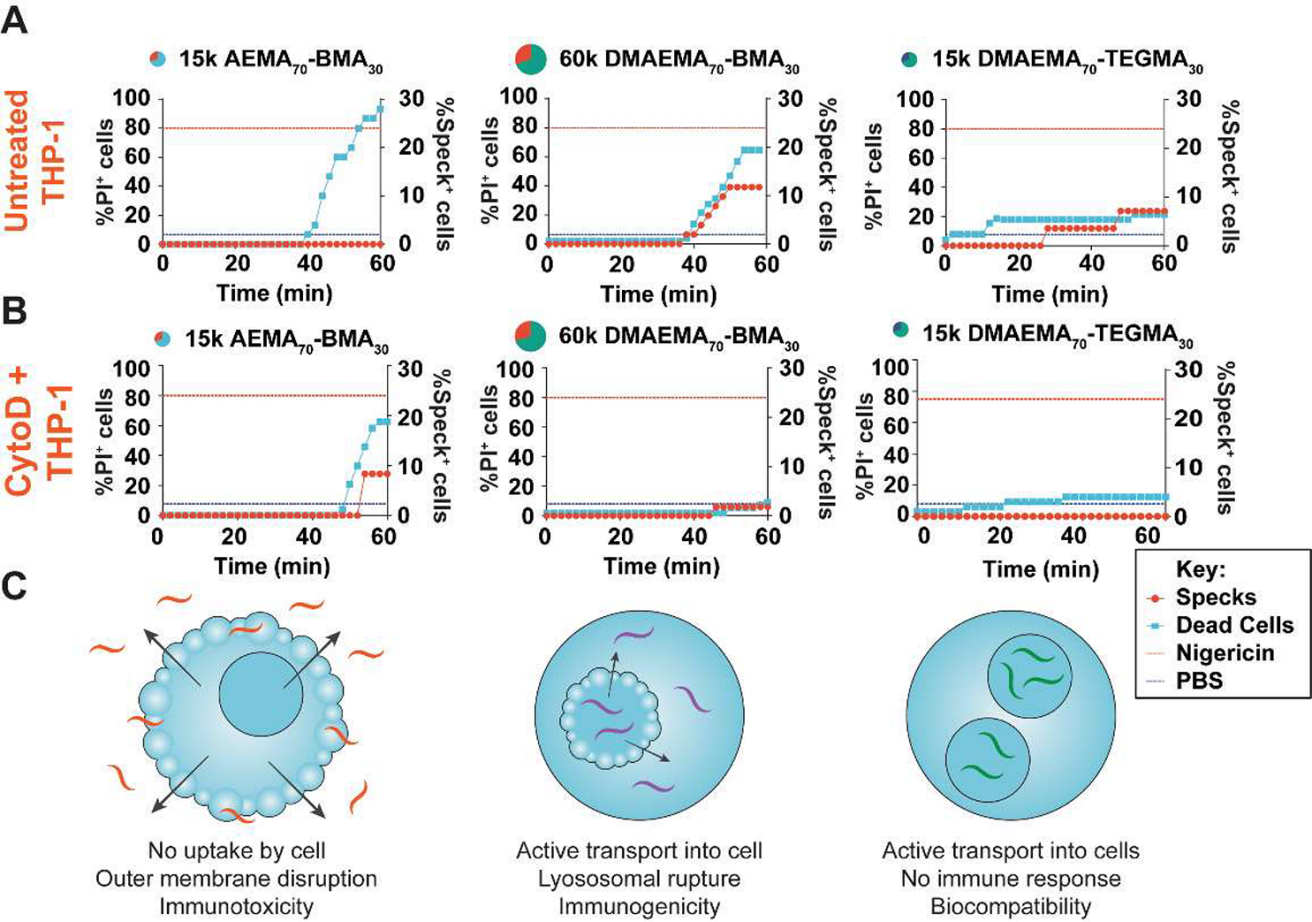
Probing the role of active transport in inflammasome activation induced by representative polymers. THP-1 ASC-GFP cells were treated with (A) the indicated polymers or (B) the indicated polymers + cytochalasin D, and cell death and ASC speck formation were evaluated as a function of time and plotted as shown. Time lapse videos can be found in Movies S4–S5 and Figures S18–S21. (C) Models of cell–polymer interactions for each of the three classes of polymers identified in this study are depicted.

With some possibilities eliminated and a clear set of biological phenomena identified, the next step was to evaluate whether the observed cell–polymer interactions corresponded to IL-1β and LDH secretion identified in the primary screen. To do so, we used THP-1 cells that contain a green fluorescent protein-labeled ASC protein (THP-1 ASC-GFP). Upon inflammasome activation, cytosolic ASC-GFP condenses into “specks” which can be identified visually by confocal microscopy.^60^ THP-1 ASC-GFP cells were primed, stained with propidium iodide (PI), and treated with polymers immediately prior to the onset of imaging. Speck-containing and PI^+^ cells were observed over 2 min intervals for 1 h (Figures 6A and S19–S20). Treatment of the cells with 60 kg/mol DMAEMA_70_-BMA_30_ and 60 kg/mol AEMA_70_-BMA_30_ resulted in speck formation in ∼20% of cells (compared to 24% of cells treated with 5 μM nigericin as a positive control), while cells treated with 15 kg/mol AEMA_70_-BMA_30_ and AEMA_70_-TEGMA_30_ polymers induced toxicity in the absence of specks. While a few specks (<10%) were observed in the nontoxic, nonimmunogenic polymers, these results likely occur due to stressors from the imaging environment (though this was mitigated as best as possible by using an environmental control chamber) and can result from activation of other cell death pathways which also use ASC.^61^

The above results raise the question of whether the inflammasome activation was the result of conventional phagocytic pathways or transcytosis across the cell membrane, as was previously reported for acetylated PEI dendrimers.^58^ To test this hypothesis, the assay was repeated in the presence of cytochalasin D (CytoD), an inhibitor of actin polymerization that prevents active transport into the cell. This assay probed whether active transport into the cell was necessary for inflammatory or toxic responses. In the presence of CytoD, ASC speck formation was almost completely inhibited, providing strong evidence that active transport of polymers into the cell is required for NLRP3 inflammasome activation (Figures 6B and S21–S22). Moreover, cell death in the presence of CytoD was unaffected, except in the case of 60 kg/mol of DMAEMA_70_-BMA_30_ polymers that are known to induce inflammasome activation. In accordance with polymer-AF488 tracking studies, this result suggests that polymers induce a toxic phenotype by membrane disruption in an active transport-independent fashion. Taken together with our IL-1β, LDH, and cell swelling analyses, we conclude that large, hydrophobic polymers induce NLRP3 inflammasome activation and cell death via swelling and rupture of membrane organelles, such as lysosomes. Meanwhile, small, highly charged polymers induce necrotic death by alternative means such as direct membrane disruption (Figure 6C). An open question that remains is the mechanism of active transport into the cell, as PEI-based systems undergo different mechanisms of endocytosis or transcytosis based on their physicochemical properties.^58^ These results provide mechanistic insight into the behavior of cells that are treated with cationic polymers and could inspire rational design of new polymers for a breadth of applications.

### Immunogenic and Immunotoxic Phenotypes Can Be Observed *In Vivo*

**Figure 7:**
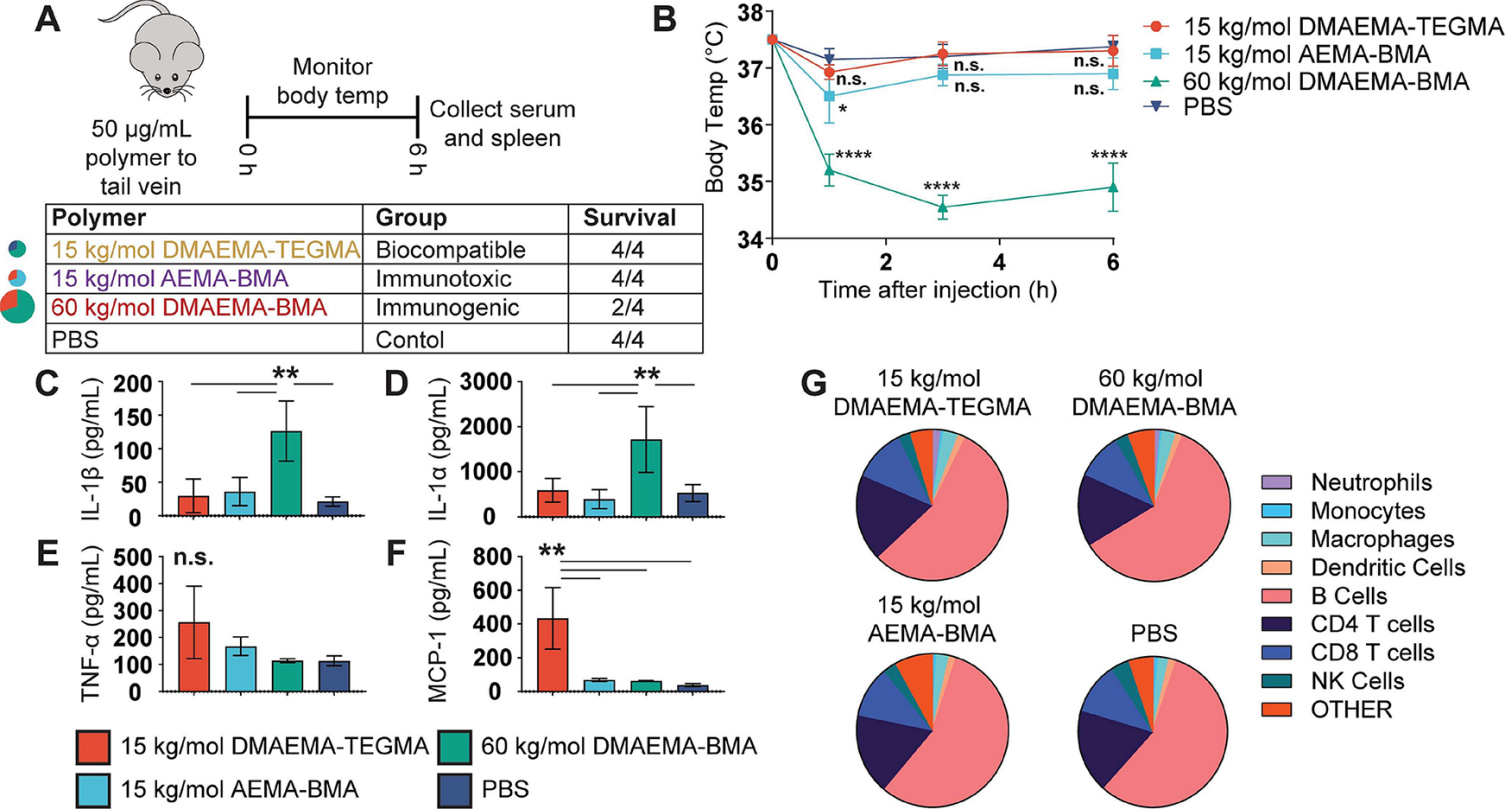
*In vivo* model of immunotoxicity induced by representative polymers. (A) Experimental paradigm for immunotoxicity study and survival after injection. (B) Body weight was monitored 1, 3, and 6 h after injection for mice treated with each polymer. (C–F) Serum cytokines assayed 6 h after injection of polymers was determined via multiplex cytokine panel. (G) Spleen leukocyte composition (% of live CD45^+^ cells) averaged for mice treated with each polymer. For (B), statistics were conducted using one-way ANOVA with Tukey’s multiple comparisons test relative to PBS. For (C–F), an analogous test was performed, where all of the multiple comparisons were queried.

Having conducted this extensive screen and mechanistic analysis, the next goal was to demonstrate the functional relevance of these data to biological applications. To probe the immunotoxic behavior of inflammasome-activating polymers *in vivo*, three polymers representing the classes of materials described in Figure 6C were administered intravenously to the tail vein to model studies by Tahtinen et al. and others.^15,23,62^ 15 kg/mol AEMA_70_-BMA_30_ (15 kg/mol) represents a toxic, membrane-rupturing polymer, 60 kg/mol DMAEMA_70_-BMA_30_ represents an immunogenic, lysosome-rupturing polymer, and 15 kg/mol DMAEMA_70_-TEGMA_30_ represents a nontoxic, biocompatible polymer. Immune responses toward the polymers and IL-1β mediated toxicity were measured including cytokine production, body temperature, and splenic immune responses. (Figure 7A). Recent reports on mRNA liposome and lipid nanoparticle vaccine formulations, bearing charged amines indicated that IL-1 was a key mediator of immunogenicity and reactogenicity toward such formulations in a composition-dependent manner. It was hypothesized that the physicochemical properties of these polymers could similarly bias the immune response and inspire design principles for future therapeutics.

After injection of 50 μg of each polymer, mice (*n* = 4/group) treated with 15 kg/mol AEMA_70_-BMA_30_ or 15 kg/mol DMAEMA_70_-TEGMA_30_ did not exhibit changes to body temperature or other adverse health outcomes. On the other hand, two mice treated with 60 kg/mol DMAEMA_70_-BMA_30_ died within 1 h after injection, possibly a result of cytokine storm or aggregation *in vivo*, and the remaining mice showed a large decrease in body temperature indicative of a sickness response (Figure 7B). After 6 h, all remaining mice were sacrificed. Cytokines in the sera were evaluated, and it was observed that mice treated with 60 kg/mol DMAEMA_70_-BMA_30_ had significant increases in systemic IL-1α and IL-1β (Figure 7C,D). Surprisingly, this increase in IL-1 cytokines was not accompanied by increases in other proinflammatory cytokines such as TNF-α and MCP-1 (Figures 7E,F), which were found to be increased in mice treated with the nonimmunogenic, 15 kg/mol DMAEMA_70_-TEGMA_30_ polymers. This TNF-α and MCP-1 cytokine profile is possibly a kinetic phenomenon resulting from improved biodistribution of the hydrophilic polymers. A nonsignificant increase in spleen size was observed in mice treated with 60 kg/mol DMAEMA_70_-BMA_30_, although there were no changes in leukocyte composition (Figures 7G and S23–S24). When intracellular cytokine staining was conducted on leukocytes for IL-1α, pro-IL-1β, and TNF-α, a few differences between groups were observed (Figure S25). Notably, mice treated with 15 kg/mol DMAEMA_70_-TEGMA_30_ had an increase in IL-1α^+^ macrophages, which suggests that these polymers could more effectively traffic to the spleen for endocytosis, but more work must be conducted to formulate these materials for optimized delivery. Overall, these results confirm that hydrophobic polymers containing tertiary amines, such as 60 kg/mol DMAEMA_70_-BMA_30_, can induce IL-1 cytokine production via NLRP3 inflammasome activation both *in vitro* and *in vivo*. IL-1 can be productive or detrimental, depending on context, necessitating careful control over biodistribution of these materials.^23^ Understanding the polymeric properties that induce systemic immunotoxic responses is a central requirement for the design of safe, next-generation biomaterials.

## Conclusion

Polymers have emerged as key components of many biotechnologies. For the safe and effective implementation of polymers for these technologies, better methods to rapidly probe their characteristics, such as charge or hydrophobicity, and determine how they affect immunological and/or toxic responses are needed.^9^ Here, we show that physicochemical characterization of a polymeric library in tandem with *in vitro* high-throughput screening can elucidate immunogenic and toxic responses emergent from polymers’ compositions. Our model system was prepared using a process chemistry robot to copolymerize one of two amine-containing monomers, AEMA and DMAEMA, with one of two hydrophobicity altering monomers, BMA and TEGMA, in a parametric fashion via RAFT. Using a screening approach based on IL-1β and LDH secretion, we generated a structure–property map of 107 water-soluble, cationic polymers representative of materials used in drug delivery systems, antimicrobial coatings, and tissue scaffolds. We then conducted microscopy to identify a mechanistic basis for the observed responses. With these microscopy experiments, we observe that classes of polymers with different IL-1β and/or LDH secretion profiles correspond to underlying differences in polymer–cell interactions (Figure 6C). Finally, an *in vivo* biocompatibility study was undertaken, where we showed that it is possible to extrapolate the *in vitro* results from the high-throughput screening data set to *in vivo* responses with reasonable fidelity. These results can inform the development of future cationic biomaterials.

Overall, this study provides three key insights: (1) the correlation of immunogenicity and toxicity with polymer–cell interactions, (2) a “map” of the composition of polymers that result in these interactions, and (3) a simple and low-cost workflow to identify immunotoxic behavior of a polymer prior to *in vivo* testing. First, we correlated immunogenicity and toxicity induced by polymers with underlying polymer–cell interactions. Baljon et al.^18^ recently reported that the ratio of DMAEMA to BMA in a polymer can modulate NLRP3 inflammasome activation. Adding to this finding, we report that a key mechanistic feature behind this phenomenon is the polymer’s ability to enter the cell. Hydrophobic, high-*M*_n_ polymers (i.e., those ≥45 kg/mol and containing DMAEMA and >20 mol % BMA) displayed robust inflammasome activation in an active transport-dependent fashion. These results strongly suggest that polymers’ entry into the cell is the determining factor for NLRP3 inflammasome activation. Polymers that are actively transported into the cell enter the more acidic endolysosome (pH ∼ 5.5–6.5), where they likely become highly charged and induce membrane disruption. On the other hand, highly charged, low-*M*_n_ polymers (i.e., those ≤30 kg/mol and composed of ≥50 mol % AEMA) induced toxicity via external plasma membrane disruption. Finally, less charged, hydrophilic polymers (i.e., those containing DMAEMA and ≥20 mol % TEGMA) were taken up to a lower extent but did not disrupt membranes and were therefore biocompatible at all concentrations tested. One open question that remains is the mechanism of polymer-induced membrane and organelle disruption. This question is of great interest and beyond the scope of this study, as several pore-forming mechanisms have already been proposed and studied.^54,58^

Second, we provide a “map” of cationic polymers’ immunogenic and toxic behavior. By mapping the response generated by cells treated with 107 polymers varied over several dimensions, we find that subtle variations in a polymer’s properties can have large effects on immunogenicity and toxicity. These effects can be attributed to changes in the proportional ratio between the hydrophobicity and charge in a polymeric scaffold. Polymers containing 10–20 mol % of a hydrophobic monomer (BMA) within an amine monomer backbone (AEMA or DMAEMA) were among the most toxic tested, inducing cell death at all concentrations tested. Those with any proportion of AEMA or BMA tested were toxic at higher concentrations. High-*M*_n_, DMAEMA-containing polymers with ≥20 mol % of BMA activated the NLRP3 inflammasome upon active transport into the cell. Copolymers of DMAEMA and BMA are highly effective gene delivery platforms,^31,32^ so reducing *M*_n_ or BMA content to avoid immunogenic side effects may serve as a valuable tool for safe translation of these systems. Finally, copolymers of DMAEMA and TEGMA were found to be nontoxic and nonimmunogenic, particularly when low *M*_n_ (≤30 kg/mol) and/or >20 mol % TEGMA was employed. These polymers have lower p*K*_a_s combined with high degrees of charge shielding conferred from ethylene glycol moieties, which previous studies have shown to prevent rupture of membranes and facilitate safe, intracellular delivery when complexed with RNA.^57,63^ Overall, these results provide “points of no return” of polymer composition that, when avoided, can ensure that an amine-containing polymer will have a low probability of being immunotoxic. They provide a starting point to accelerate polymer-based therapeutic development, although we acknowledge that structure–function optimization will be needed for some applications. The tuning of subtle and conflicting parameters can be crucial in determining the most effective materials for a specific application, though we note that modern computational tools should aid in selection.^40^ For example, high *M*_n_s of DMAEMA-containing polymers are critical in maximizing transfection efficiency, though these properties may also yield increased rates of inflammasome activation and inflammatory cell death.^64^ With that said, the results herein can dramatically reduce the design space of interest to quickly identify materials for a breadth of applications.

Third, and perhaps of greatest importance, we present a simple method to screen the immunogenicity and toxicity of polymers prior to costly *in vivo* assays. While highly specialized, application driven readouts are appealing to maximize the effectiveness of a material for a given application; they are poorly translated between different fields and often fail due to unanticipated biocompatibility challenges. While this study focused on IL-1β and LDH release induced by cationic polymers, the workflow used herein can be applied toward other markers of immunotoxicity, such as TNF-α, IL-1α, or reactive oxygen species. It can also be applied to different polymer properties. Applying these insights in new and existing therapeutics should streamline (pre)clinical testing to design tailored materials for applications in biomedicine. By using common monomers with general features, such as primary or tertiary amines, we sought to make this initial “map” general to many common applications of cationic polymers. In verification studies, branched PEI fell within the toxicity and immunogenicity parameters outlined by DMAEMA and BMA (Figure S12). While the information generated from our screen may not be general to every cationic polymer, we suggest it will provide a robust starting point for any application driven study (e.g., designing polymers for mRNA delivery). As structure–property information is obtained on additional polymers, high-throughput screening can be combined with machine learning to unveil emergent properties and develop newer, better materials.^40^

Many studies have used structure–function information to identify polymers for biological applications without recognizing the inherent compromise among functionality, immunogenicity, and toxicity. A combination of physicochemical properties, such as charge, hydrophobicity, or molecular weight, that leads to a desired response can behave as a double-edged sword, causing lysosomal rupture and inflammasome activation or cell necrosis. Understanding the polymer–cell interactions that govern these responses can accelerate polymer development and reduce late-stage biocompatibility screening failures. By varying parameters in a systematic fashion, we present a single parametrized map rather than disparate outputs generated in application-driven studies. Using the screening data presented in this work, researchers can make informed choices and design polymer compositions that might be suitable for a variety of applications. For example, in drug delivery, small quantities of positive charge in a polymer can improve performance (e.g., using a primary amine comonomer to facilitate protein adsorption), but too much positive charge might result in toxicity. This study suggests a small quantity (<10 mol %) of a primary amine containing monomer (such as AEMA) could be accommodated by applying the heuristics outlined herein to build polymers which balance the charge with hydrophilic, ethylene glycol-containing components. Overall, the screening strategies and mechanistic results in this study provide a framework from which future materials can be built, accelerating the development of new biomaterials.

## Supporting Information Available

Additional methods, table of antibodies, synthesis of BocAEMA, full synthetic details (^1^H NMR and SEC), and screening results of all polymers, additional characterization of selected polymers (monomer consumption rates, DOSY-NMR, DLS, acid/base titration), screening results in BMDCs and PBMCs, immunogenicity and toxicity of PEI, HEK FIRE-pHLy studies, time-lapse images from microscopy, additional flow cytometry from *in vivo* study, and description of Movies S1–S5 (PDF)
Movies S1–S5 (ZIP)
