# Supplementary material for: Understanding How Cationic Polymers’ Properties Inform Toxic or Immunogenic Responses via Parametric Analysis
Source: Macromolecules. 2023 Sep 8;56(18):7286–99. doi: 10.1021/acs.macromol.3c01223 (PMC10537447; doi:10.1021/acs.macromol.3c01223)
Supplement: Supplementary file 1 — ma3c01223_si_001.pdf [file ma3c01223_si_001.pdf]

Supplementary Information For:

Understanding How Cationic Polymers' Properties Inform Toxic or Immunogenic Responses *Via* Parametric Analysis

Adam M. Weiss[1,2,&], Marcos A. Lopez II,[2] Benjamin W. Rawe,[1] Saikat Manna,[1] Qing Chen,[1] Elizabeth J. Mulder,[1] Stuart J. Rowan,[1,2,3] Aaron P. Esser-Kahn[1,3]

[1] Pritzker School of Molecular Engineering, University of Chicago
5640 S Ellis Ave., Chicago, IL 60637, USA

[2] Department of Chemistry, University of Chicago
5735 S Ellis Ave., Chicago, IL 60637, USA

[3] Co-corresponding authors: aesserkahn@uchicago.edu, stuartrowan@uchicago.edu

[&] Current address:
Center for Translational Medicine, University of Montana
32 Campus Drive, Missoula, MT, 59801, USA

Keywords: Biomaterials, Polymers, Immunology, Biophysics, Cell-Material Interactions

Table of Contents

Pg S2.	Table of Contents
Pg S3-S5.	Supplementary Methods
Pg S7.	Table of Antibodies Used for Flow Cytometry Studies
Pg S8.	Figure S1: Synthesis of 2-(N-(tert-butoxycarbonyl)amino)ethyl methacrylate
Pg S9-S12.	Figure S2: Analysis of Monomer Consumption Over Time for Polymerizations
Pg S13-S15.	Figure S3: Polymer Characterization Data Table
Pg S16-S17.	Figure S4: NMR Diagnostic Peaks for Polymer Analyses
Pg S18.	Figure S5: DOSY-NMR of Selected Polymers and Comparison to GPC
Pg S19.	Figure S6: DLS and TEM of Selected Polymers for Aggregation Analysis
Pg S20.	Figure S7: Titration and pK_a Value of Selected Polymers
Pg S21.	Figure S8: Effect of Polymers on pH of Cell Culture Media
Pg S22.	Figure S9: Full Results of In-Vitro Screening
Pg S23.	Figure S10: Polymer-Induced LDH and Cytokine Production in MoDCs
Pg S24.	Figure S11: Polymer-Induced LDH and Cytokine Production in BMDCs
Pg S25.	Figure S12: In-vitro Screening of Branched Poly(ethyleneimine)
Pg S26.	Figure S13: Raw Images of Lysosensor Green Assay in WT THP-1 Cells
Pg S27.	Figure S14: Cell Swelling of THP-1 Cells
Pg S28.	Figure S15: Raw Images of Lysosensor Green Assay in NLRP3-KO THP-1 Cells
Pg S29-S30.	Figure S16: Raw Images of Lysosensor Green Assay in other cell lines
Pg S31.	Figure S17: HEK FIRE-pHLy Lysosomal pH Assay
Pg S32.	Figure S18: Synthesis and Characterization of AF488-Labelled Polymers
Pg S33.	Figure S19: THP-1 ASC-GFP Speck Formation
Pg S34.	Figure S20: THP-1 ASC-GFP Speck Raw Images
Pg S35.	Figure S21: THP-1 ASC-GFP Speck Formation + Cytochalasin D
Pg S36.	Figure S22: THP-1 ASC-GFP Speck Raw Images + Cytochalasin D
Pg S37.	Figure S23: Flow Cytometry Gating Strategy for I.V. Polymer Administration
Pg S38.	Figure S24: Absolute Cell Counts in Spleen after I.V. Polymer Administration
Pg S39.	Figure S25: Intracellular IL-1 and TNF-α in Spleen after I.V. Polymer Administration
Pg S40-S60.	Raw NMR Spectra and SEC Traces for Polymeric Library
Pg S61-S62.	Descriptions of Supplementary Videos
Pg S62.	Supplementary References

Supplementary Methods and Materials

Analysis of Monomer Consumption

To confirm the random incorporation of monomer into the polymer backbone, polymerizations were subjected to ^{1}H-NMR spectroscopy using a 60-to-40 mol.% ratio of AEMA or DMAEMA to BMA or TEGMA and M_n = 30 kg/mol target. Purified monomers, CTA, ACVA, and a stir bar were added to a flame-dried flask for a 1.0 g scale reaction. The flask was sealed, 2.5 mL dry DMF was transferred by syringe, and the reaction sparged with argon for 30 min. The flask was then added to a pre-heated oil bath at 72 °C and stirred. At 1, 2, 4, 6, and 18 h, 50 µL aliquots of crude reaction mixture were collected, quenched by rapid addition to an ice bath, and diluted in 600 µL DMSO-*d6*. The mixtures were analyzed by ^{1}H-NMR spectroscopy to evaluate relative consumption of monomers as well as the reaction conversion. The ^{1}H-NMR spectra in DMSO-*d6* were referenced to a TMS standard and analyzed in MestReNova. After 18 h, the reaction mixtures were quenched by exposure to air, deprotected, and purified as described in the **Methods and Materials**. The aerogels were then re-analyzed by ^{1}H-NMR spectroscopy in D_2O to confirm that molar ratios of polymers were consistent with expected molar ratios of AEMA or DMAEMA to BMA or TEGMA. Monomer consumption over time for each co-monomer system were conducted at least twice to confirm the results shown. The results shown are representative of the replicate trials.

Diffusion Ordered Spectroscopy-Nuclear Magnetic Resonance (DOSY-NMR)

Selected samples were analyzed using a 500 MHz Bruker Avance-II+ spectrometer equipped with a QNP probe using Topspin 2.1. Samples were analyzed using the ledbpgps2 pulse program with P30 = 1900 µs, Δ20 = 1 s, and D1 = 5 s, and 32 ramp increments (16 scans/spectrum, linear gradient) ranging from a gradient amplitude (GPZ6) of 5-95% were obtained for each sample. Diffusion analysis was subsequently conducted using MestreNova, and the diffusion constant was calculated by determining the largest non-solvent peak from each sample, plotting the integral

intensity as a function of field strength, and fitting the resultant curve to a mono-exponential fit function y = B*Exp(-x*F), where F is the diffusion constant.

Dynamic Light Scattering

Dynamic light scattering (DLS) measurements were performed by a Wyatt Mobiuζ DLS instrument. Selected polymers were dissolved at 1 mg/mL in phenol-red free RPMI + 10% FBS. Measurements were performed at 25 ^{o}C or 37 ^{o}C using a laser wavelength of 532 nm. Scattered light was collected at a fixed angle of 163.5^{o}.

Transmission Electron Microscopy

Transmission Electron Microscopy (TEM) was performed using a FEI Tecnai F30 300 kV FEG(s) TEM microscope. Carbon-coated copper grids were treated with oxygen plasma before deposition of the samples. The samples were deposited on the carbon grids for 1 min, and excess samples were wicked away. The samples were allowed to dry under ambient conditions and subsequently stained with uranyl acetate prior to imaging.

Acid-Base Characterization

The pKa of selected polymers was determined by acid-base titration. Briefly, selected polymers (70:30 ratio, 15 kg/mol, 30-50 mg per experiment) were dissolved at 1 mg/mL in deionized water and adjusted to pH = 4.0. Then, 0.01 M NaOH was added dropwise at a fixed rate, and pH was recorded every 15 s using an electronic pH meter (Hanna Instruments). The pKa was determined by finding the inflection point in the titration curve. To further validate that the acidity/basicity of polymers had no effect on the pH of cell culture medium, 20 μL of selected polymers (1 mg/mL) in dH_2O was added to 180 μL of phenol red-containing cell culture medium in a clear, flat bottomed

96 well plate. Absorbance was collected using a Multiskan FC plate reader (Thermo Fisher), and the 442/570 nm absorbance ratio was used to determine changes to pH.

Monocyte-derived Dendritic Cell Validation Assay

Peripheral blood samples were thawed and isolated by magnetic separation using EasySep Human Monocyte Isolation Kit (StemCell Technologies) according to the manufacturer's protocol. Isolated monocytes were then cultured in RPMI + 10% FBS with IL-4 and GM-CSF supplementation to induce a dendritic cell-like phenotype. After 6 d, the monocyte-derived dendritic cells (MoDCs) were released with EDTA, pelleted by centrifugation, and plated at 90,000 cells/well in RPMI + 10% HI-FBS. Cells were primed with 100 EU/mL ultrapure LPS-EB for 3 h. Media was then removed and replaced with fresh media containing polymer solutions at the indicated concentration. Cells were incubated with polymers for 5 h and then medium was collected and analyzed with human IL-1β ELISA Kit (Thermo Scientific) and CyQUANT LDH Cytotoxicity Assay (Thermo Fisher) according to the manufacturer's procedures.

Bone Marrow Derived Dendritic Cell Validation Assay

Bone-marrow-derived dendritic cells (BMDCs) were harvested from C57Bl/6J or B6.129S6-Nlrp3^{tm1Bhk}/J mice (Jackson Laboratory) and differentiated with GM-CSF to a dendritic cell-like phenotype as previously reported.[S1] After 6 d, cells were released with EDTA, pelleted by centrifugation, and plated at 180,000 cells/well in a 96-well plate in DMEM with 10% HI-FBS, 10 mM HEPES buffer, 55 μM β-mercaptoethanol, 100 U/mL penicillin, and 100 μg/mL streptomycin ("BMDM medium"). After allowing adhesion for 1 h, cells were primed with 100 EU/mL ultrapure LPS-EB for 3 h. Media was then removed and replaced with fresh BMDM medium containing polymer solutions at the indicated concentration. Cells were incubated with polymers for 5 h and then medium was collected and analyzed with mouse IL-1β ELISAMAX Kit (BioLegend) and CyQUANT LDH Cytotoxicity Assay (Thermo Fisher) according to the manufacturer's procedures.

Synthesis of 2-(N-(tert-butoxycarbonyl)amino)ethyl methacrylate (BocAEMA)

BocAEMA was prepared as reported previously with minor changes.[S2] Briefly, 60.0 g di-tert-butyl dicarbonate was dissolved in 100 mL DCM in a 250 mL flask containing a stir bar. The reaction was placed under an addition funnel, pleaced in an ice bath, and sealed under Argon. 25.0 mL 2-aminoethan-1-ol was added to the addition funnel and added dropwise under rapid stirring. After addition was complete, the reaction was warmed to room temperature and stirred for 16 h. The reaction was then extracted sequentially with 100 mL 0.1 M NaOH, 100 mL H_2O, and 100 mL brine, and the organic phase was collected and dried over $MgSO_4$. The solvent was removed under reduced pressure to crude obtain N-(tert-Butoxycarbonyl)ethanolamine, which was used without further purification. 10.0 g of N-(tert-Butoxycarbonyl)ethanolamine was then added to a flame dried flask with 8.0 mL triethylamine and a stir bar, dissolved in 100 mL dry DCM, and sealed under argon. The flask was placed in an ice bath, and 9.0 mL methacryloyl chloride in 50 mL dry DCM was added over 30 min with rapid stirring. Upon complete addition, the reaction was warmed to room temperature and stirred for 16 h. The reaction was then quenched by addition of 100 mL 0.1 M NaOH, and the organic phase was washed with 2 x 100 mL H_2O and 100 mL brine prior to drying over $MgSO_4$. The product was filtered, concentrated under reduced pressure, and recrystallized from 1:1 Hexanes:DCM to obtain 2-(N-(tert-butoxycarbonyl)amino)ethyl methacrylate as a white crystalline solid. ^{1}H-NMR: 6.13 (s, 1H), 5.59 (s, 1H), 4.79 (br s, 1H), 4.21 (t, 2H), 3.45 (q, 2H), 1.95 (s, 3H), 1.45 (s, 9H).

CellProfiler Image Analysis

RGB images collected from confocal imaging as described in the *Methods* were imported to the CellProfiler software (Broad Institute). Images were then converted to greyscale, smoothed, and objects were identified using “Robust Background” thresholding. Area of each cell “object” was then determined, and areas were averaged and plotted as a function of time for each treatment.

Table of Antibodies Used for Flow Cytometry

Antibody	Fluorophore	Clone	Vendor
**Live/Dead Blue**	N/A	N/A	Thermo
**CD45**	APC/Cy7	I3/2.3	BioLegend
**CD11b**	PerCP	M1/70	BioLegend
**CD11c**	BV421	N418	BioLegend
**F4/80**	BV785	BM8	BioLegend
**Ly6G**	PE-CF594	1A8	BioLegend
**CD19**	BUV737	1D3	BD
**Ly6C**	AF700	HK1.4	BioLegend
**NK1.1**	BUV563	PK136	BD
**CD3**	efluor506	17A2	Thermo
**CD4**	BV605	RM4-5	BioLegend
**CD8**	PE/Cy7	53.6-7	BioLegend
**TNF-alpha**	AF488	MP6-XT22	BioLegend
**pro-IL-1beta**	APC	NJTEN3	Thermo
**IL-1alpha**	PE	ALF-161	BioLegend
**CD16/32 (TruStain FcX™)**	N/A	93	BioLegend

Supplemental Results

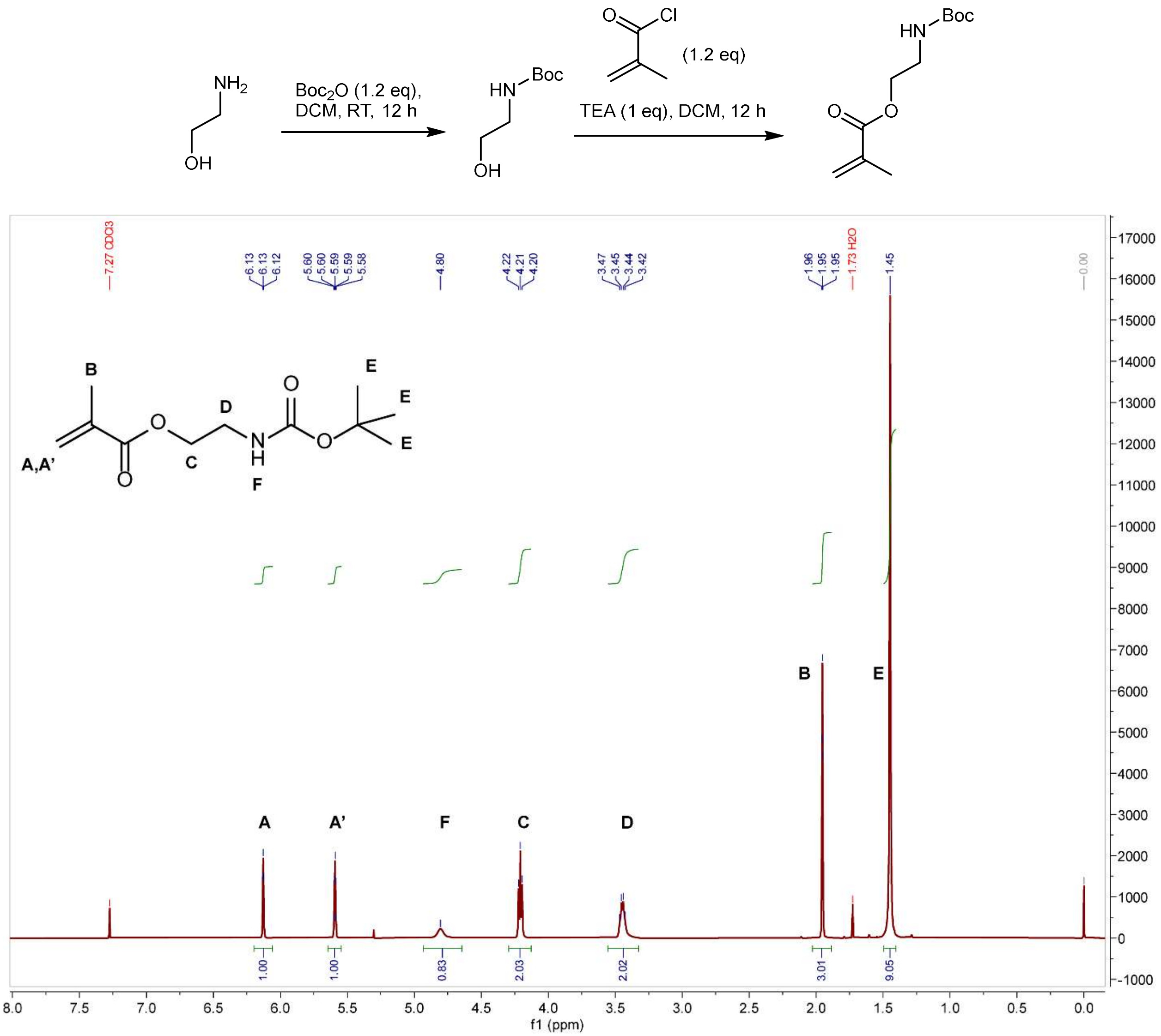

Figure S1: Synthesis of 2-(N-(tert-butoxycarbonyl)amino)ethyl methacrylate. Synthetic scheme and ^{1}H-NMR of 2-(N-(tert-butoxycarbonyl)amino)ethyl methacrylate (BocAEMA) in $CDCl_3$ at 400 MHz.

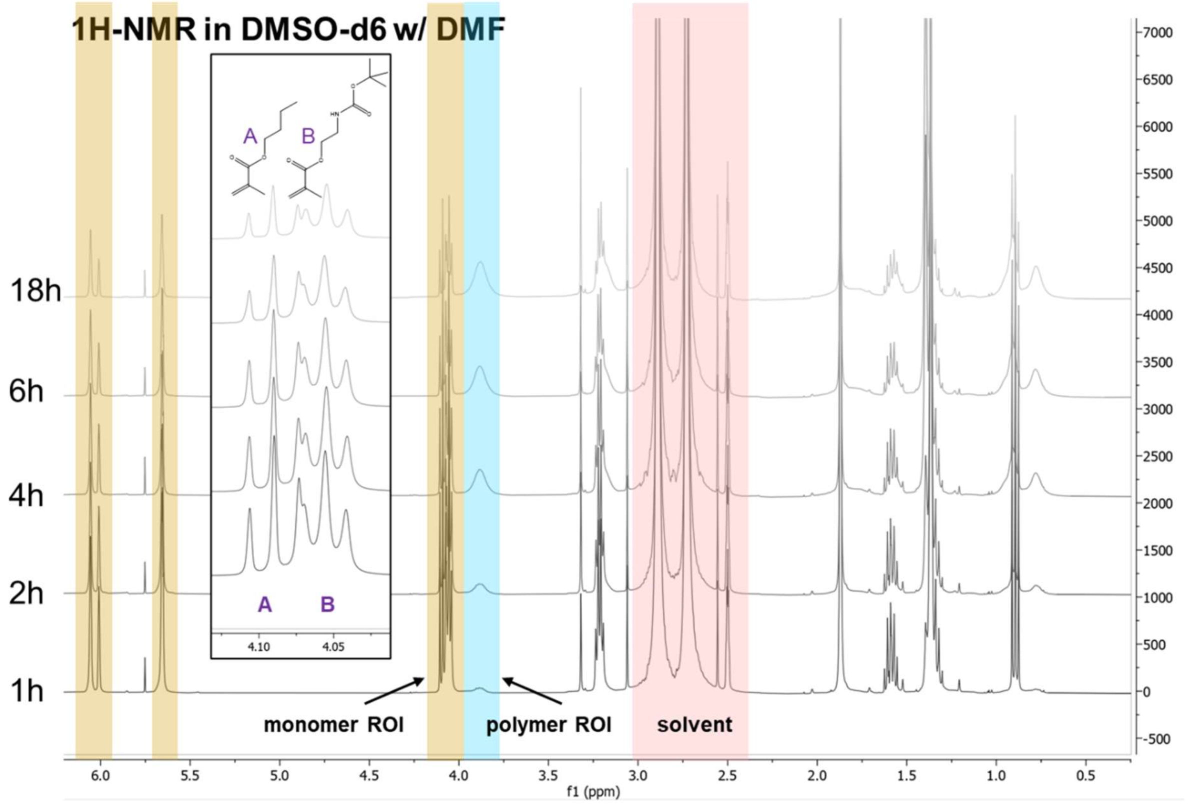

Time (h)	Conversion	%BocAEMA in solution	%BocAEMA in polymer
1	2%	60.0%	N/A
2	10%	61.3%	N/A
4	31%	60.5%	N/A
6	41%	60.6%	N/A
18	56%	63.4%	N/A
Final	N/A	N/A	57.9%

A) Analysis of monomer consumption versus time for a polymer containing 60 mol.% BocAEMA and 40 mol.% BMA was conducted by ^{1}H-NMR using DMF as reaction solvent and DMSO-d6 as a locking solvent for NMR. The indicated regions of interest (ROI) were integrated to determine the conversion of monomer at each time point. The molar ratio of BocAEMA and BMA in solution is stable throughout the reaction indicating that the monomers are incorporated into the polymer with similar kinetics.

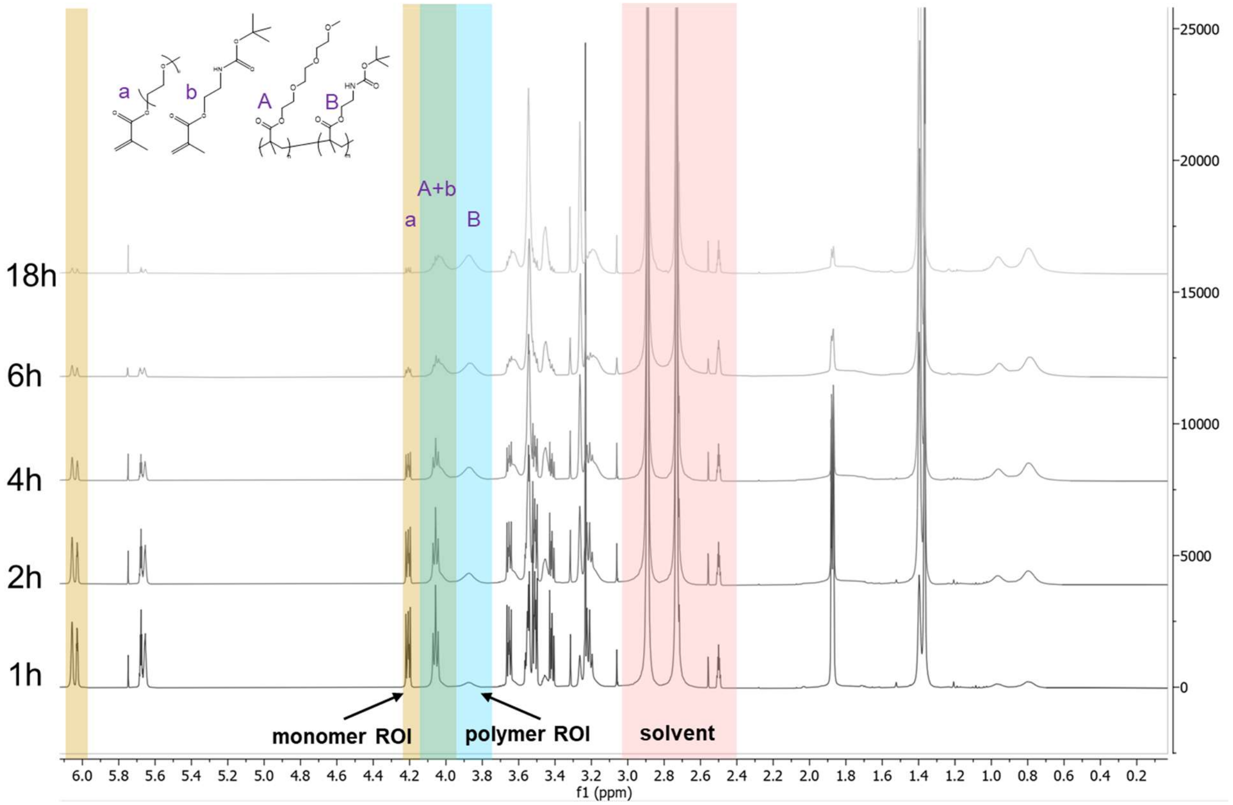

Time (h)	Conversion	%BocAEMA in solution	%BocAEMA in polymer
1	20%	59.5%	N/A
2	45%	59.0%	N/A
4	71%	58.5%	N/A
6	82%	62.5%	N/A
18	96%	58.0%	N/A
Final	N/A	N/A	57.5%

B) Analysis of monomer consumption versus time for a polymer containing 60 mol.% BocAEMA and 40 mol.% TEGMA was conducted by ^{1}H-NMR using DMF as reaction solvent and DMSO-d6 as a locking solvent for NMR. The indicated regions of interest (ROI) were integrated to determine the conversion of monomer at each time point. The molar ratio of BocAEMA and TEGMA in solution is stable throughout the reaction indicating that the monomers are incorporated into the polymer with similar kinetics.

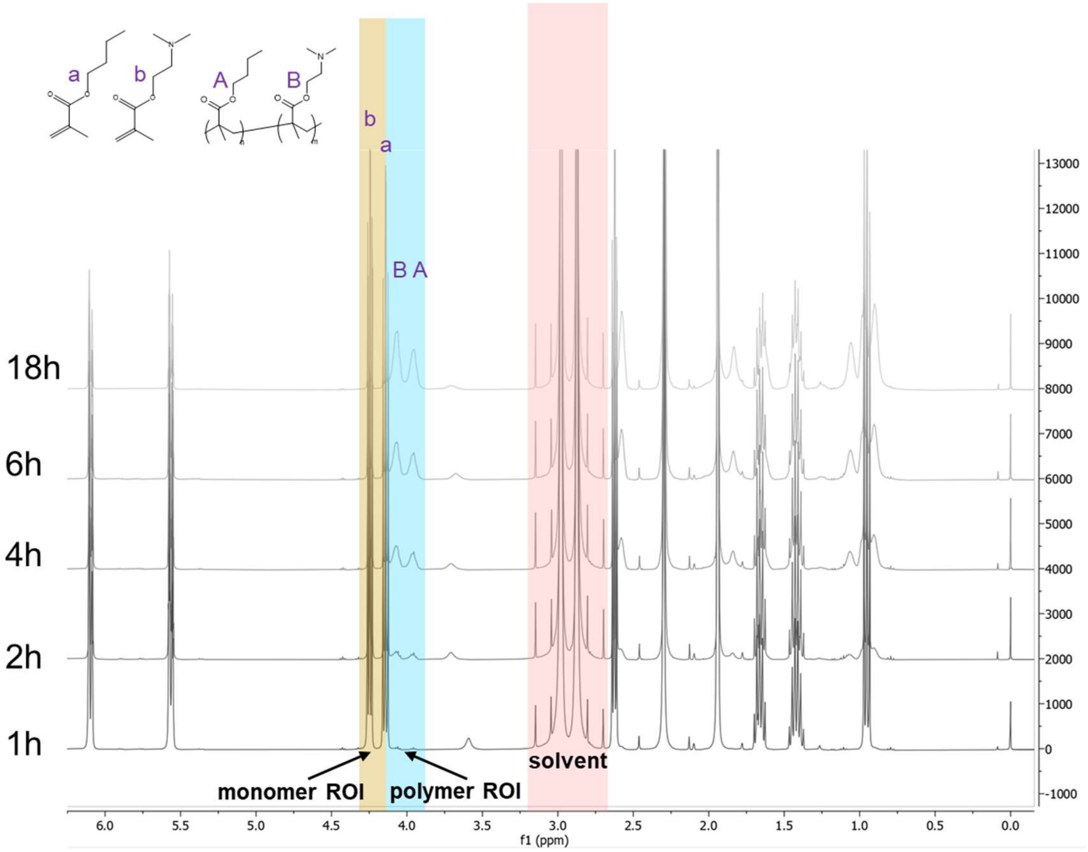

Time (h)	Conversion	%DMAEMA in solution	%DMAEMA in polymer
1	1%	58.1%	N/A
2	7%	57.8%	N/A
4	24%	57.5%	N/A
6	36%	57.5%	N/A
18	58%	57.5%	N/A
Final	N/A	N/A	57.3%

C) Analysis of monomer consumption versus time for a polymer containing 60 mol.% DMAEMA and 40 mol.% BMA was conducted by ^{1}H-NMR using DMF as reaction solvent and DMSO-d6 as a locking solvent for NMR. The indicated regions of interest (ROI) were integrated to determine the conversion of monomer at each time point. The molar ratio of DMAEMA and BMA in solution is stable throughout the reaction indicating that the monomers are incorporated into the polymer with similar kinetics.

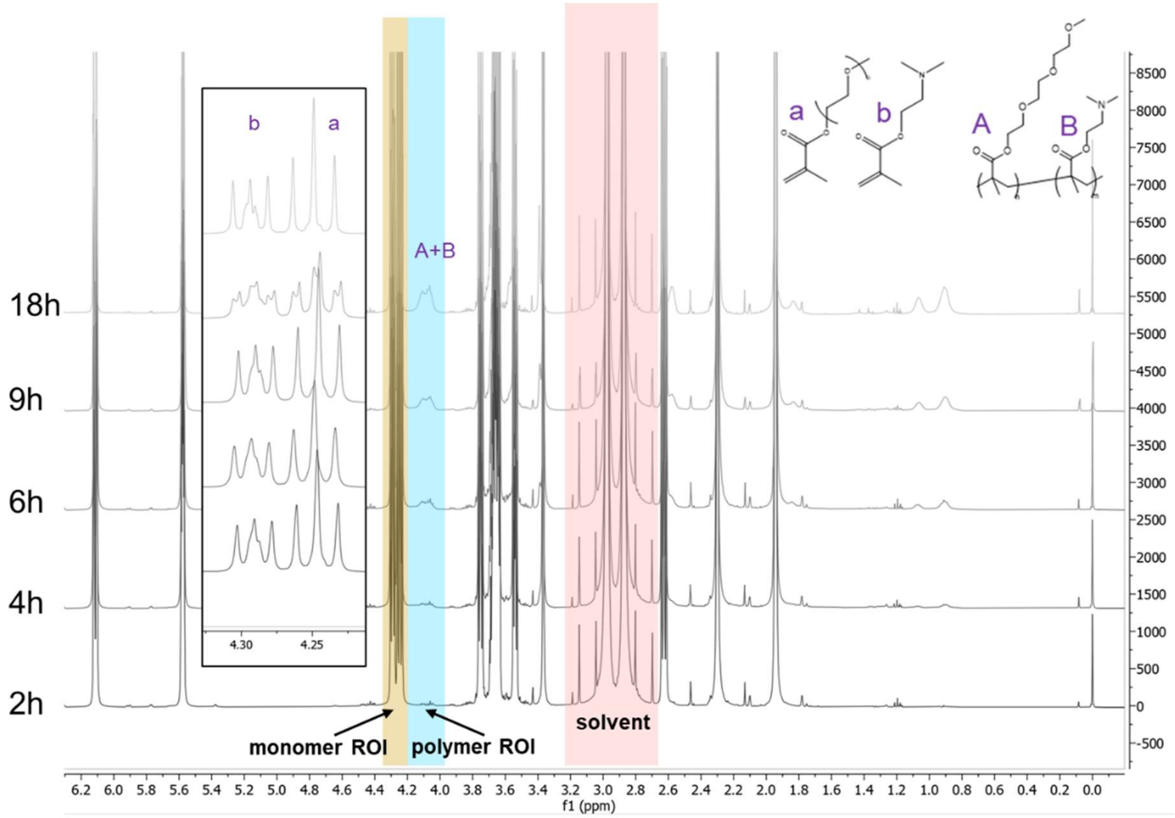

Time (h)	Conversion	%DMAEMA in solution	%DMAEMA in polymer
2	1%	56.7%	N/A
4	3%	56.3%	N/A
6	5%	56.9%	N/A
9	10%	56.5%	N/A
18	17%	56.1%	N/A
Final	N/A	N/A	58.1%

D) Analysis of monomer consumption versus time for a polymer containing 60 mol.% DMAEMA and 40 mol.% TEGMA was conducted by ^{1}H-NMR using DMF as reaction solvent and DMSO-d6 as a locking solvent for NMR. The indicated regions of interest (ROI) were integrated to determine the conversion of monomer at each time point. The molar ratio of DMAEMA and TEGMA in solution is stable throughout the reaction indicating that the monomers are incorporated into the polymer with similar kinetics. It should be noted that that, because of poor initiation of the DMAEMA-TEGMA co-polymerization resulting in lower monomer conversions, the first two kinetic analyses for this system were conducted at 2 and 4 h rather than 1 and 3 h.

Figure S2: Analysis of Monomer Incorporation Over Time for Polymerizations. Monomer consumption in each co-polymer system employed in this work was assessed by 400 MHz ^{1}H-NMR spectroscopy. For each combination, monomers were incorporated into the polymer backbone at a similar rate, which was important to prevent self-assembly behavior in solution.

Polymer Name	Mon1	Mon2	M_n (theory)	%Mon1 (theory)	% Mon1 (^{1}H-NMR)	M_n_Boc (SEC)	M_n (SEC)	*Đ* (SEC)
7.5k $AEMA_{50}$-s-BMA_{50}	AEMA	BMA	7,500	50%	52%	6,600	5,000	1.22
7.5k $AEMA_{60}$-s-BMA_{40}	AEMA	BMA	7,500	60%	62%	8,900	6,800	1.19
7.5k $AEMA_{70}$-s-BMA_{30}	AEMA	BMA	7,500	70%	76%	10,700	7,500	1.22
7.5k $AEMA_{80}$-s-BMA_{20}	AEMA	BMA	7,500	80%	82%	8,800	5,900	1.23
7.5k $AEMA_{90}$-s-BMA_{10}	AEMA	BMA	7,500	90%	91%	14,200	8,800	1.14
7.5k AEMA	AEMA	[redacted]	7,500	100%	100%	10,900	6,300	1.22
7.5k $AEMA_{90}$-s-$TEGMA_{10}$	AEMA	TEGMA	7,500	90%	91%	12,000	7,400	1.18
7.5k $AEMA_{80}$-s-$TEGMA_{20}$	AEMA	TEGMA	7,500	80%	79%	14,400	9,600	1.16
7.5k $AEMA_{70}$-s-$TEGMA_{30}$	AEMA	TEGMA	7,500	70%	76%	9,500	6,700	1.26
7.5k $AEMA_{60}$-s-$TEGMA_{40}$	AEMA	TEGMA	7,500	60%	64%	12,000	8,800	1.19
7.5k $AEMA_{50}$-s-$TEGMA_{50}$	AEMA	TEGMA	7,500	50%	43%	12,700	10,100	1.30
15k $AEMA_{50}$-s-BMA_{50}	AEMA	BMA	15,000	50%	54%	16,200	12,700	1.17
15k $AEMA_{60}$-s-BMA_{40}	AEMA	BMA	15,000	60%	63%	18,200	12,700	1.17
15k $AEMA_{70}$-s-BMA_{30}	AEMA	BMA	15,000	70%	68%	26,800	18,800	1.17
15k $AEMA_{80}$-s-BMA_{20}	AEMA	BMA	15,000	80%	81%	22,400	14,700	1.17
15k $AEMA_{90}$-s-BMA_{10}	AEMA	BMA	15,000	90%	91%	25,700	15,700	1.14
15k AEMA	AEMA	[redacted]	15,000	100%	100%	25,700	14,600	1.25
15k $AEMA_{90}$-s-$TEGMA_{10}$	AEMA	TEGMA	15,000	90%	89%	24,600	15,000	1.18
15k $AEMA_{80}$-s-$TEGMA_{20}$	AEMA	TEGMA	15,000	80%	79%	26,700	17,600	1.18
15k $AEMA_{70}$-s-$TEGMA_{30}$	AEMA	TEGMA	15,000	70%	75%	19,000	13,300	1.21
15k $AEMA_{60}$-s-$TEGMA_{40}$	AEMA	TEGMA	15,000	60%	63%	22,700	16,600	1.17
15k $AEMA_{50}$-s-$TEGMA_{50}$	AEMA	TEGMA	15,000	50%	45%	24,900	19,700	1.25
30k $AEMA_{50}$-s-BMA_{50}	AEMA	BMA	30,000	50%	44%	40,400	31,800	1.20
30k $AEMA_{60}$-s-BMA_{40}	AEMA	BMA	30,000	60%	62%	31,300	23,200	1.19
30k $AEMA_{70}$-s-BMA_{30}	AEMA	BMA	30,000	70%	65%	40,000	27,900	1.36
30k $AEMA_{80}$-s-BMA_{20}	AEMA	BMA	30,000	80%	83%	38,400	25,100	1.20
30k $AEMA_{90}$-s-BMA_{10}	AEMA	BMA	30,000	90%	92%	56,900	34,700	1.22
30k AEMA	AEMA	[redacted]	30,000	100%	100%	44,100	25,000	1.16
30k $AEMA_{90}$-s-$TEGMA_{10}$	AEMA	TEGMA	30,000	90%	91%	56,400	34,400	1.26
30k $AEMA_{80}$-s-$TEGMA_{20}$	AEMA	TEGMA	30,000	80%	82%	36,300	23,700	1.25
30k $AEMA_{70}$-s-$TEGMA_{30}$	AEMA	TEGMA	30,000	70%	74%	48,300	33,700	1.22
30k $AEMA_{60}$-s-$TEGMA_{40}$	AEMA	TEGMA	30,000	60%	66%	30,900	22,300	1.24
30k $AEMA_{50}$-s-$TEGMA_{50}$	AEMA	TEGMA	30,000	50%	57%	41,200	32,400	1.22
45k $AEMA_{50}$-s-BMA_{50}	AEMA	BMA	45,000	50%	42%	53,700	42,200	1.28
[redacted]								
45k $AEMA_{70}$-s-BMA_{30}	AEMA	BMA	45,000	70%	70%	41,800	29,200	1.20
45k $AEMA_{80}$-s-BMA_{20}	AEMA	BMA	45,000	80%	80%	63,400	41,400	1.40
45k $AEMA_{90}$-s-BMA_{10}	AEMA	BMA	45,000	90%	92%	68,800	41,900	1.39
45k AEMA	AEMA	[redacted]	45,000	100%	100%	58,900	33,300	1.18
45k $AEMA_{90}$-s-$TEGMA_{10}$	AEMA	TEGMA	45,000	90%	91%	76,800	46,800	1.32
45k $AEMA_{80}$-s-$TEGMA_{20}$	AEMA	TEGMA	45,000	80%	81%	71,200	46,500	1.29
45k $AEMA_{70}$-s-$TEGMA_{30}$	AEMA	TEGMA	45,000	70%	72%	55,800	38,900	1.47
45k $AEMA_{60}$-s-$TEGMA_{40}$	AEMA	TEGMA	45,000	60%	57%	52,800	41,500	1.34
45k $AEMA_{50}$-s-$TEGMA_{50}$	AEMA	TEGMA	45,000	50%	53%	72,600	53,800	1.40
[redacted]								
60k $AEMA_{60}$-s-BMA_{40}	AEMA	BMA	60,000	60%	60%	66,900	49,500	1.25
60k $AEMA_{70}$-s-BMA_{30}	AEMA	BMA	60,000	70%	67%	76,800	53,500	1.35
60k $AEMA_{80}$-s-BMA_{20}	AEMA	BMA	60,000	80%	82%	81,100	52,900	1.26
60k $AEMA_{90}$-s-BMA_{10}	AEMA	BMA	60,000	90%	88%	76,100	46,300	1.58
60k AEMA	AEMA	[redacted]	60,000	100%	100%	90,100	50,900	1.23

60k AEMA$_{90}$-s-TEGMA$_{10}$	AEMA	TEGMA	60,000	90%	90%	106,200	64,600	1.27
60k AEMA$_{80}$-s-TEGMA$_{20}$	AEMA	TEGMA	60,000	80%	82%	73,000	47,600	1.37
60k AEMA$_{70}$-s-TEGMA$_{30}$	AEMA	TEGMA	60,000	70%	72%	85,000	59,100	1.31
60k AEMA$_{60}$-s-TEGMA$_{40}$	AEMA	TEGMA	60,000	60%	66%	66,700	49,300	1.26
60k AEMA$_{50}$-s-TEGMA$_{50}$	AEMA	TEGMA	60,000	50%	57%	68,800	54,000	1.31
7.5k DMAEMA$_{50}$-s-BMA$_{50}$	DMAEMA	BMA	7,500	50%	45%		6,000	1.37
7.5k DMAEMA$_{60}$-s-BMA$_{40}$	DMAEMA	BMA	7,500	60%	59%		7,000	1.35
7.5k DMAEMA$_{70}$-s-BMA$_{30}$	DMAEMA	BMA	7,500	70%	69%		5,900	1.37
7.5k DMAEMA$_{80}$-s-BMA$_{20}$	DMAEMA	BMA	7,500	80%	76%		5,600	1.32
7.5k DMAEMA$_{90}$-s-BMA$_{10}$	DMAEMA	BMA	7,500	90%	91%		5,400	1.37
7.5k DMAEMA	DMAEMA		7,500	100%	100%		8,700	1.33
7.5k DMAEMA$_{90}$-s-TEGMA$_{10}$	DMAEMA	TEGMA	7,500	90%	86%		5,900	1.39
7.5k DMAEMA$_{80}$-s-TEGMA$_{20}$	DMAEMA	TEGMA	7,500	80%	81%		6,100	1.45
7.5k DMAEMA$_{70}$-s-TEGMA$_{30}$	DMAEMA	TEGMA	7,500	70%	66%		5,700	1.39
7.5k DMAEMA$_{60}$-s-TEGMA$_{40}$	DMAEMA	TEGMA	7,500	60%	60%		7,800	1.39
7.5k DMAEMA$_{50}$-s-TEGMA$_{50}$	DMAEMA	TEGMA	7,500	50%	51%		5,600	1.37
15k DMAEMA$_{50}$-s-BMA$_{50}$	DMAEMA	BMA	15,000	50%	52%		12,300	1.28
15k DMAEMA$_{60}$-s-BMA$_{40}$	DMAEMA	BMA	15,000	60%	65%		11,500	1.35
15k DMAEMA$_{70}$-s-BMA$_{30}$	DMAEMA	BMA	15,000	70%	70%		11,500	1.39
15k DMAEMA$_{80}$-s-BMA$_{20}$	DMAEMA	BMA	15,000	80%	79%		13,200	1.27
15k DMAEMA$_{90}$-s-BMA$_{10}$	DMAEMA	BMA	15,000	90%	93%		13,000	1.32
15k DMAEMA	DMAEMA		15,000	100%	100%		10,800	1.32
15k DMAEMA$_{90}$-s-TEGMA$_{10}$	DMAEMA	TEGMA	15,000	90%	90%		9,700	1.26
15k DMAEMA$_{80}$-s-TEGMA$_{20}$	DMAEMA	TEGMA	15,000	80%	79%		9,300	1.20
15k DMAEMA$_{70}$-s-TEGMA$_{30}$	DMAEMA	TEGMA	15,000	70%	64%		11,500	1.37
15k DMAEMA$_{60}$-s-TEGMA$_{40}$	DMAEMA	TEGMA	15,000	60%	55%		18,700	1.56
15k DMAEMA$_{50}$-s-TEGMA$_{50}$	DMAEMA	TEGMA	15,000	50%	50%		11,400	1.36
30k DMAEMA$_{50}$-s-BMA$_{50}$	DMAEMA	BMA	30,000	50%	43%		30,900	1.35
30k DMAEMA$_{60}$-s-BMA$_{40}$	DMAEMA	BMA	30,000	60%	62%		23,600	1.31
30k DMAEMA$_{70}$-s-BMA$_{30}$	DMAEMA	BMA	30,000	70%	73%		22,200	1.53
30k DMAEMA$_{80}$-s-BMA$_{20}$	DMAEMA	BMA	30,000	80%	83%		24,000	1.37
30k DMAEMA$_{90}$-s-BMA$_{10}$	DMAEMA	BMA	30,000	90%	86%		30,700	1.49
30k DMAEMA	DMAEMA		30,000	100%	100%		36,900	1.35
30k DMAEMA$_{90}$-s-TEGMA$_{10}$	DMAEMA	TEGMA	30,000	90%	85%		30,800	1.53
30k DMAEMA$_{80}$-s-TEGMA$_{20}$	DMAEMA	TEGMA	30,000	80%	74%		21,100	1.53
30k DMAEMA$_{70}$-s-TEGMA$_{30}$	DMAEMA	TEGMA	30,000	70%	68%		34,400	1.43
30k DMAEMA$_{60}$-s-TEGMA$_{40}$	DMAEMA	TEGMA	30,000	60%	56%		33,900	1.51
30k DMAEMA$_{50}$-s-TEGMA$_{50}$	DMAEMA	TEGMA	30,000	50%	50%		32,000	1.47
45k DMAEMA$_{50}$-s-BMA$_{50}$	DMAEMA	BMA	45,000	50%	48%		39,600	1.46
45k DMAEMA$_{60}$-s-BMA$_{40}$	DMAEMA	BMA	45,000	60%	61%		34,400	1.32
45k DMAEMA$_{70}$-s-BMA$_{30}$	DMAEMA	BMA	45,000	70%	67%		38,500	1.58
45k DMAEMA$_{80}$-s-BMA$_{20}$	DMAEMA	BMA	45,000	80%	82%		32,000	1.47
45k DMAEMA$_{90}$-s-BMA$_{10}$	DMAEMA	BMA	45,000	90%	91%		38,200	1.66
45k DMAEMA	DMAEMA		45,000	100%	100%		40,000	1.51
45k DMAEMA$_{90}$-s-TEGMA$_{10}$	DMAEMA	TEGMA	45,000	90%	83%		42,500	1.57
45k DMAEMA$_{80}$-s-TEGMA$_{20}$	DMAEMA	TEGMA	45,000	80%	76%		35,100	1.43
45k DMAEMA$_{70}$-s-TEGMA$_{30}$	DMAEMA	TEGMA	45,000	70%	68%		42,800	1.66
45k DMAEMA$_{60}$-s-TEGMA$_{40}$	DMAEMA	TEGMA	45,000	60%	54%		46,100	1.70
45k DMAEMA$_{50}$-s-TEGMA$_{50}$	DMAEMA	TEGMA	45,000	50%	51%		42,900	1.65
60k DMAEMA$_{50}$-s-BMA$_{50}$	DMAEMA	BMA	60,000	50%	54%		46,400	1.61
60k DMAEMA$_{60}$-s-BMA$_{40}$	DMAEMA	BMA	60,000	60%	67%		48,700	1.44
60k DMAEMA$_{70}$-s-BMA$_{30}$	DMAEMA	BMA	60,000	70%	67%		47,900	1.65

60k $DMAEMA_{80}$-s-BMA_{20}	DMAEMA	BMA	60,000	80%	82%	[redacted]	46,900	1.73
[redacted]								
60k DMAEMA	DMAEMA	[redacted]	60,000	100%	100%	[redacted]	45,400	1.36
60k $DMAEMA_{90}$-s-$TEGMA_{10}$	DMAEMA	TEGMA	60,000	90%	88%	[redacted]	48,200	1.85
60k $DMAEMA_{80}$-s-$TEGMA_{20}$	DMAEMA	TEGMA	60,000	80%	79%	[redacted]	50,800	1.24
60k $DMAEMA_{70}$-s-$TEGMA_{30}$	DMAEMA	TEGMA	60,000	70%	67%	[redacted]	48,300	1.88
60k $DMAEMA_{60}$-s-$TEGMA_{40}$	DMAEMA	TEGMA	60,000	60%	59%	[redacted]	47,200	1.57
60k $DMAEMA_{50}$-s-$TEGMA_{50}$	DMAEMA	TEGMA	60,000	50%	48%	[redacted]	78,800	1.58

Figure S3: Polymer Characterization Data Table. Full ^{1}H-NMR spectroscopy and SEC characterization data of polymer library. 400 MHz ^{1}H-NMR spectra and SEC analyses in DMF are provided in the *^{1}H-NMR and SEC of Polymer Library* section of the supplementary information.

A) 30 kg/mol poly($AEMA_{60}$-*s*-BMA_{40})

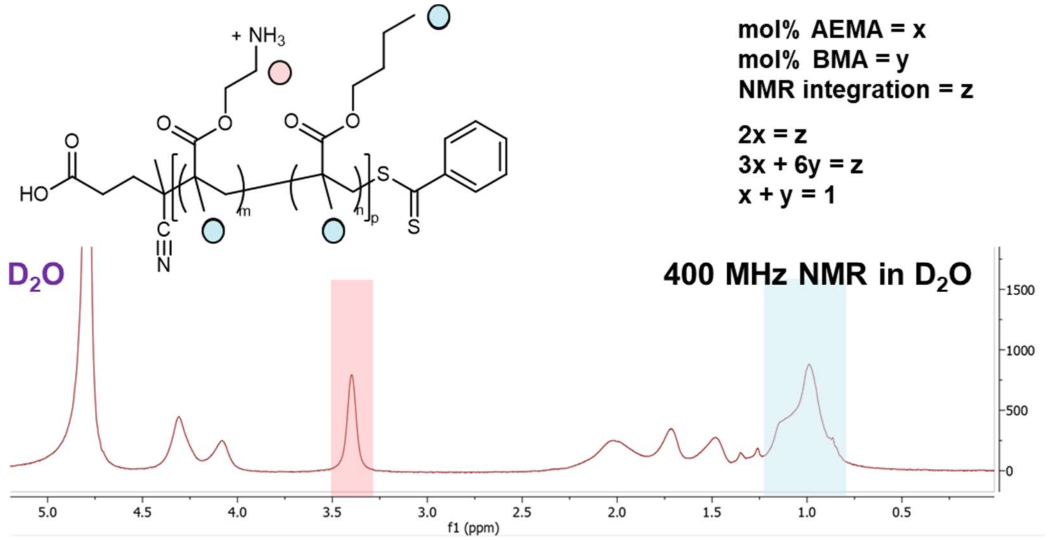

B) 30 kg/mol poly($AEMA_{60}$-*s*-$TEGMA_{40}$)

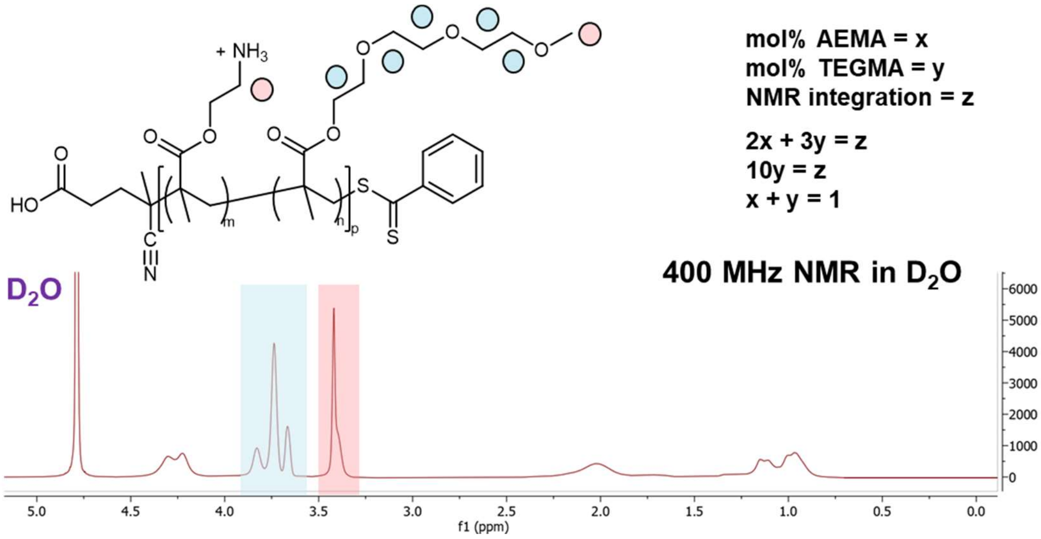

C) 30 kg/mol poly($DMAEMA_{60}$-*s*-BMA_{40})

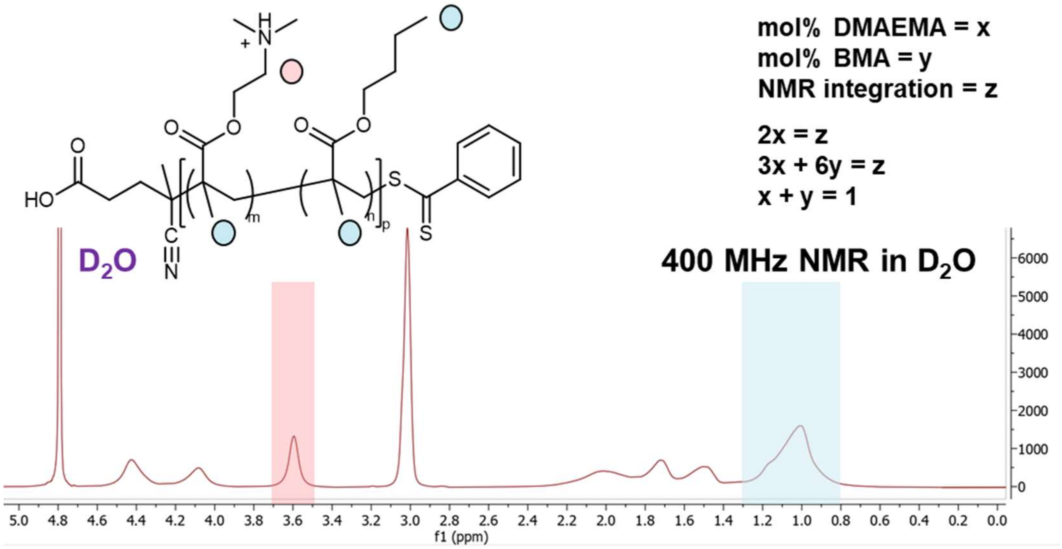

D) 30 kg/mol poly($DMAEMA_{60}$-*s*-$TEGMA_{40}$)

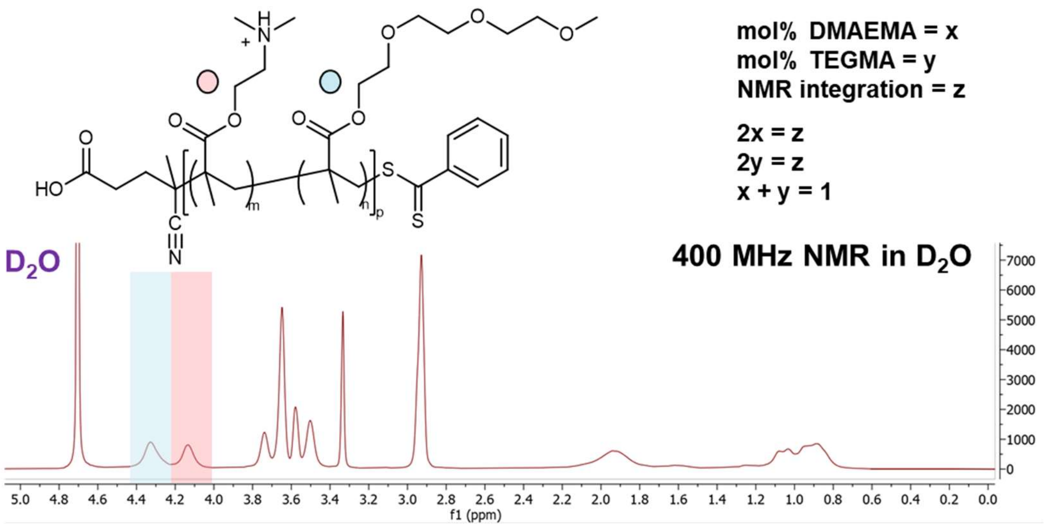

Figure S4: NMR Diagnostic Peaks for Polymer Analyses. Schematic analysis of 400 MHz ^{1}H-NMR spectra used to determine polymer composition for each of the four combinations of "core" (AEMA or DMAEMA) and "dopant" (BMA or TEGMA) monomers after deprotection and dialysis. NMR spectra correspond to the 60:40 core:dopant polymers prepared for monomer consumption analysis in **Figure S2**. Integration of the red ROI is defined as "x", and integration of the blue ROI is defined as "y". Based on the relative integrations, a system of equations was defined to determine the relative ratio of core and dopant monomer incorporated into the polymer backbone.

Polymer ID	M_n (SEC)	D (DOSY-NMR)
15k $AEMA_{70}$-BMA_{30}	18,800 g/mol	2.2 x 10^{-11} m^2/s
15k $AEMA_{70}$-$TEGMA_{30}$	13,300 g/mol	1.8 x 10^{-12} m^2/s
15k $DMAEMA_{70}$-BMA_{30}	11,500 g/mol	3.4 x 10^{-12} m^2/s
15k $DMAEMA_{70}$-$TEGMA_{30}$	11,500 g/mol	2.9 x 10^{-12} m^2/s
60k $AEMA_{70}$-BMA_{30}	53,500 g/mol	7.0 x 10^{-12} m^2/s
60k $AEMA_{70}$-$TEGMA_{30}$	59,100 g/mol	7.1 x 10^{-12} m^2/s
60k $DMAEMA_{70}$-BMA_{30}	47,900 g/mol	1.0 x 10^{-12} m^2/s
60k $DMAEMA_{70}$-$TEGMA_{30}$	48,300 g/mol	1.2 x 10^{-12} m^2/s

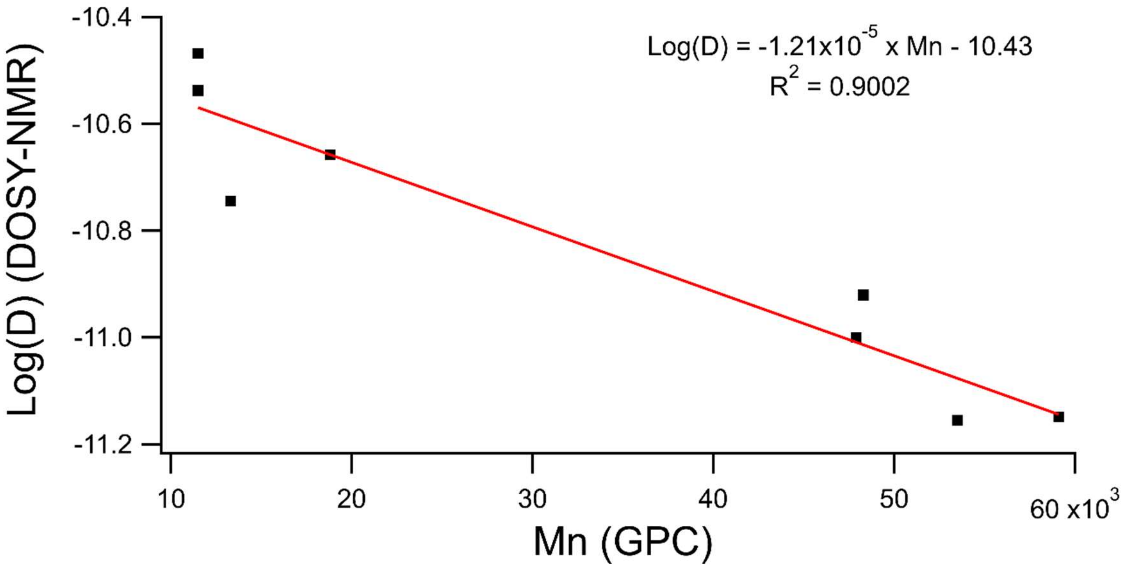

Figure S5: DOSY-NMR of Selected Polymers and Comparison to GPC. Diffusion constants determined by DOSY-NMR of polymers in D_2O after deprotection with TFA were used to develop a correlation between diffusion constants (D) determined by DOSY-NMR and M_n determined by SEC prior to deprotection. For a monodisperse polymer in dilute solution, M_n and Log(D) have a linear relationship,[S3] thereby providing evidence that polymer molecular weight was maintained during the deprotection process.

A

Polymer Composition	Number Avg. Hydrodynamic Radius at 25 °C (R_h, DLS)	Number Avg. Hydrodynamic Radius at 37 °C (R_h, DLS)
15k $AEMA_{70}$-BMA_{30}	11.32 ± 1.84 nm	84.47 ± 1.50 nm
15k $AEMA_{70}$-$TEGMA_{30}$	3.90 ± 0.06 nm	28.00 ± 0.81 nm
15k $DMAEMA_{70}$-BMA_{30}	9.92 ± 3.46 nm	203.3 ± 13.7 nm (&)
15k $DMAEMA_{70}$-$TEGMA_{30}$	4.28 ± 0.06 nm	12.24 ± 0.43 nm
60k $AEMA_{70}$-BMA_{30}	10.37 ± 0.16 nm	81.14 ± 2.17 nm
60k $AEMA_{70}$-$TEGMA_{30}$	11.17 ± 0.97 nm	16.33 ± 1.99 nm
60k $DMAEMA_{70}$-BMA_{30}	6.89 ± 0.51 nm	64.52 ± 5.84 nm (&)
60k $DMAEMA_{70}$-$TEGMA_{30}$	14.03 ± 0.49 nm	18.34 ± 0.48 nm

B

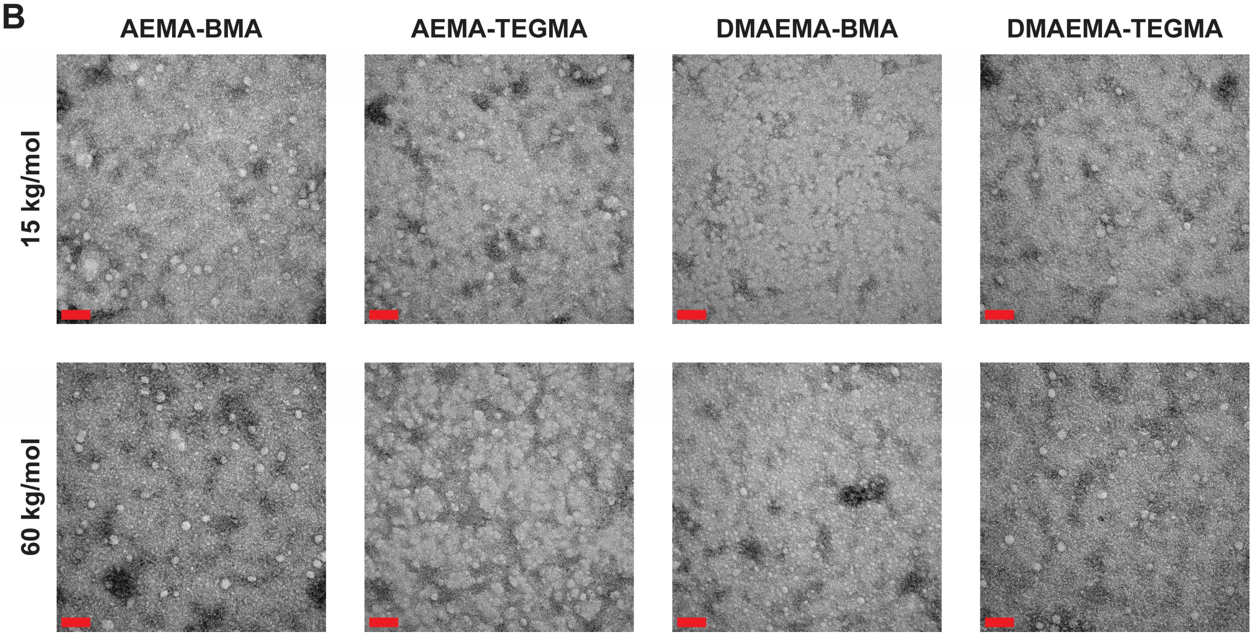

Figure S6: DLS and TEM of Selected Polymers for Aggregation Analysis. A) Aggregation of polymers in cell culture medium (1 mg/mL polymer in phenol red-free RPMI + 10% FBS) was assessed using dynamic light scattering at ambient (25 °C) and biological (37 °C) temperature. The hydrodynamic radii (R_h) observed suggest that the polymers used in this study do not self-assemble in cell culture. For (&) denoted samples, the R_h observed increased over time due to aggregation upon heating. B) Polymers were dissolved at 1 mg/mL in cell culture medium, plated on holey carbon grids, stained with uranium acetate, and imaged by TEM (Red scale bar = 100 nm). No evidence of ordered polymer self-assembly is observed, though spherical protein aggregates of ~20 nm are found in most samples.

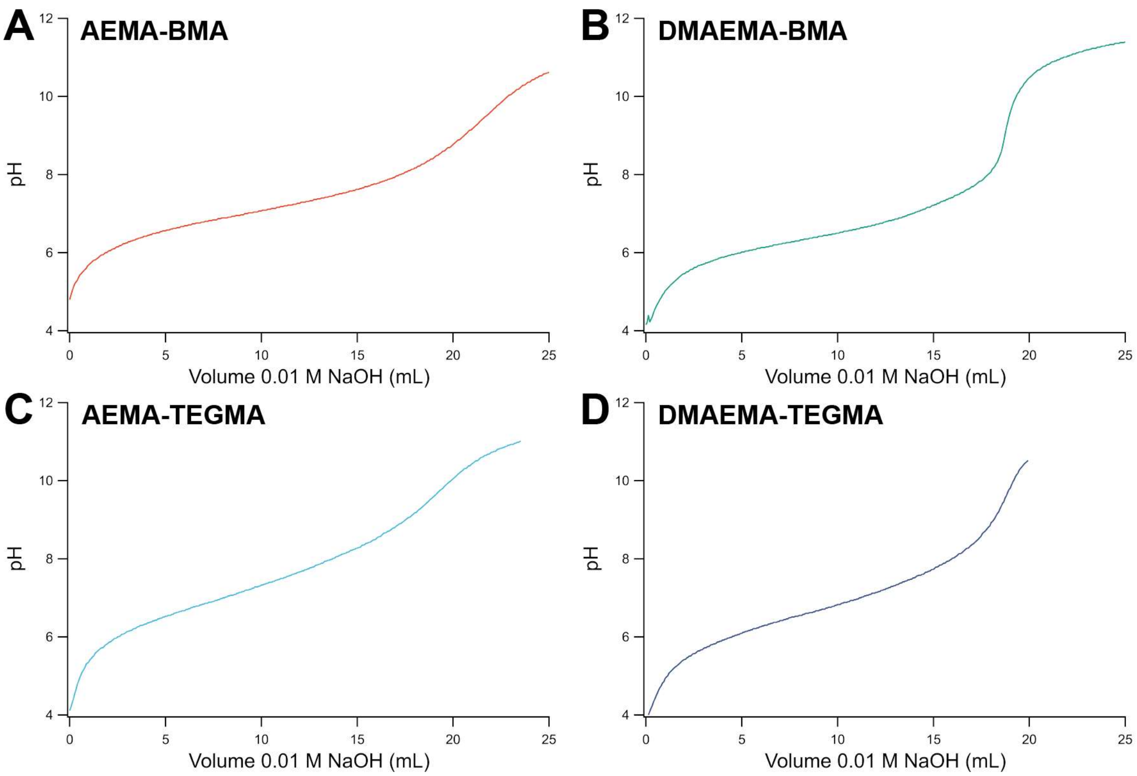

E) Calculated pK_a values for each co-polymer

Co-polymer	pK_a
15k AEMA-BMA	7.18
15k DMAEMA-BMA	6.44
15k AEMA-TEGMA	7.25
15k DMAEMA-TEGMA	6.71

Figure S7: Titration and pK_a Values of Selected Polymers. (A-D) Polymers from each of the four monomer combinations (70:30 ratio, 15 kg/mol) were dissolved at 1 mg/mL in deionized water and adjusted to pH = 4.0. They were then titrated with 0.01 M NaOH to determine the approximate pK_a of each co-polymer. (E) The pK_a values determined via titration are shown in the accompanying table.

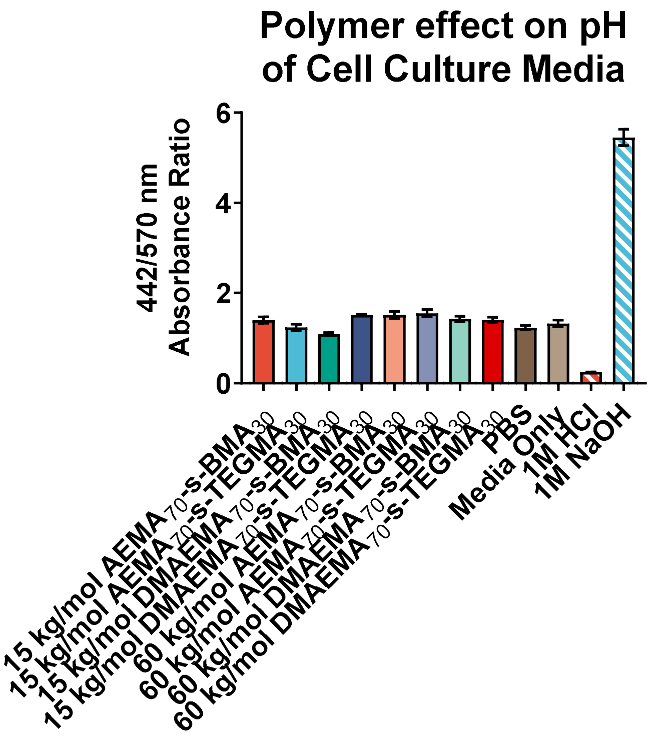

Figure S8: Effect of Polymers on pH of Cell Culture Media. To determine the effect of polymers on the pH of cell culture medium, polymers or controls were added to cell culture medium (100 μg/mL polymer in phenol red-containing RPMI + 10% FBS), and the emission spectrum of phenol red (a colorimetric pH indicator between 6.8 and 8.2) was analyzed by plate reader using a broad-spectrum scan (350-750 nm, 2 nm step size). The ratio of absorbance at 442 and 570 nm was used to determine the acidity of solution.

A) IL-1β Secretion

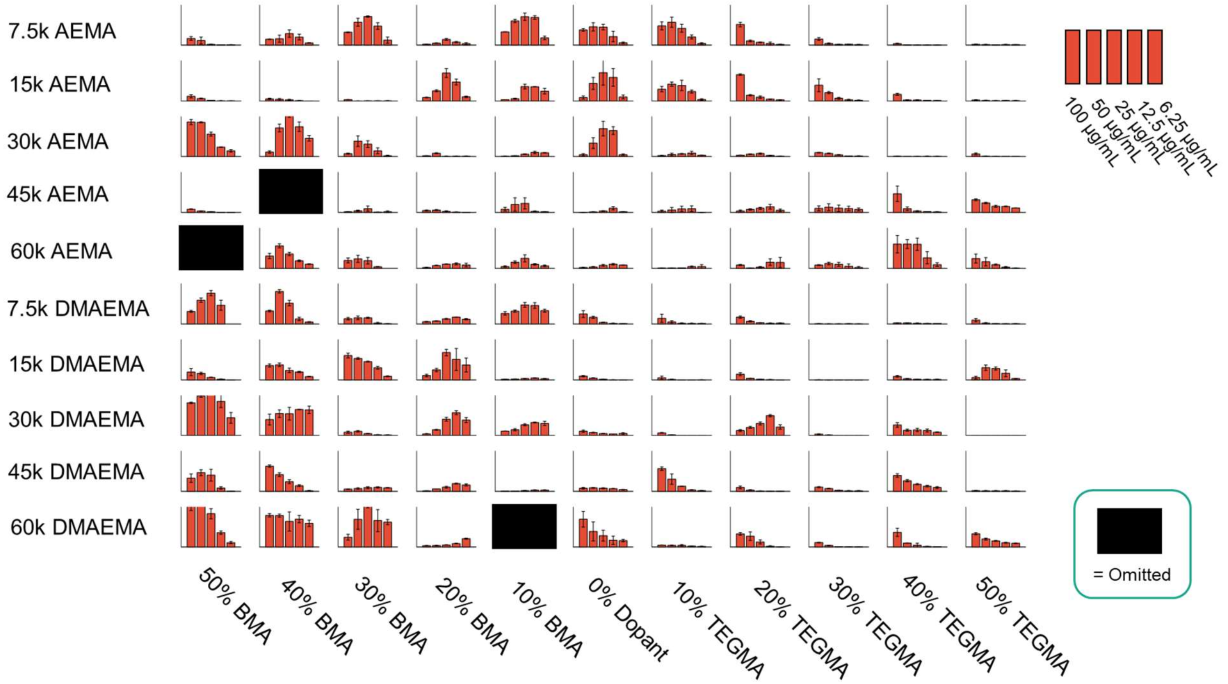

B) LDH Secretion

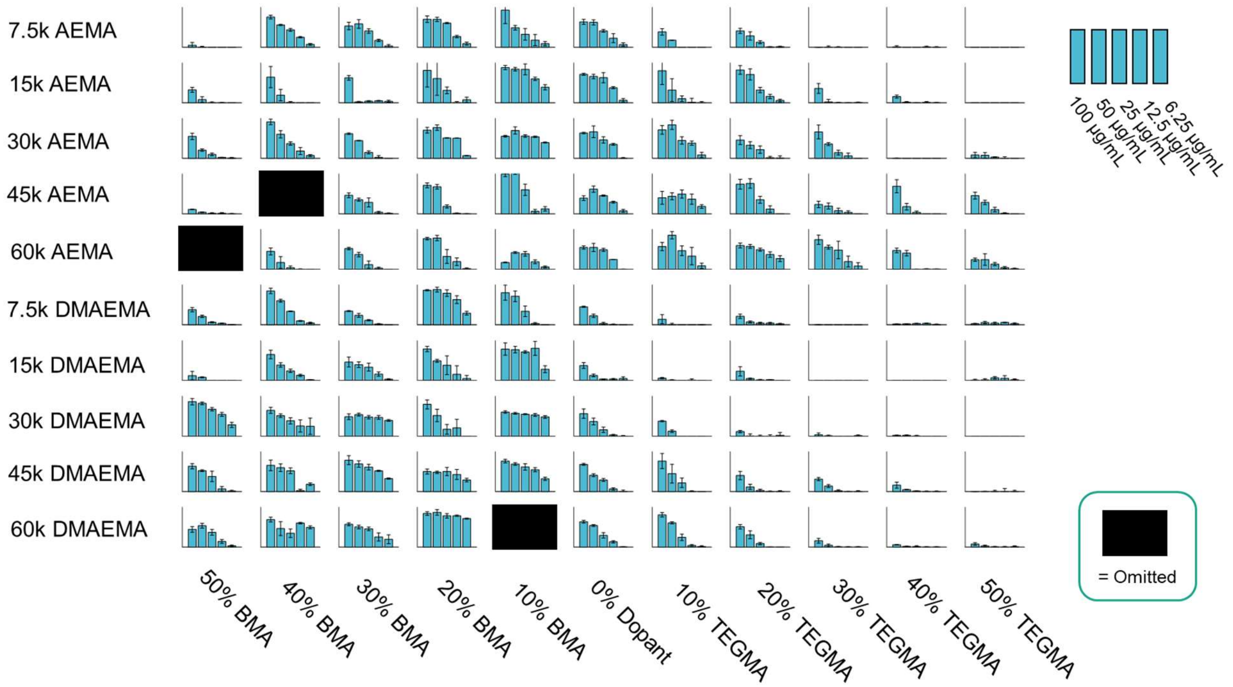

Figure S9: Full Results of In-Vitro Screening. High throughput screening was conducted for each of the 107 polymers five concentrations tested to determine (A) IL-1β and (B) LDH secretion.

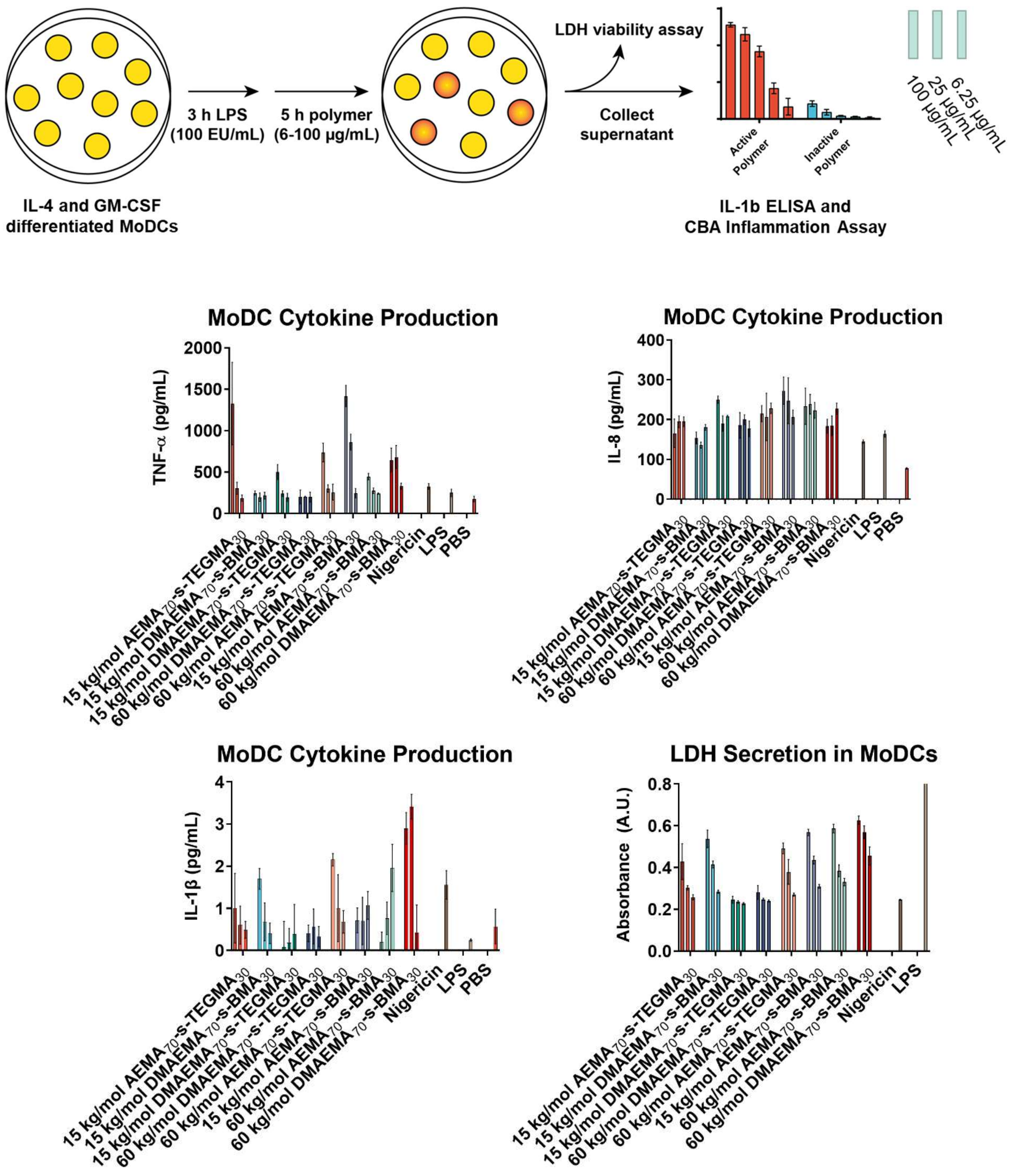

Figure S10: Polymer-Induced LDH and Cytokine Production in MoDCs. IL-4 and GM-CSF differentiated primary human monocytes (MoDCs) were primed with 100 EU/mL LPS and then treated with polymers. Cytokine production was then assayed using human CBA inflammation kit (TNF-α and IL-8) or ELISA (IL-1β), and LDH was assayed by CyQUANT LDH Cytotoxicity assay.

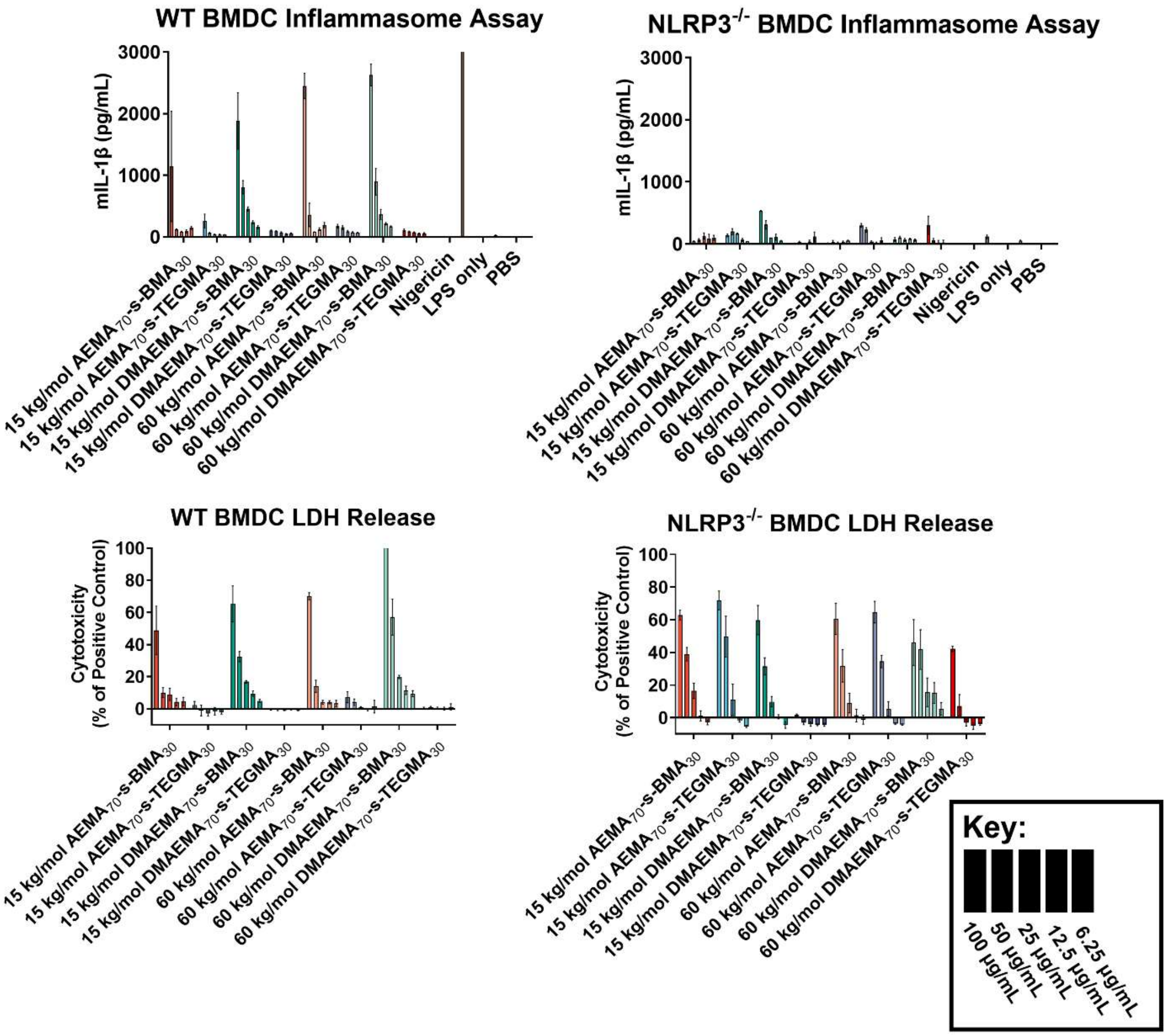

Figure S11: Polymer-Induced LDH and Cytokine Production in BMDCs. GM-CSF differentiated murine dendritic cells (BMDCs) from wild-type or NLRP3-KO C57Bl6/J mice were primed with 100 EU/mL LPS and then treated with polymers. IL-1β production was analyzed by ELISA, and LDH was assayed by CyQUANT LDH Cytotoxicity assay. The IL-1β production results shown are also plotted in **Figure 3B-C** in the manuscript.

A

Polymer (with Mn)	Vendor	Product Number	Reported Đ
60 kg/mol branched PEI	Sigma	181978	12.5
10 kg/mol branched PEI	Sigma	408727	2.5
1.8 kg/mol branched PEI	Sigma	408700	1.11

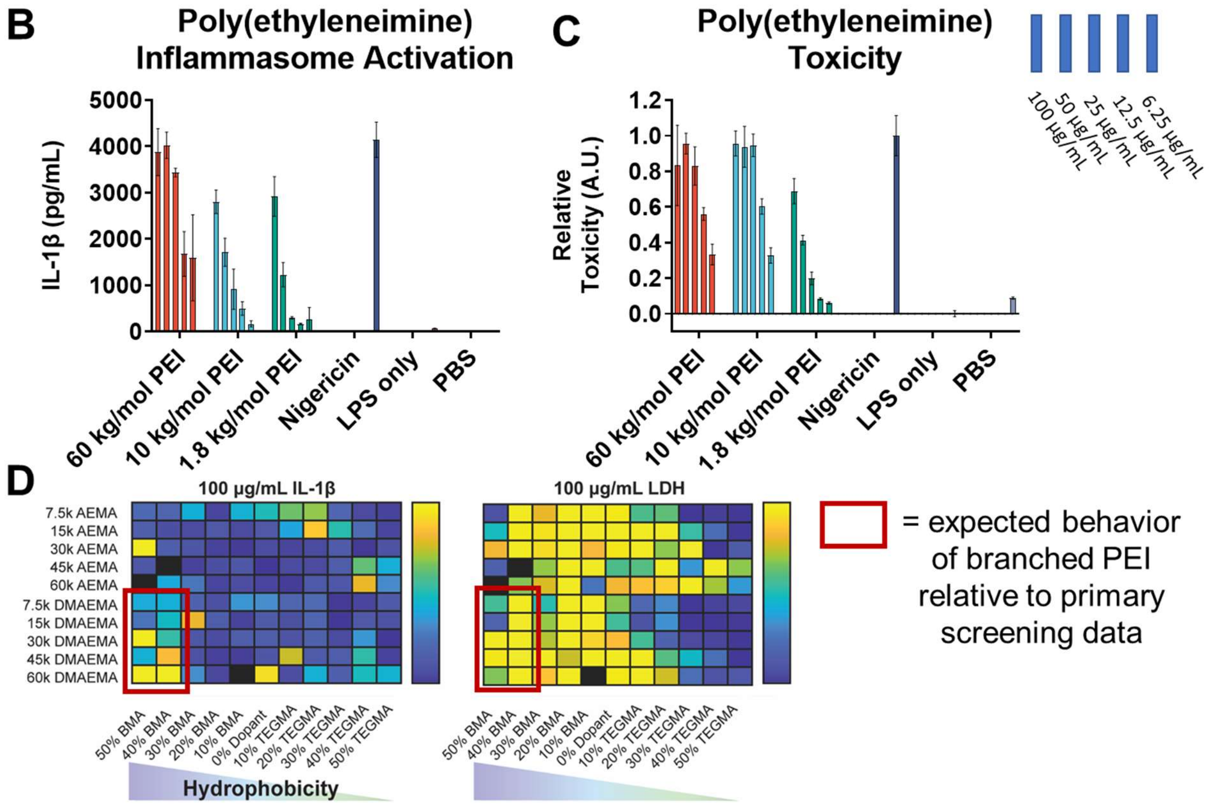

Figure S12: In-vitro Screening of Branched Poly(ethyleneimine). (A) Three branched poly(ethyleneimine) (PEI) compounds with different molecular weights were purchased with the reported characteristics. LPS-primed BMDCs were treated with the indicated polymers for 5 h, and (B) IL-1β and (C) LDH were assayed in the supernatant. (D) Based on the results of (B) and (C), branched PEI is expected to behave similarly to the polymers noted in red from the high throughput screen.

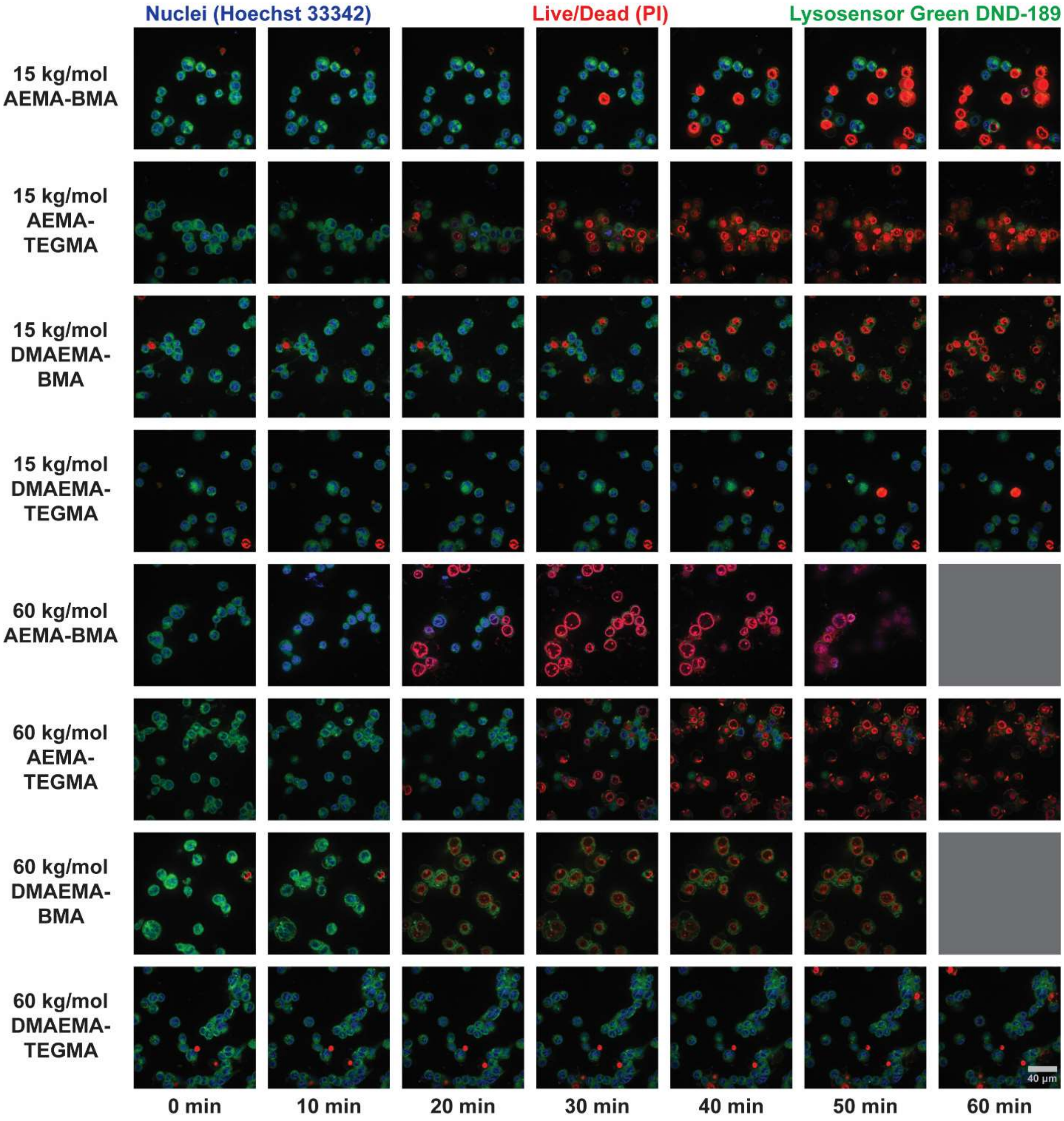

Figure S13: Raw Images of Lysosensor Green Assay in WT THP-1 Cells. THP-1 cells were stained with LysoSensor Green DND-189, Propidium Iodide, and Hoechst 33342 and then treated with 100 µg/mL of the indicated polymers. Cells were then imaged every 2 min for 1 h using confocal microscopy (40x oil lens) to evaluate lysosomal pH and cellular morphology. 10 min intervals of selected polymers are shown here, and full videos are available in *Supplementary Video 1*. Scale bar is representative of all images.

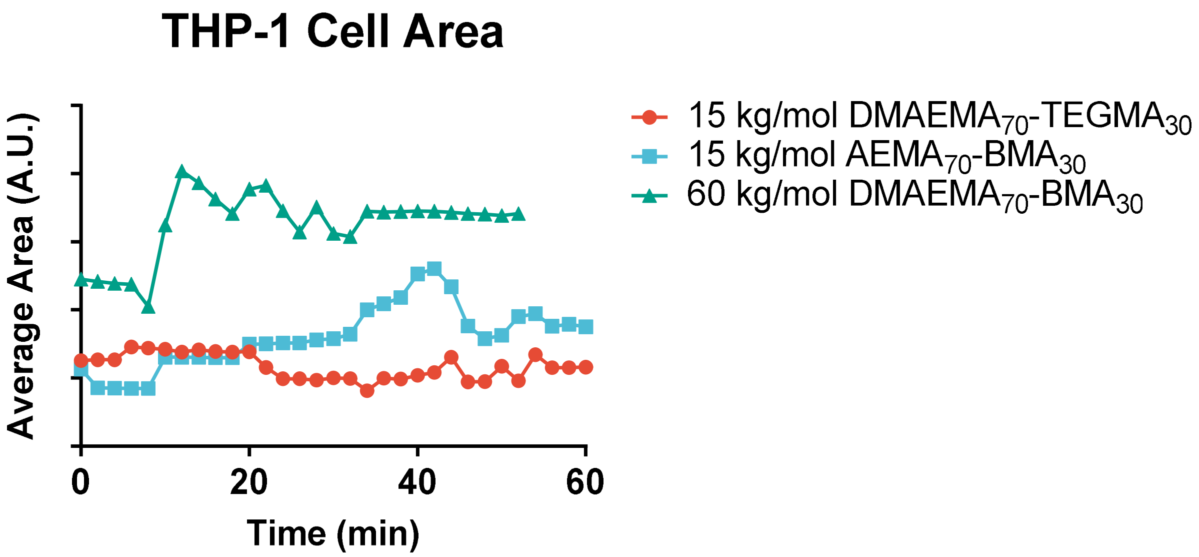

Figure S14: Cell Swelling of THP-1 Cells. Selected treatment groups from **Figure S13** were processed using CellProfiler to quantitatively determine swelling as a function of time.

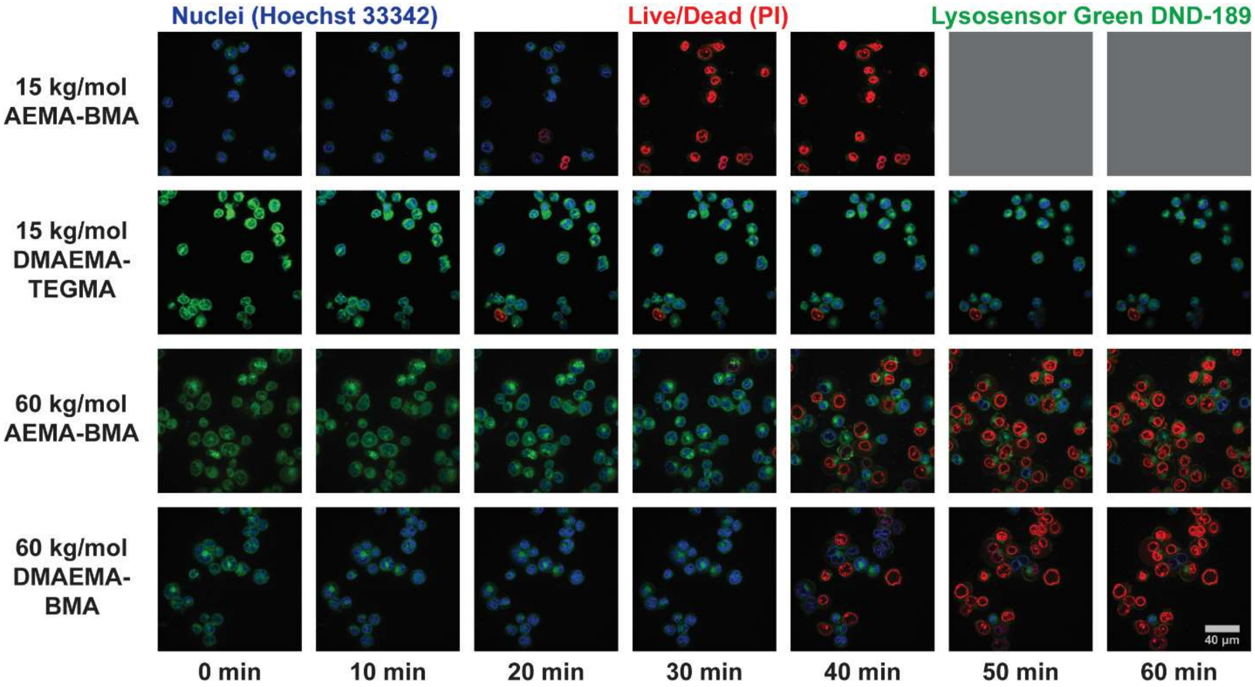

Figure S15: Raw Images of Lysosensor Green Assay in NLRP3-KO THP-1 Cells. NLRP3-KO THP-1 cells were stained with LysoSensor Green DND-189, Propidium Iodide, and Hoechst 33342 and then treated with 100 µg/mL of the indicated polymers. Cells were then imaged every 2 min for 1 h using confocal microscopy to evaluate lysosomal pH and cellular morphology. 10 min intervals of selected polymers are shown here, and full videos are available in *Supplementary Video 2*. Scale bar is representative of all images.

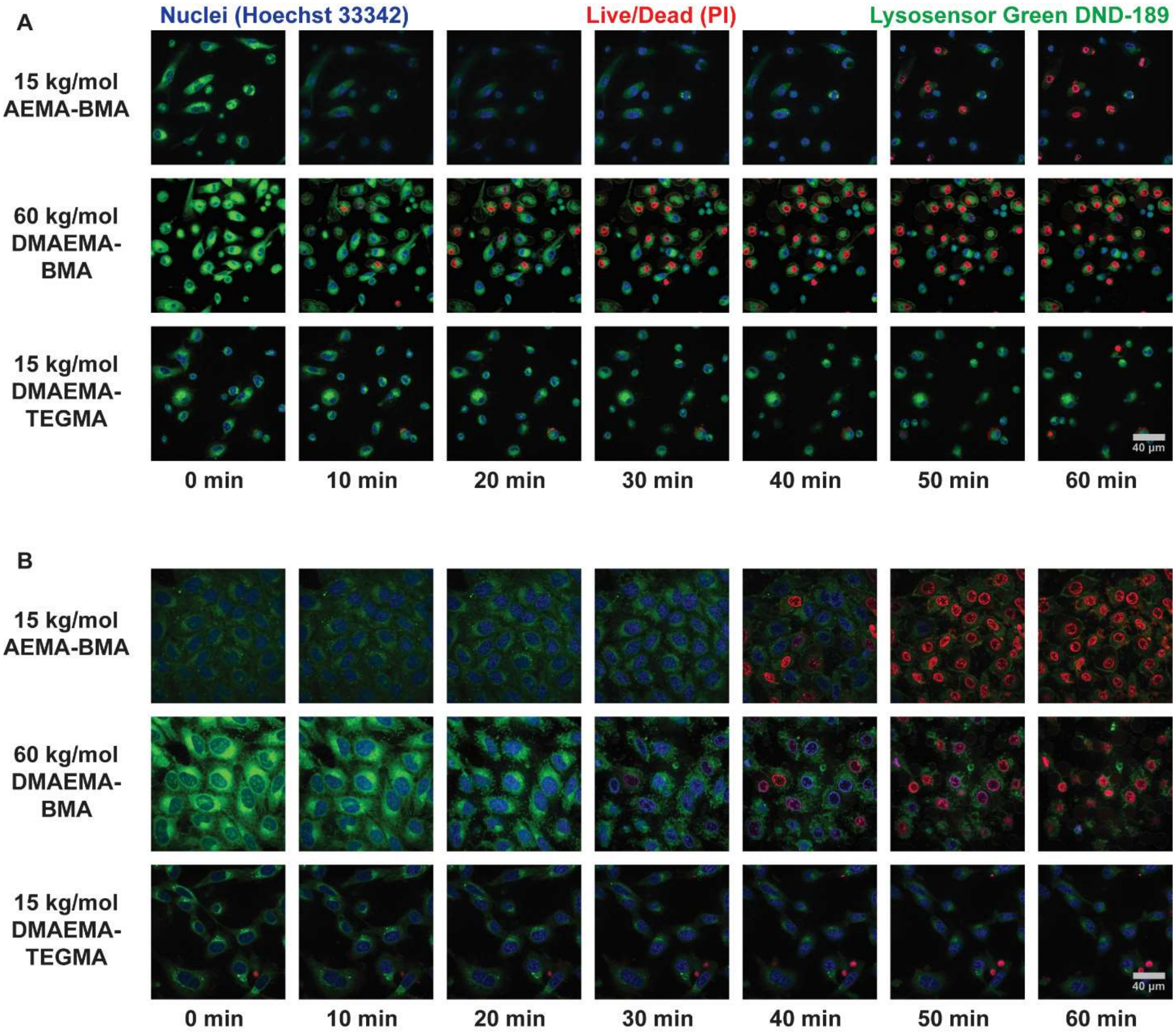
A
Nuclei (Hoechst 33342)
Live/Dead (PI)
Lysosensor Green DND-189
15 kg/mol AEMA-BMA
60 kg/mol DMAEMA-BMA
15 kg/mol DMAEMA-TEGMA
40 μm
0 min
10 min
20 min
30 min
40 min
50 min
60 min
B
15 kg/mol AEMA-BMA
60 kg/mol DMAEMA-BMA
15 kg/mol DMAEMA-TEGMA
40 μm
0 min
10 min
20 min
30 min
40 min
50 min
60 min

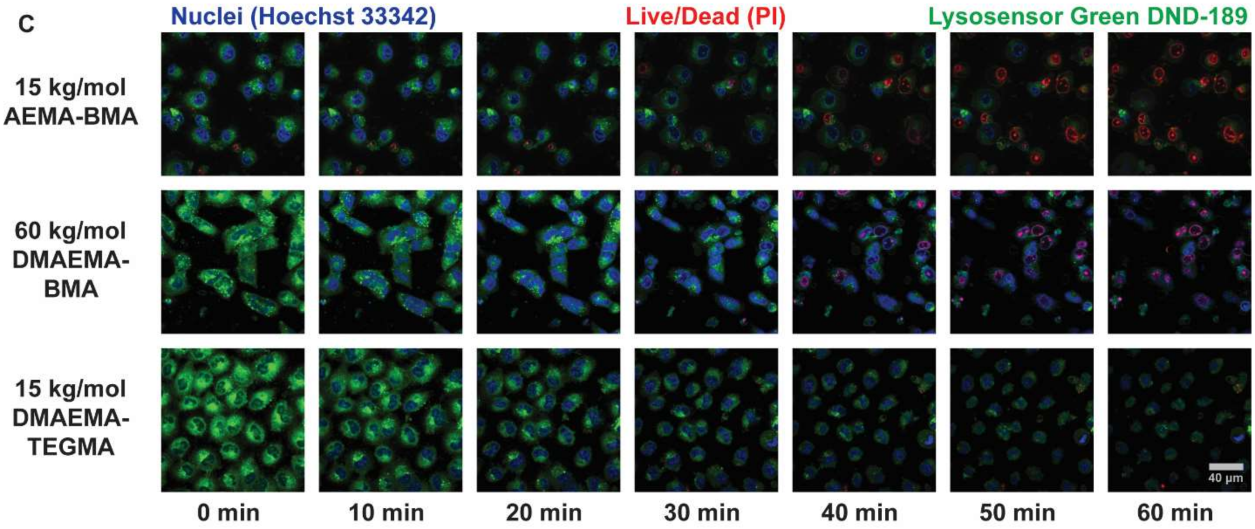

Figure S16: Raw Images of Lysosensor Green Assay in Other Cell Lines. (A) Bone marrow-derived dendritic cells (BMDCs), (B) HeLa cells, or (C) A549 cells were stained with LysoSensor Green DND-189, Propidium Iodide, and Hoechst 33342 and then treated with 100 µg/mL of the indicated polymers. Cells were then imaged every 2 min for 1 h using confocal microscopy to evaluate lysosomal pH and cellular morphology. 10 min intervals of selected polymers are shown here, and full videos are available in *Supplementary Video 3*. Scale bar is representative of all images.

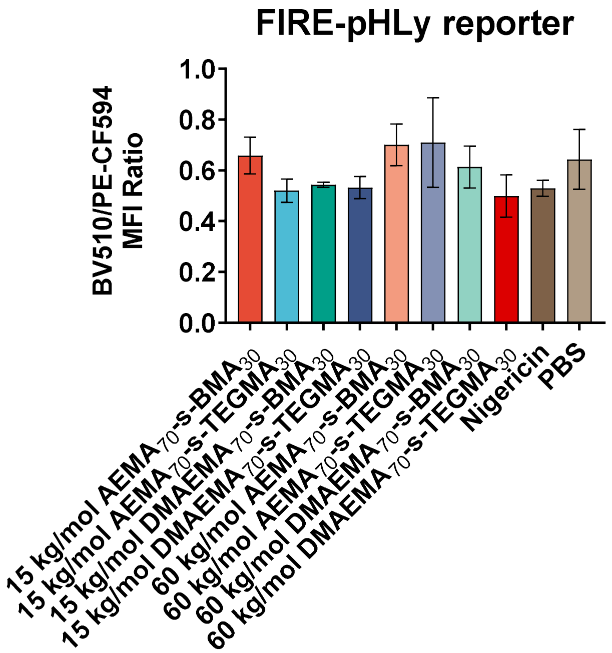

Figure S17: HEK FIRE-pHLy Lysosomal pH Assay. HEK FIRE-pHLy cells were treated with polymers at 25 µg/mL for 1 h, and the ratio of mTFP1 to mCherry fluorescence was analyzed by flow cytometry. mTFP1 is a pH sensitive lysosomal reporter where greater fluorescence indicates lysosomal acidification.

A) Synthetic Scheme

Synthesis of DMAEMA-containing polymers with 5 mol.% AEMA for fluorescent tagging

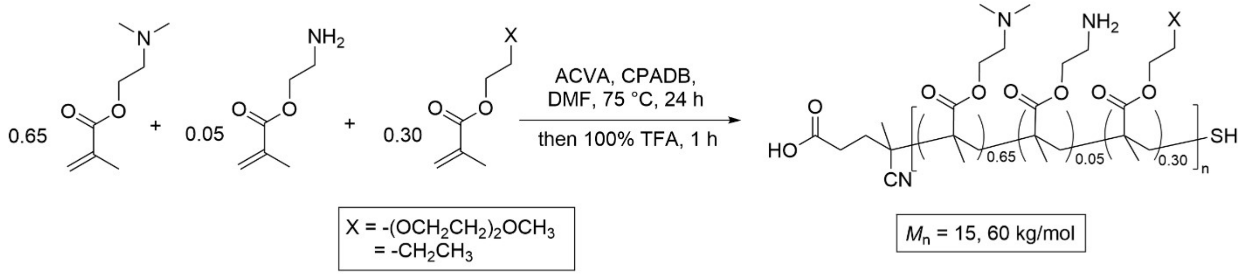

Fluorescent tagging of polymers with AF488-NHS Ester

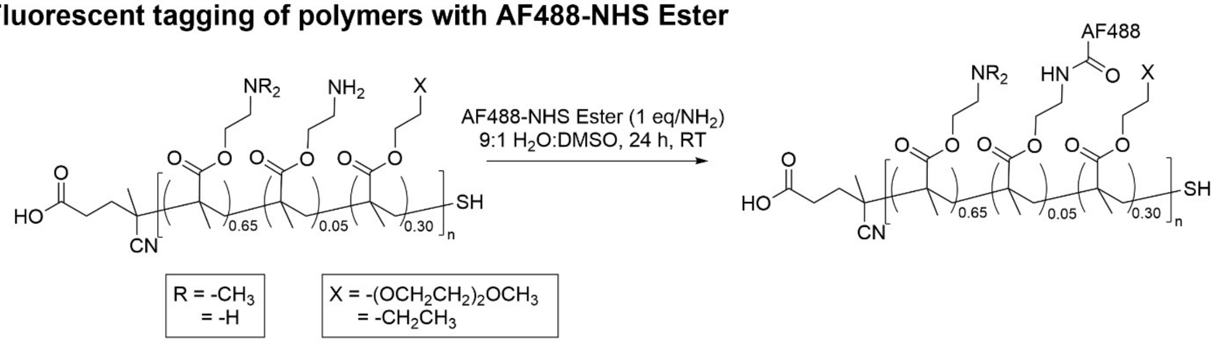

B) Characterization Table

Polymer Name	Mon1	Mon2	M_n (theory)	%Mon1 (theory)	% Mon1 (^{1}H-NMR)	M_n (SEC)	*Đ* (SEC)
15k $AEMA_{70}$-s-BMA_{30}	AEMA	BMA	15,000	70%	68%	18,800	1.17
15k $AEMA_{70}$-s-$TEGMA_{30}$	AEMA	TEGMA	15,000	70%	75%	13,300	1.21
60k $AEMA_{70}$-s-BMA_{30}	AEMA	BMA	60,000	70%	67%	53,500	1.35
60k $AEMA_{70}$-s-$TEGMA_{30}$	AEMA	TEGMA	60,000	70%	72%	59,100	1.31
15k $DMAEMA_{65}$-s-BMA_{30}-s-$AEMA_5$	DMAEMA	BMA	15,000	65%	68%	14,100*	1.33
15k $DMAEMA_{65}$-s-$TEGMA_{30}$-s-$AEMA_5$	DMAEMA	TEGMA	15,000	65%	63%	10,600*	1.60
60k $DMAEMA_{65}$-s-BMA_{30}-s-$AEMA_5$	DMAEMA	BMA	60,000	65%	61%	37,500*	1.39
60k $DMAEMA_{65}$-s-$TEGMA_{30}$-s-$AEMA_5$	DMAEMA	TEGMA	60,000	65%	61%	31,800*	1.35

Figure S18: Synthesis and characterization of AF488-Labelled Polymers. (A) Synthetic strategy to prepare polymers for AF488 conjugation and subsequent imaging studies. (B) Characterization of polymers prepared for conjugation to AF488-NHS Ester. Raw NMR and SEC spectra are provided in the *^{1}H-NMR and SEC of Polymer Library* section of the supplementary information. Where starred (*), it should be noted that DMF-SEC was conducted on deprotected polymers containing 5 mol.% of primary amines, so the SEC values reported for are expected to be under-estimations due to primary amine-column interactions.

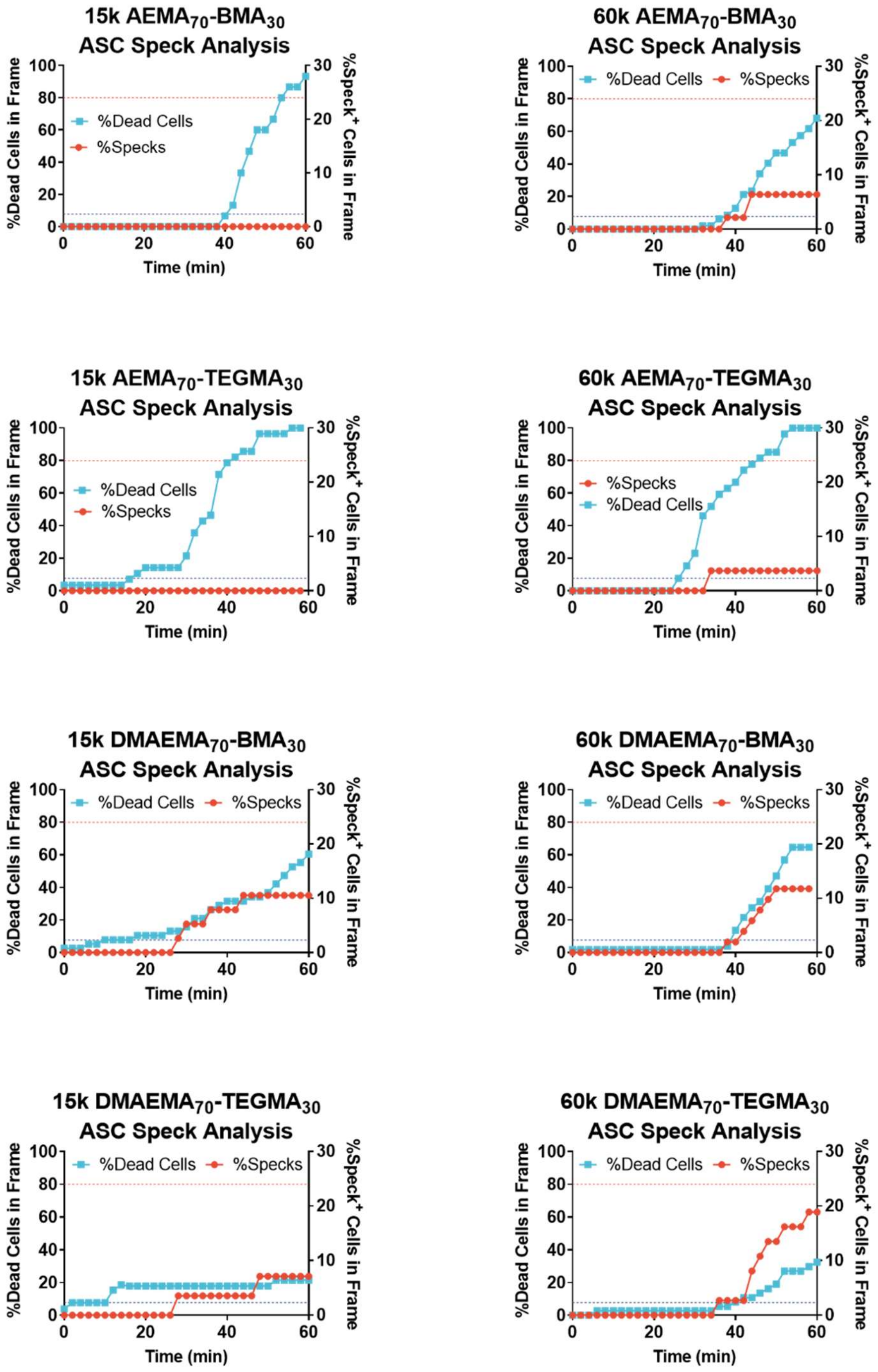

Figure S19: THP-1 ASC-GFP Speck Formation. ASC speck formation and propidium iodide staining of THP-ASC-GFP cells were analyzed in 2 min intervals for 60 min after treatment with the eight polymers of interest at 100 µg/mL. A positive nigericin control is shown in orange, and negative PBS control is shown in purple. Selected results are also presented in **Figure 6A**.

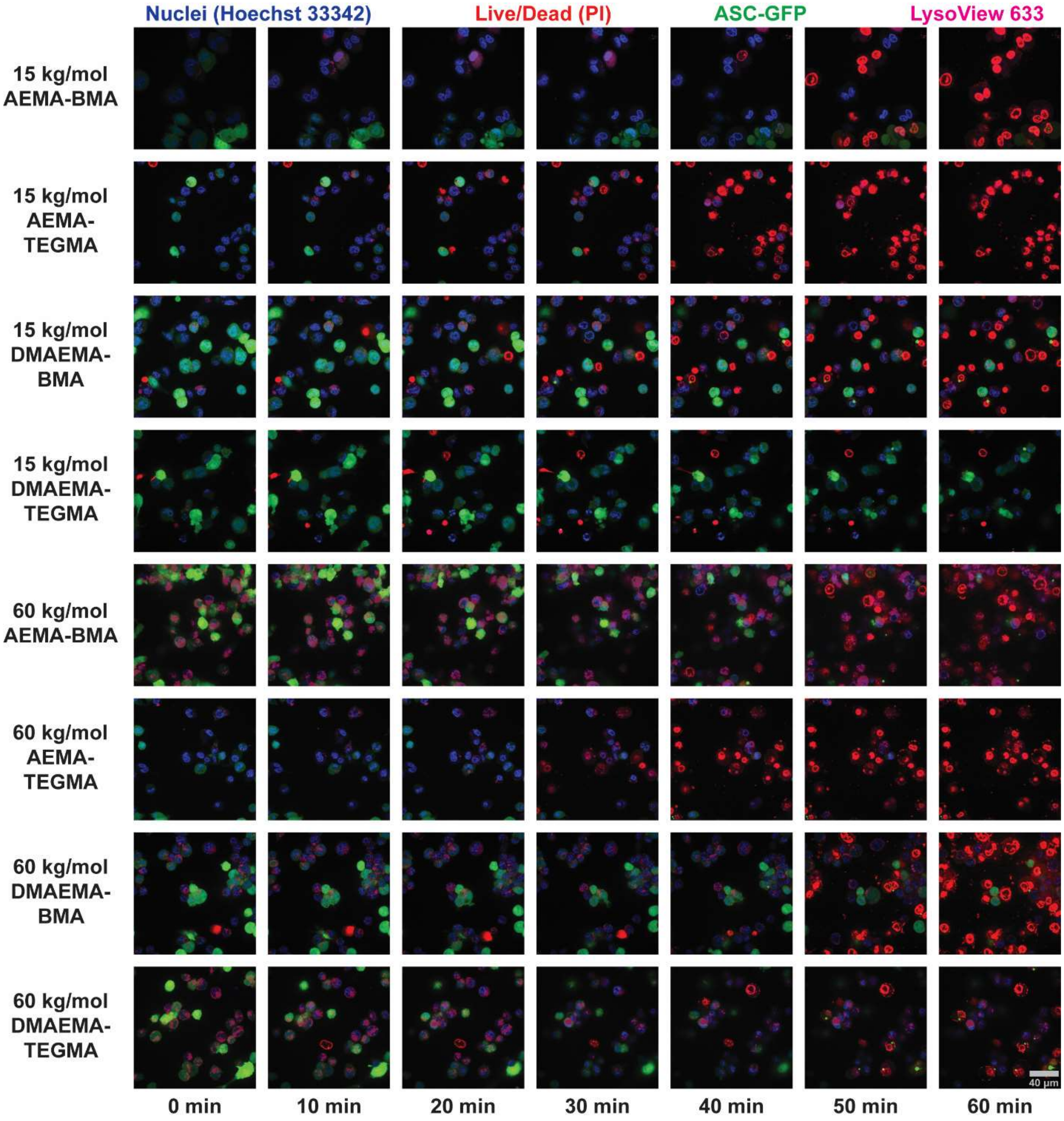

Figure S20: THP-1 ASC-GFP Speck Raw Images. THP-1 ASC-GFP cells were stained with LysoView 633, Propidium Iodide, and Hoechst 33342 and then treated with 100 µg/mL of the indicated polymers. Cells were then imaged every 2 min for 1 h using confocal microscopy (40x oil lens) to evaluate speck formation and cell death. 10 min intervals of selected polymers are shown here, and full videos are available in *Supplementary Video 4*. Images were used to determine results shown in **Figures 6A** and **S19**. Scale bar is representative of all images.

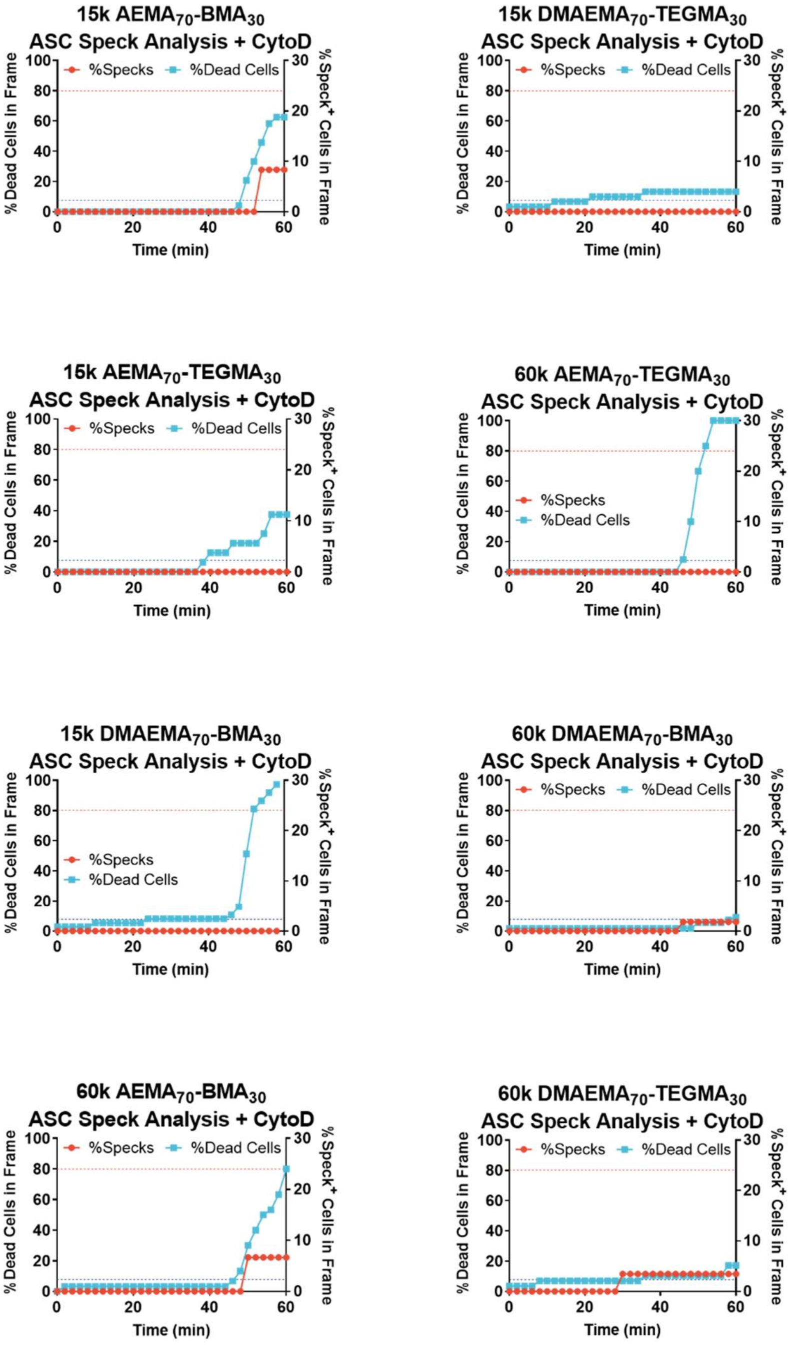

Figure S21: THP-1 ASC-GFP Speck Formation + Cytochalasin D. Cells were pre-treated with cytochalasin D (CytoD), and ASC speck formation and propidium iodide staining were analyzed in 2 min intervals for 60 min after treatment with the eight polymers of interest at 100 µg/mL (as in **Figure S19**). A positive nigericin control is shown in orange, and negative PBS control is shown in purple (both conducted in WT THP-1s). Selected results are also presented in **Figure 6B**.

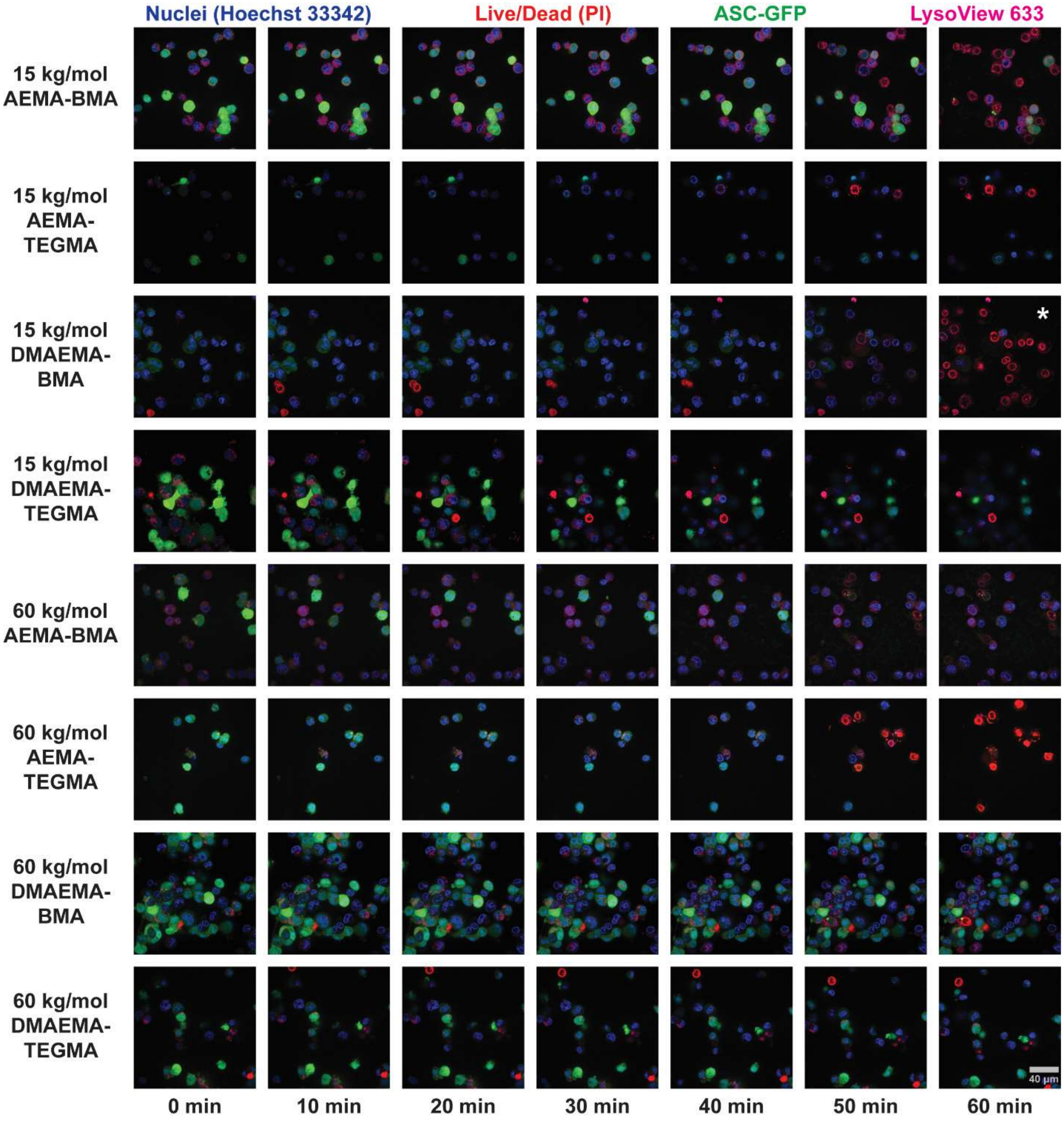

Figure S22: ASC-GFP Speck Raw Images + Cytochalasin D. THP-1 ASC-GFP cells were pre-treated with Cytochalasin D, stained as in **Figure S19**, and treated with 100 μg/mL of the indicated polymers. Cells were then imaged every 2 min for 1 h using confocal microscopy (40x oil lens) to evaluate speck formation and cell death. 10 min intervals of selected polymers are shown here, and full videos are available in *Supplementary Video 5* (* = imaged at 58 min). Images were used to determine results shown in **Figures 6B** and **S21**. Scale bar is representative of all images.

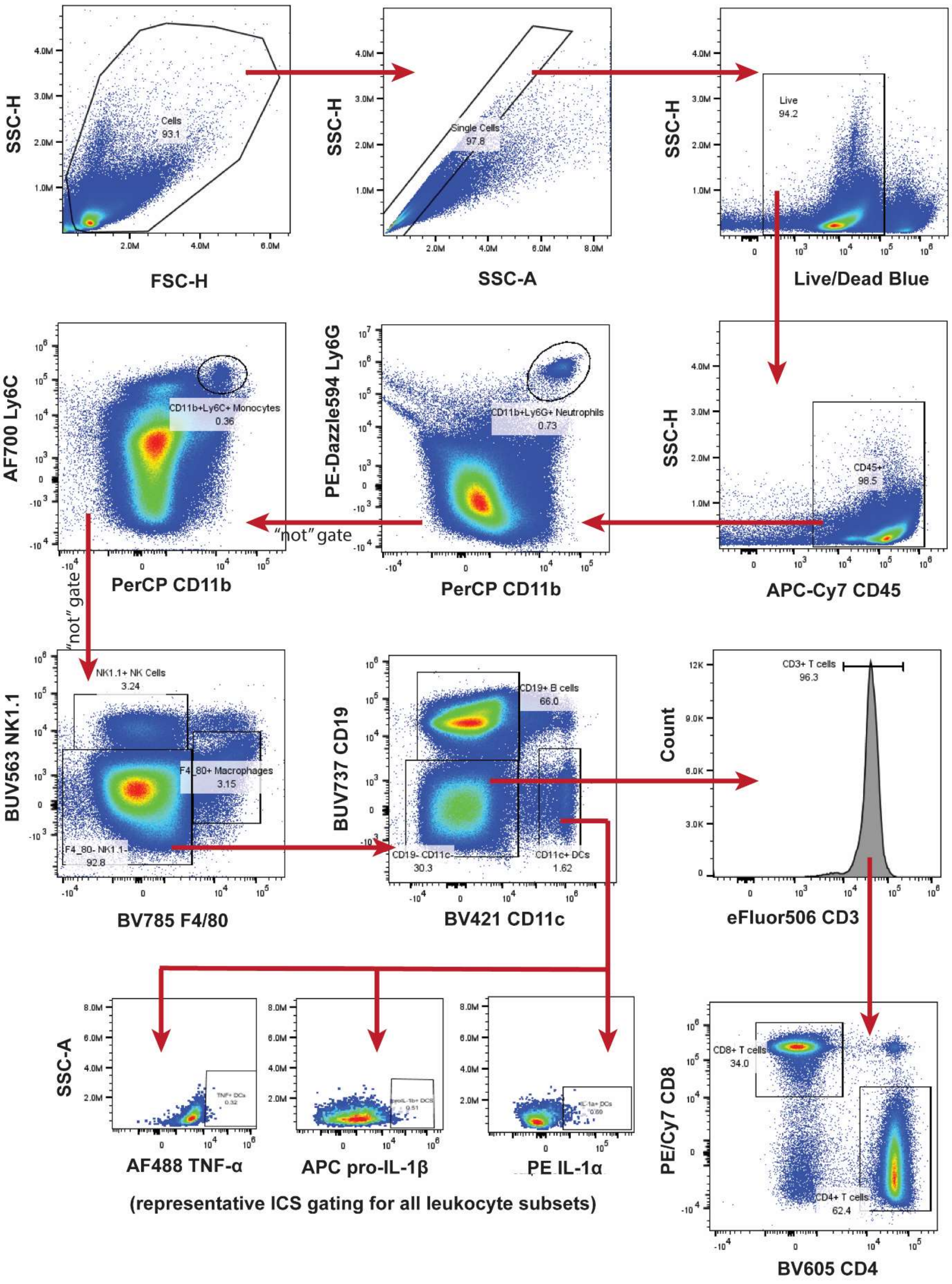

Figure S23: Flow Cytometry Gating Strategy for I.V. Polymer Administration. Flow cytometry gating for the immunophenotyping experiment described in **Figures 7** and **S23-S24**.

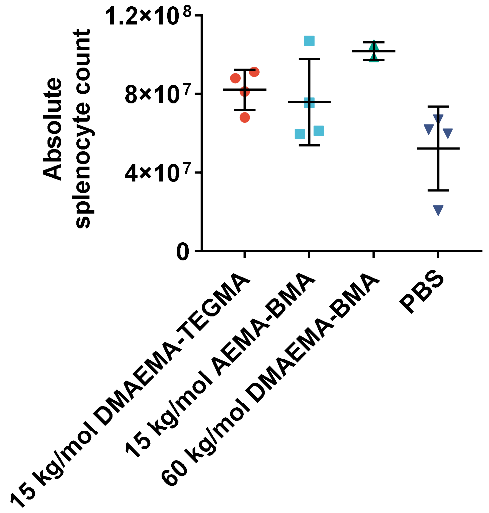

Figure S24: Absolute Cell Counts in Spleen after I.V. Polymer Administration. Absolute splenocyte count 6 h after intravenous injection of 50 μg of the indicated polymers as described in **Figure 7A**. Cells were counted manually using a hemocytometer.

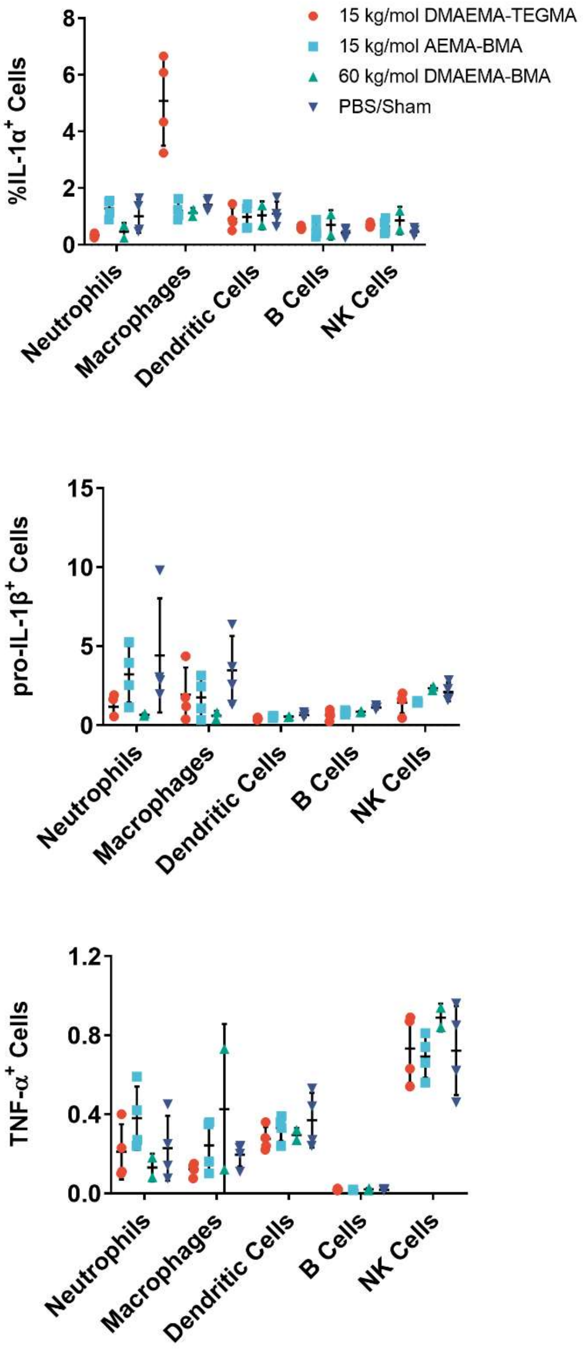

Figure S25: Intracellular IL-1 and TNF-α in Spleen after I.V. Polymer Administration. Splenocytes collected 6 h after I.V. injection of the indicated polymers were immunophenotyped as described in **Figure S22**, and the percentage of IL-1α$^+$, pro-IL-1β$^+$, and TNF-α$^+$ cells in each leukocyte subset were quantified via intracellular cytokine staining and flow cytometry.

^{1}H-NMR and SEC of Polymer Library

1. 7.5 kg/mol poly(AEMA-*s*-BMA)

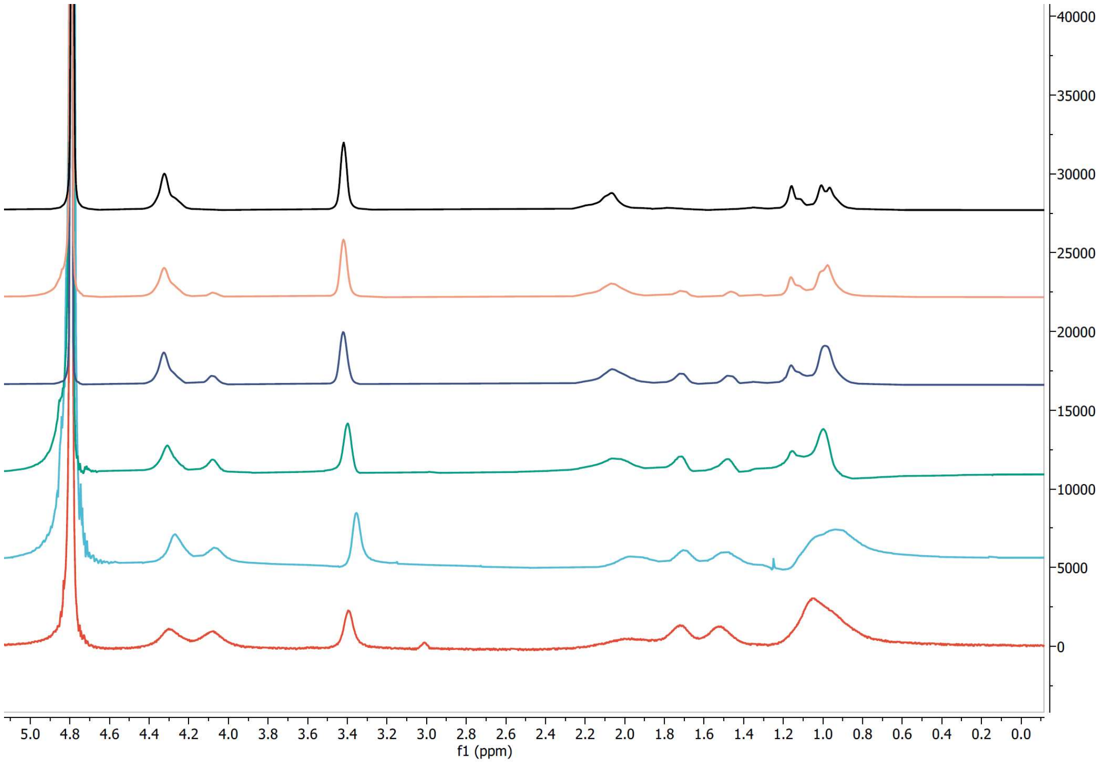

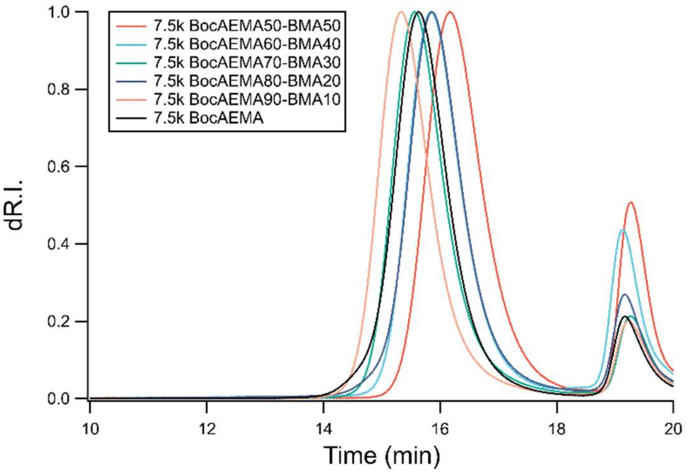

Mon1	Mon2	M_n (th)	%Mon1 (th)	%Mon1 (NMR)	M_n_Boc (SEC)	M_n (SEC)	*Đ* (SEC)
AEMA	BMA	7,500	50%	52%	6,600	5,000	1.22
AEMA	BMA	7,500	60%	62%	8,900	6,800	1.19
AEMA	BMA	7,500	70%	76%	10,700	7,500	1.22
AEMA	BMA	7,500	80%	82%	8,800	5,900	1.23
AEMA	BMA	7,500	90%	91%	14,200	8,800	1.14
AEMA	N/A	7,500	100%	100%	10,900	6,300	1.22

2. 7.5 kg/mol poly(AEMA-*s*-TEGMA)

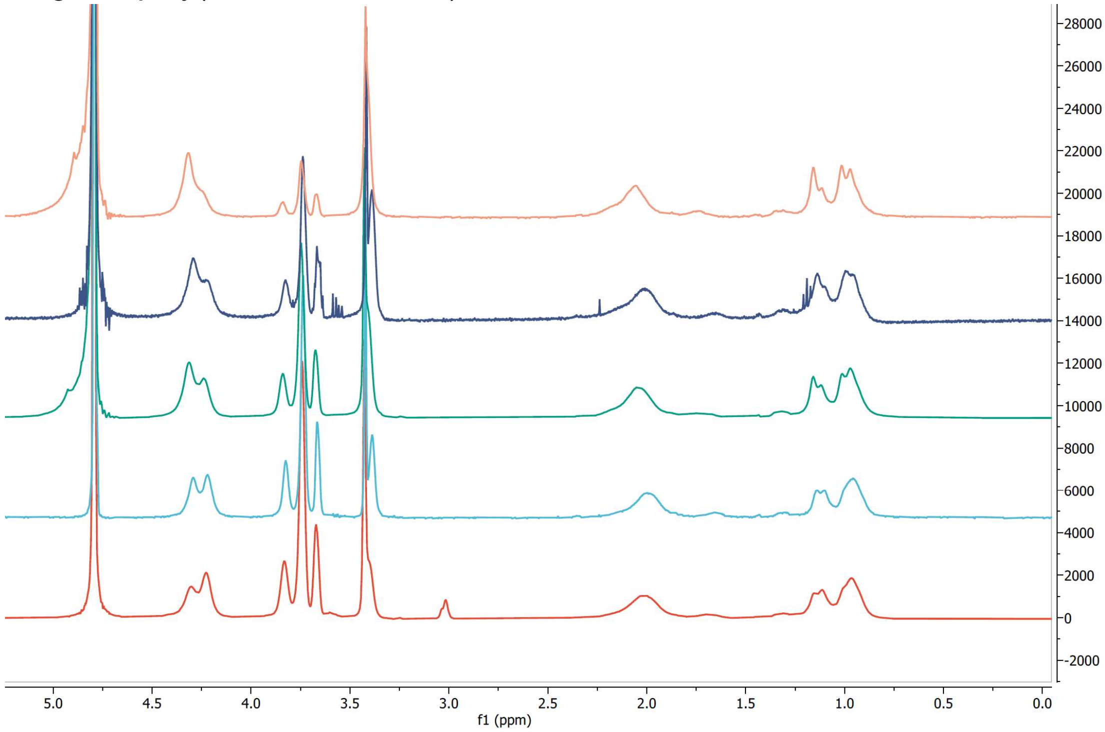

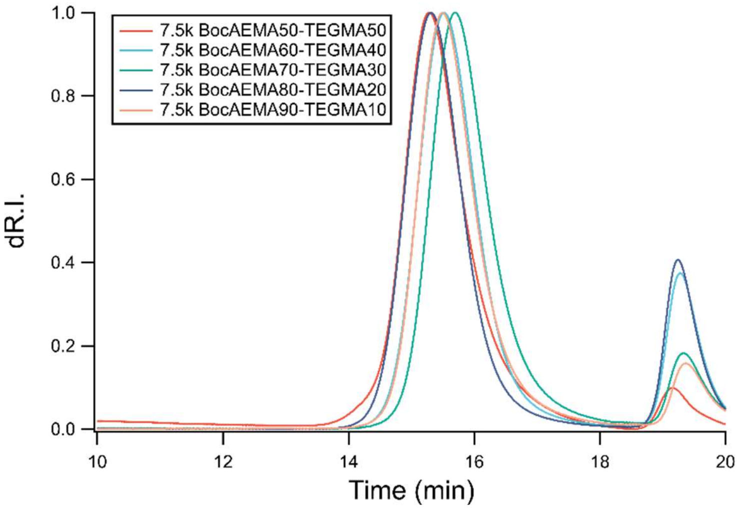

Mon1	Mon2	M_n (th)	%Mon1 (th)	%Mon1 (NMR)	M_n_Boc (SEC)	M_n (SEC)	*Đ* (SEC)
AEMA	TEGMA	7,500	50%	43%	12,700	10,100	1.30
AEMA	TEGMA	7,500	60%	64%	12,000	8,800	1.19
AEMA	TEGMA	7,500	70%	76%	9,500	6,700	1.26
AEMA	TEGMA	7,500	80%	79%	14,400	9,600	1.16
AEMA	TEGMA	7,500	90%	91%	12,000	7,400	1.18

3. 7.5 kg/mol poly(DMAEMA-*s*-BMA)

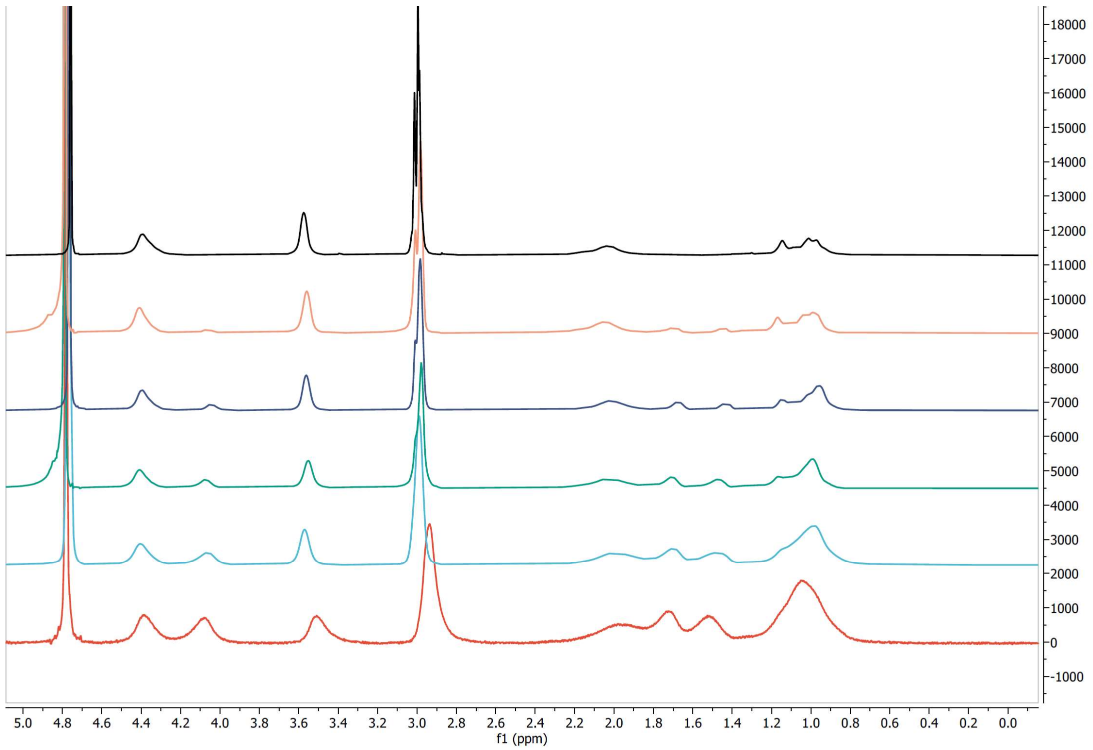

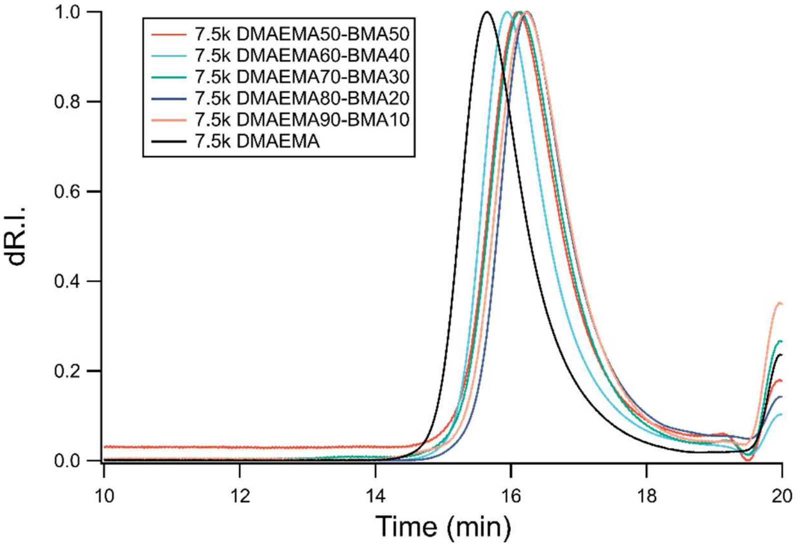

Mon1	Mon2	M_n (th)	%Mon1 (th)	%Mon1 (NMR)	M_n (SEC)	$Đ$ (SEC)
DMAEMA	BMA	7,500	50%	45%	6,000	1.37
DMAEMA	BMA	7,500	60%	59%	7,000	1.35
DMAEMA	BMA	7,500	70%	69%	5,900	1.37
DMAEMA	BMA	7,500	80%	76%	5,600	1.32
DMAEMA	BMA	7,500	90%	91%	5,400	1.37
DMAEMA	N/A	7,500	100%	100%	8,700	1.33

4. 7.5 kg/mol poly(DMAEMA-*s*-TEGMA)

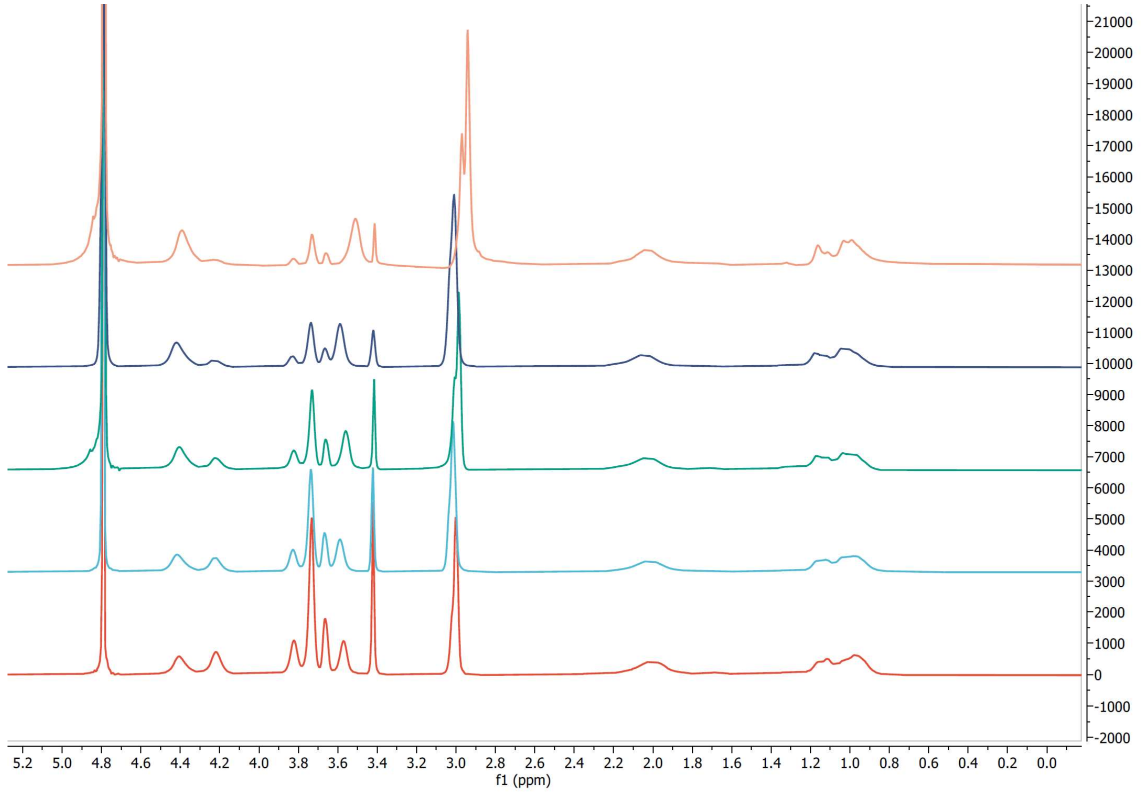

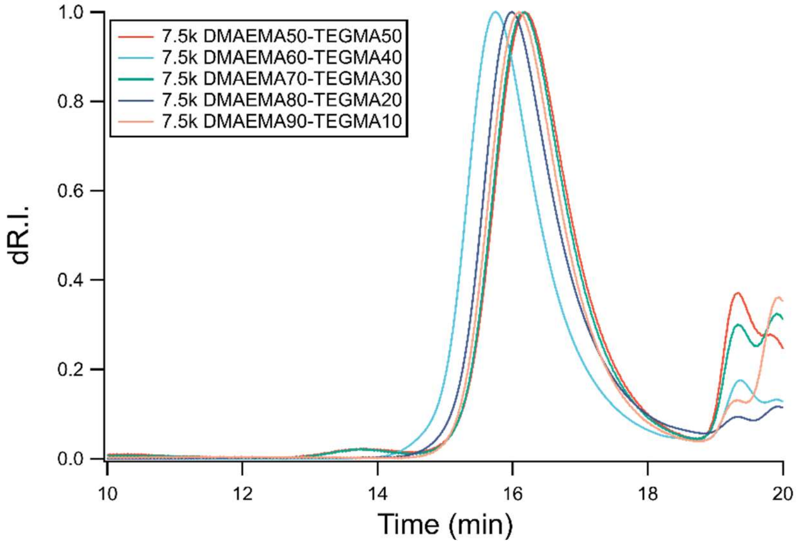

Mon1	Mon2	M_n (th)	%Mon1 (th)	%Mon1 (NMR)	M_n (SEC)	*Đ* (SEC)
DMAEMA	TEGMA	7,500	50%	51%	5,600	1.37
DMAEMA	TEGMA	7,500	60%	60%	7,800	1.39
DMAEMA	TEGMA	7,500	70%	66%	5,700	1.39
DMAEMA	TEGMA	7,500	80%	81%	6,100	1.45
DMAEMA	TEGMA	7,500	90%	86%	5,900	1.39

5. 15 kg/mol poly(AEMA-*s*-BMA)

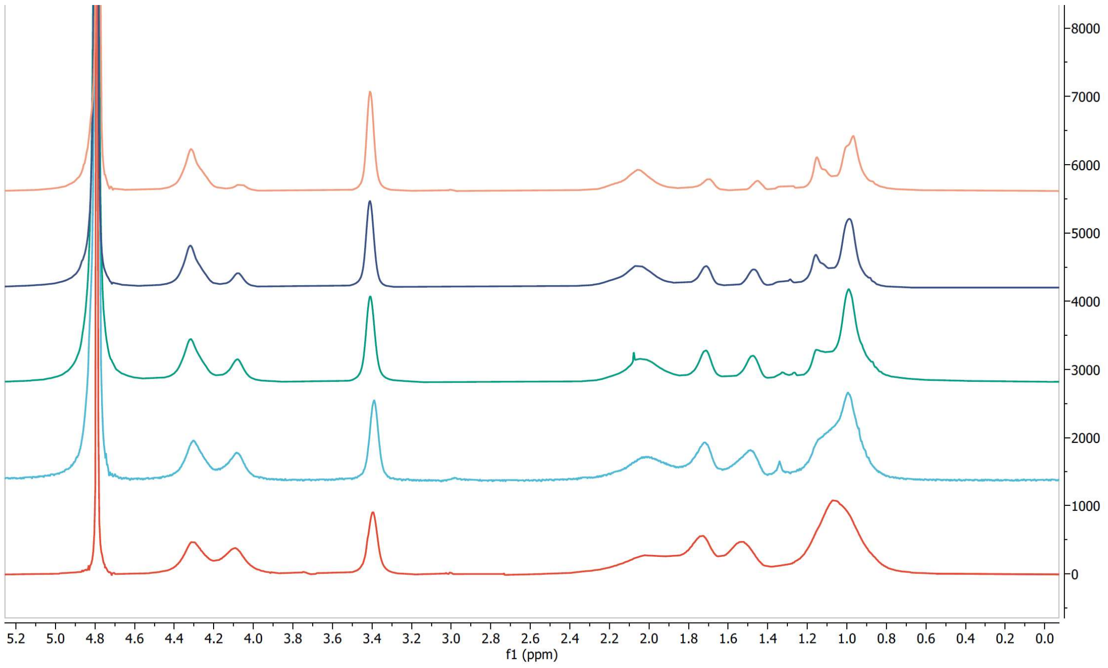

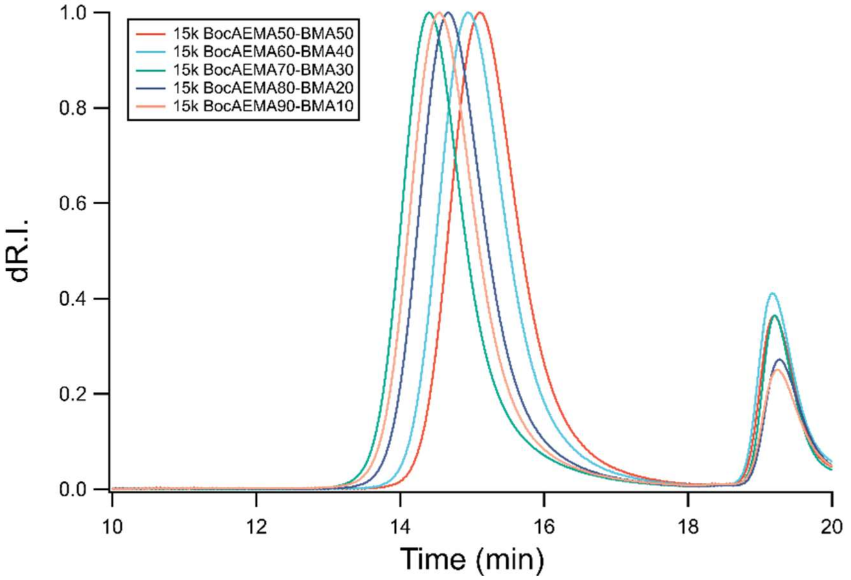

Mon1	Mon2	M_n (th)	%Mon1 (th)	%Mon1 (NMR)	M_n_Boc (SEC)	M_n (SEC)	*Đ* (SEC)
AEMA	BMA	15,000	50%	54%	16,200	12,700	1.17
AEMA	BMA	15,000	60%	63%	18,200	12,700	1.17
AEMA	BMA	15,000	70%	68%	26,800	18,800	1.17
AEMA	BMA	15,000	80%	81%	22,400	14,700	1.17
AEMA	BMA	15,000	90%	91%	25,700	15,700	1.14

6. 15 kg/mol poly(AEMA-*s*-TEGMA)

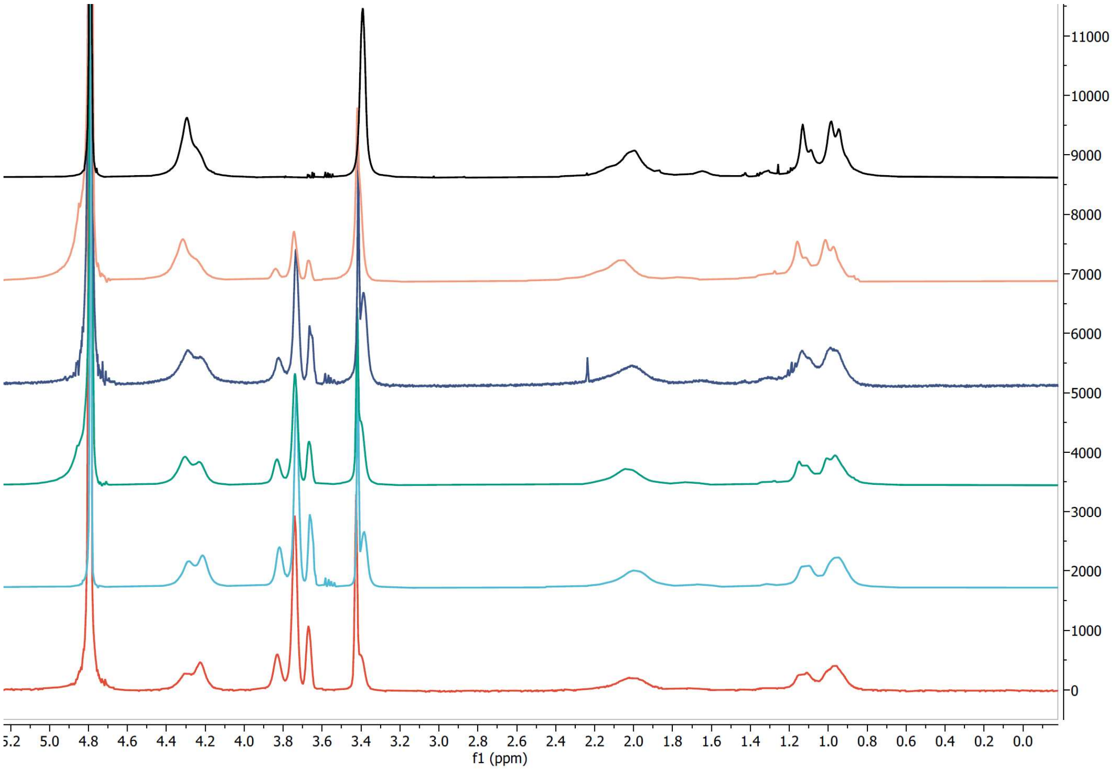

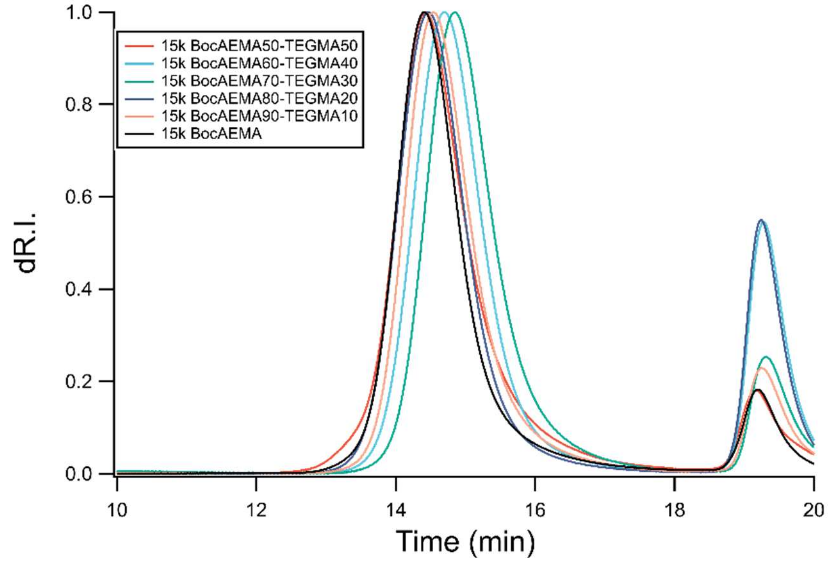

Mon1	Mon2	M_n (th)	%Mon1 (th)	%Mon1 (NMR)	M_n_Boc (SEC)	M_n (SEC)	*Đ* (SEC)
AEMA	TEGMA	15,000	50%	45%	24,900	19,700	1.25
AEMA	TEGMA	15,000	60%	63%	22,700	16,600	1.17
AEMA	TEGMA	15,000	70%	75%	19,000	13,300	1.21
AEMA	TEGMA	15,000	80%	79%	26,700	17,600	1.18
AEMA	TEGMA	15,000	90%	89%	24,600	15,000	1.18
AEMA	N/A	15,000	100%	100%	25,700	14,600	1.25

7. 15 kg/mol poly(DMAEMA-*s*-BMA)

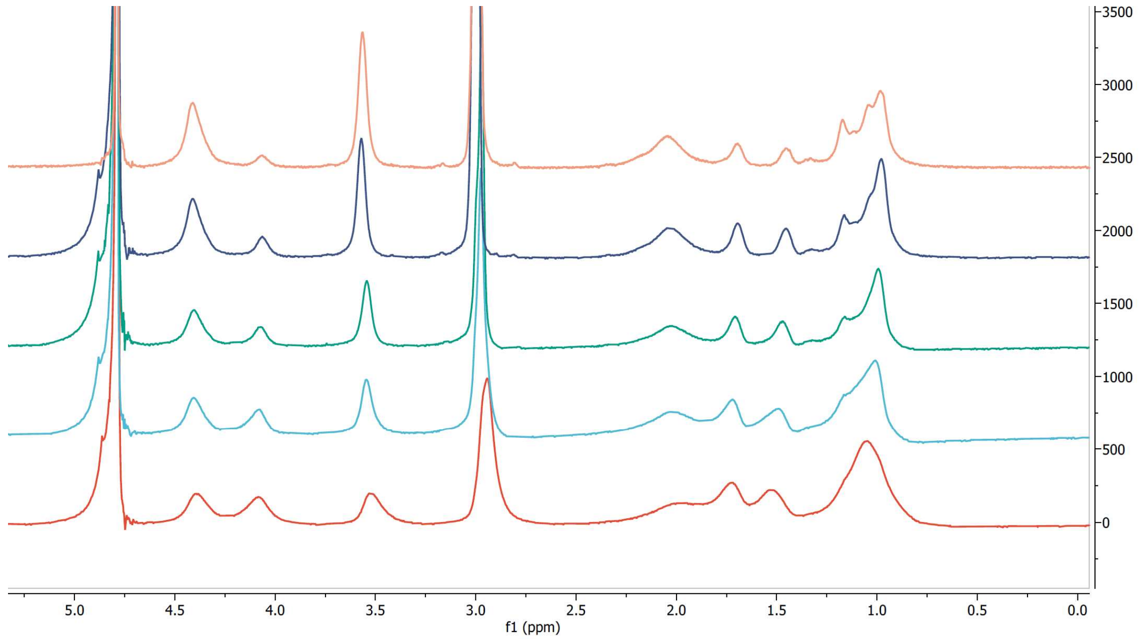

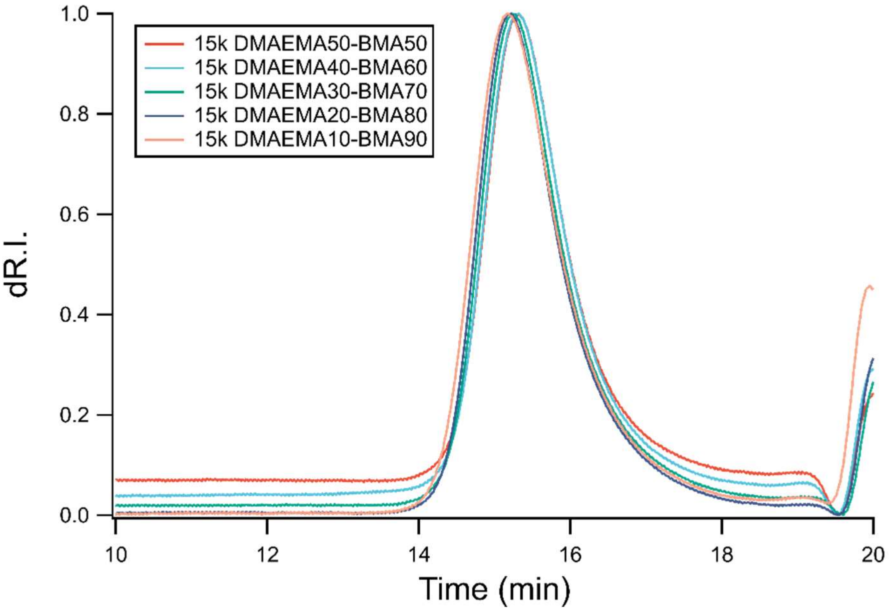

Mon1	Mon2	M_n (th)	%Mon1 (th)	%Mon1 (NMR)	M_n (SEC)	$Đ$ (SEC)
DMAEMA	BMA	15,000	50%	52%	12,300	1.28
DMAEMA	BMA	15,000	60%	65%	11,500	1.35
DMAEMA	BMA	15,000	70%	70%	11,500	1.39
DMAEMA	BMA	15,000	80%	79%	13,200	1.27
DMAEMA	BMA	15,000	90%	93%	13,000	1.32

8. 15 kg/mol poly(DMAEMA-*s*-TEGMA)

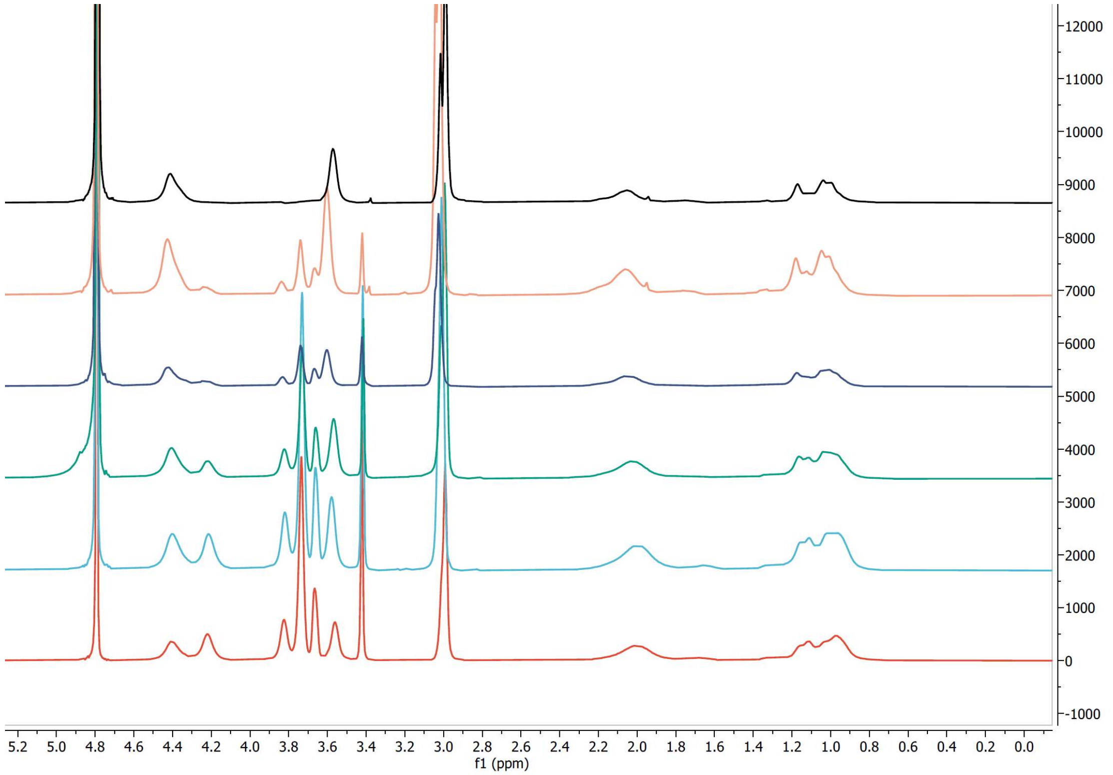

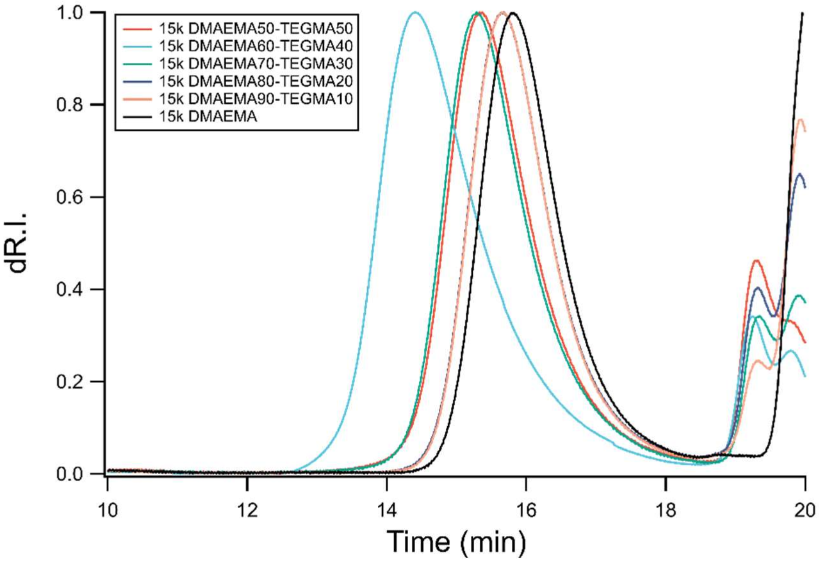

Mon1	Mon2	M_n (th)	%Mon1 (th)	%Mon1 (NMR)	M_n (SEC)	*Đ* (SEC)
DMAEMA	TEGMA	15,000	50%	50%	11,400	1.36
DMAEMA	TEGMA	15,000	60%	55%	18,700	1.56
DMAEMA	TEGMA	15,000	70%	64%	11,500	1.37
DMAEMA	TEGMA	15,000	80%	79%	9,300	1.20
DMAEMA	TEGMA	15,000	90%	90%	9,700	1.26
DMAEMA	N/A	15,000	100%	100%	10,800	1.32

9. 30 kg/mol poly(AEMA-*s*-BMA)

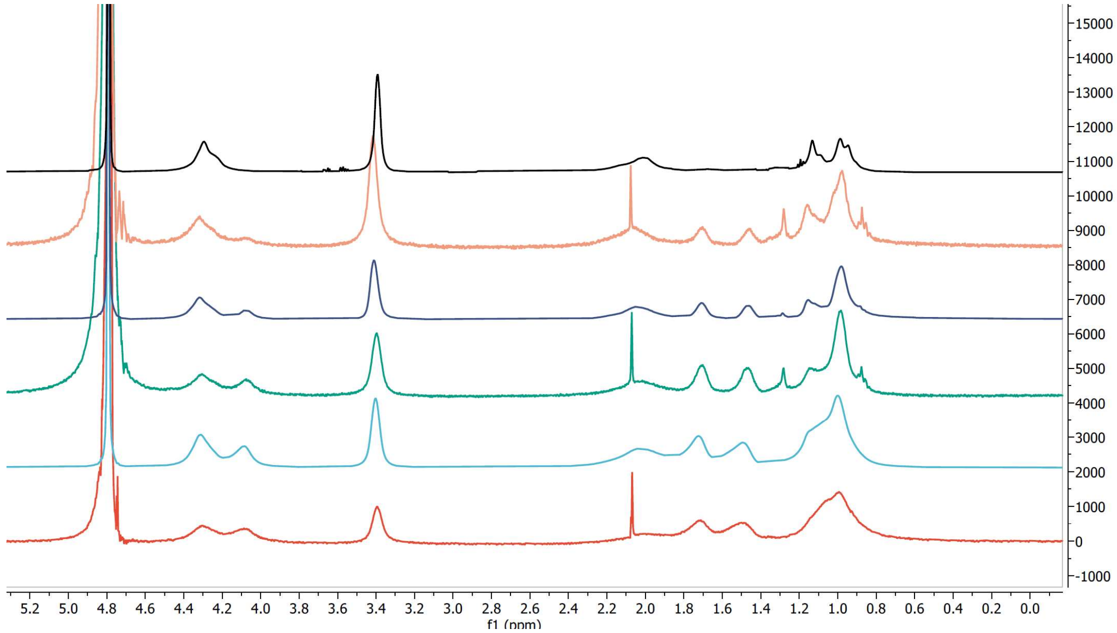

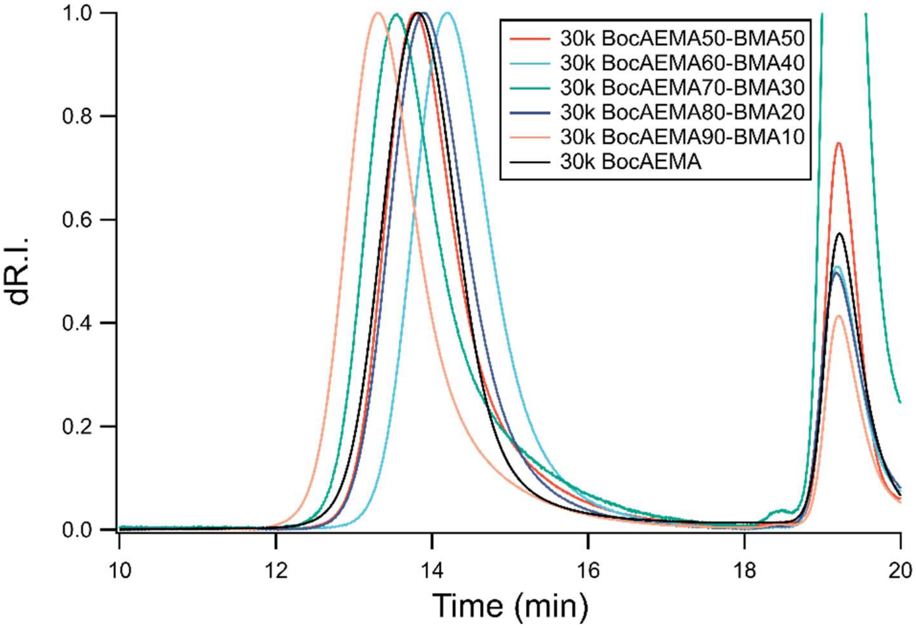

Mon1	Mon2	M_n (th)	%Mon1 (th)	%Mon1 (NMR)	M_n_Boc (SEC)	M_n (SEC)	*Đ* (SEC)
AEMA	BMA	30,000	50%	44%	40,400	31,800	1.20
AEMA	BMA	30,000	60%	62%	31,300	23,200	1.19
AEMA	BMA	30,000	70%	65%	40,000	27,900	1.36
AEMA	BMA	30,000	80%	83%	38,400	25,100	1.20
AEMA	BMA	30,000	90%	92%	56,900	34,700	1.22
AEMA	N/A	30,000	100%	100%	44,100	25,000	1.16

10. 30 kg/mol poly(AEMA-*s*-TEGMA)

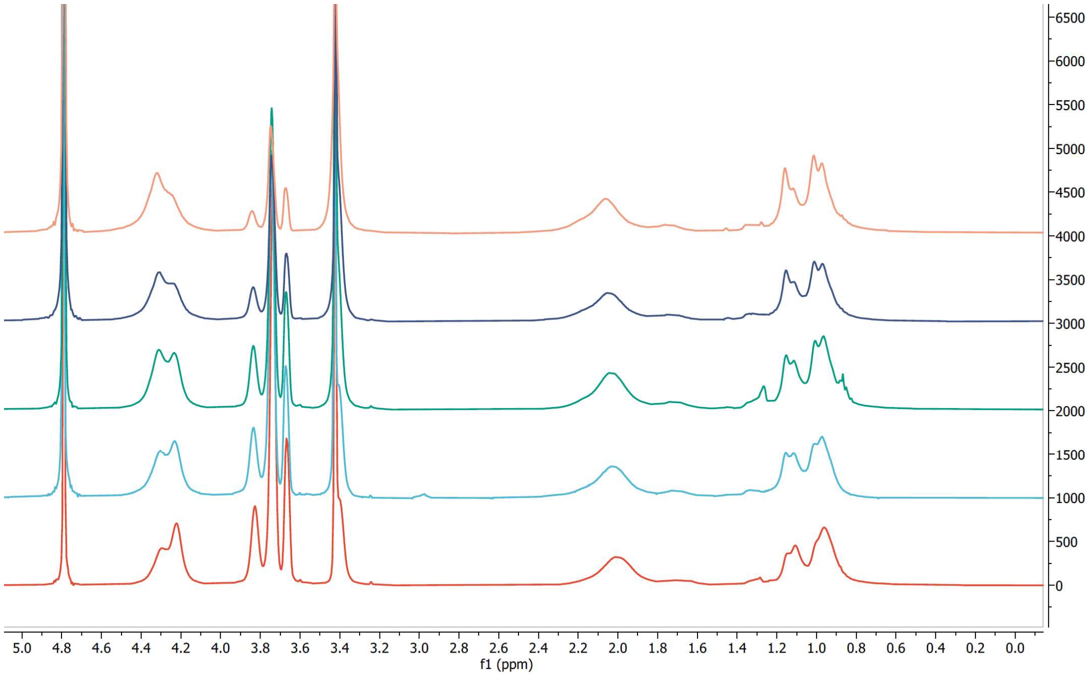

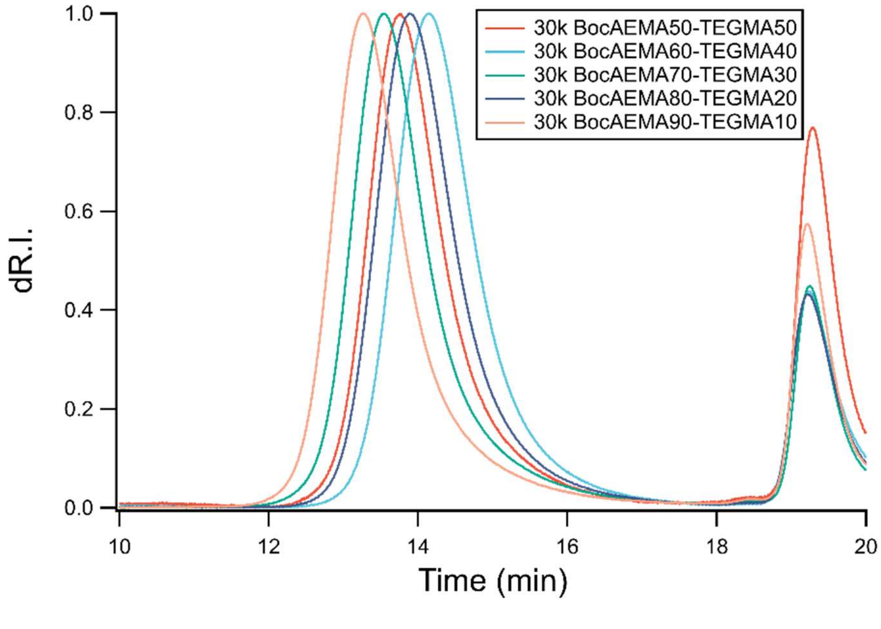

Mon1	Mon2	M_n (th)	%Mon1 (th)	%Mon1 (NMR)	M_n_Boc (SEC)	M_n (SEC)	*Đ* (SEC)
AEMA	TEGMA	30,000	50%	57%	41,200	32,400	1.22
AEMA	TEGMA	30,000	60%	66%	30,900	22,300	1.24
AEMA	TEGMA	30,000	70%	74%	48,300	33,700	1.22
AEMA	TEGMA	30,000	80%	82%	36,300	23,700	1.25
AEMA	TEGMA	30,000	90%	91%	56,400	34,400	1.26

11. 30 kg/mol poly(DMAEMA-*s*-BMA)

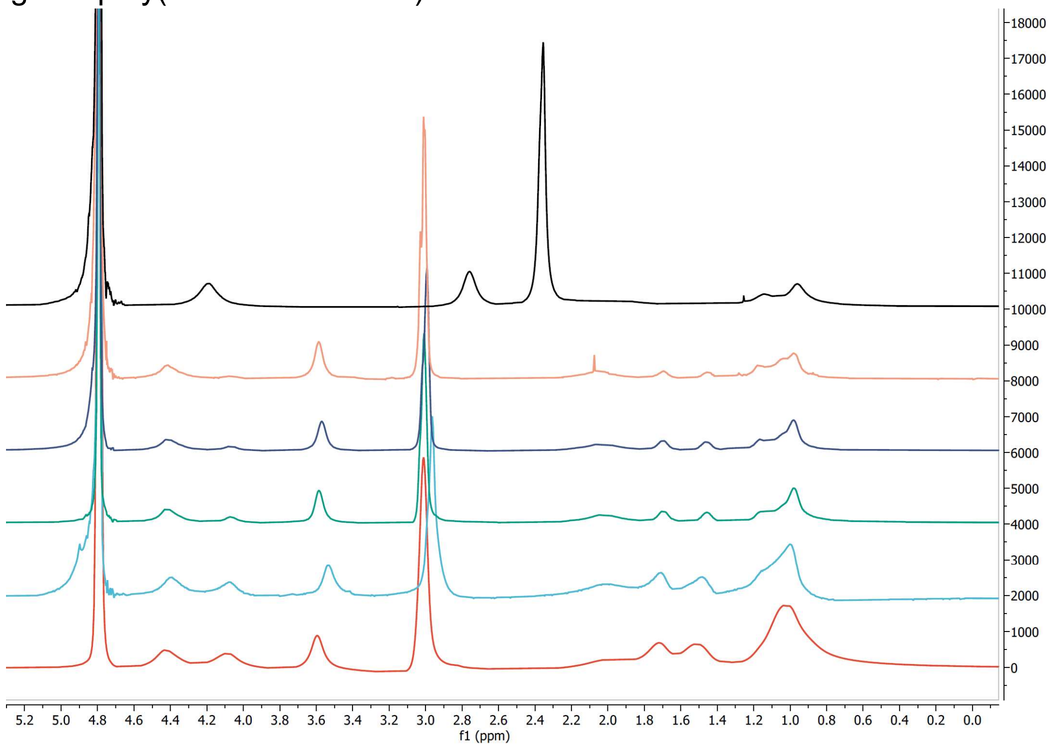

**Note: The 30 kg/mol $DMAEMA_{100}$ polymer was deprotonated prior to ^{1}H-NMR, accounting for the differences observed in its spectrum.

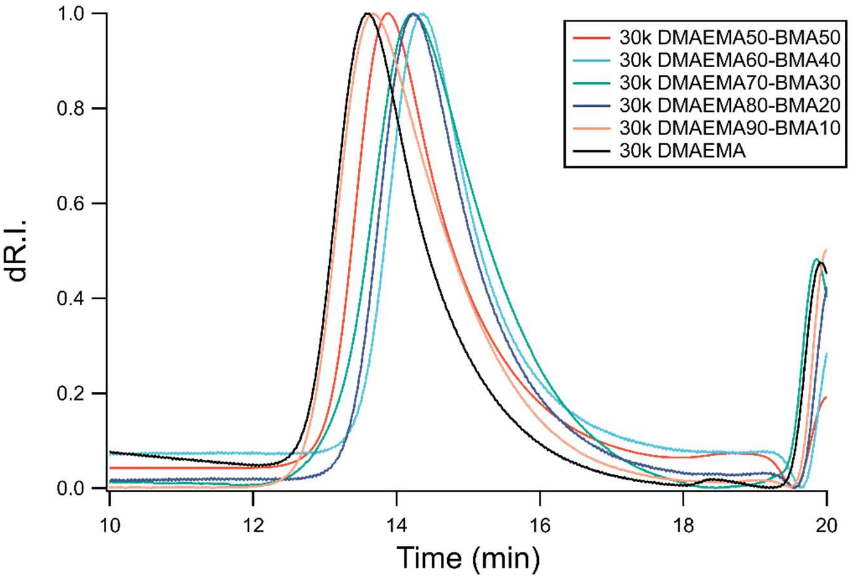

Mon1	Mon2	M_n (th)	%Mon1 (th)	%Mon1 (NMR)	M_n (SEC)	*Đ* (SEC)
DMAEMA	BMA	30,000	50%	43%	30,900	1.35
DMAEMA	BMA	30,000	60%	62%	23,600	1.31
DMAEMA	BMA	30,000	70%	73%	22,200	1.53
DMAEMA	BMA	30,000	80%	83%	24,000	1.37
DMAEMA	BMA	30,000	90%	86%	30,700	1.49
DMAEMA	N/A	30,000	100%	100%	36,900	1.35

12. 30 kg/mol poly(DMAEMA-*s*-TEGMA)

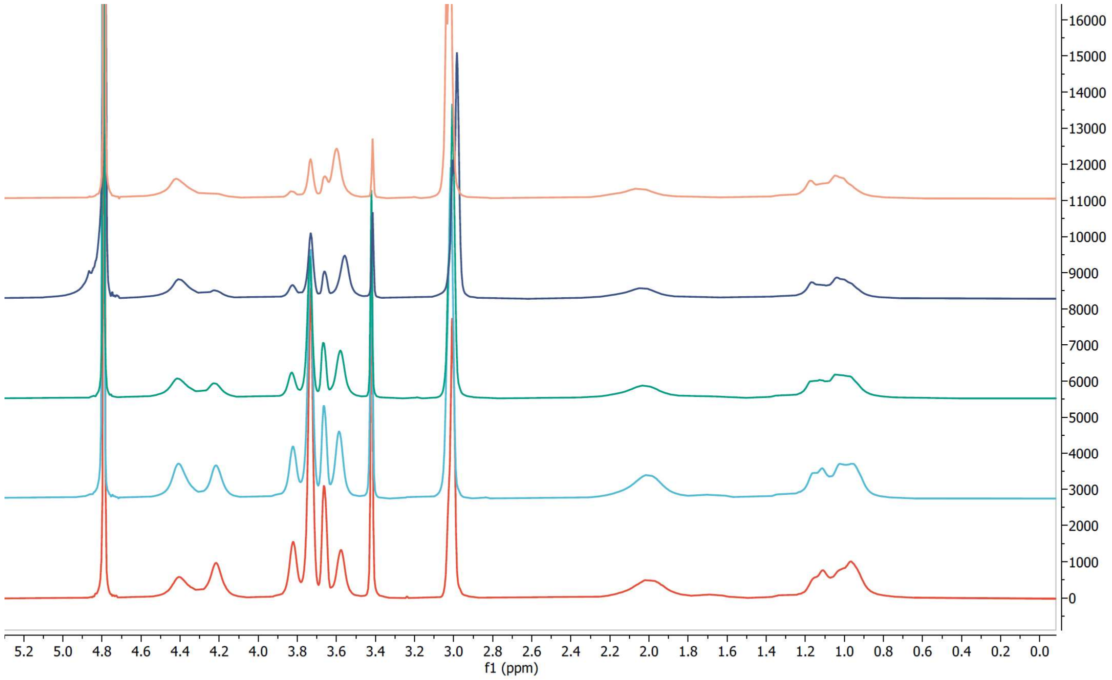

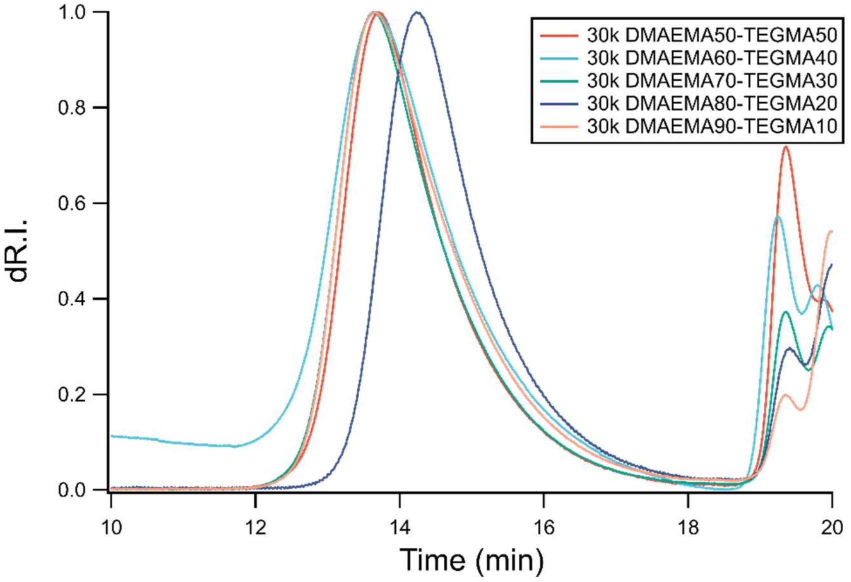

Mon1	Mon2	M_n (th)	%Mon1 (th)	%Mon1 (NMR)	M_n (SEC)	$Đ$ (SEC)
DMAEMA	TEGMA	30,000	50%	50%	32,000	1.47
DMAEMA	TEGMA	30,000	60%	56%	33,900	1.51
DMAEMA	TEGMA	30,000	70%	68%	34,400	1.43
DMAEMA	TEGMA	30,000	80%	74%	21,100	1.53
DMAEMA	TEGMA	30,000	90%	85%	30,800	1.53

13.45 kg/mol poly(AEMA-*s*-BMA)

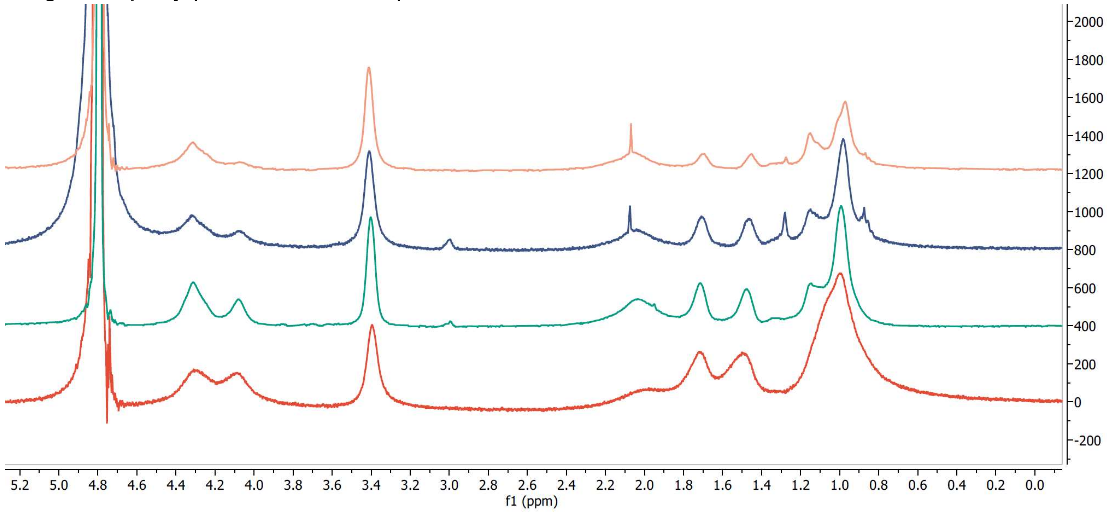

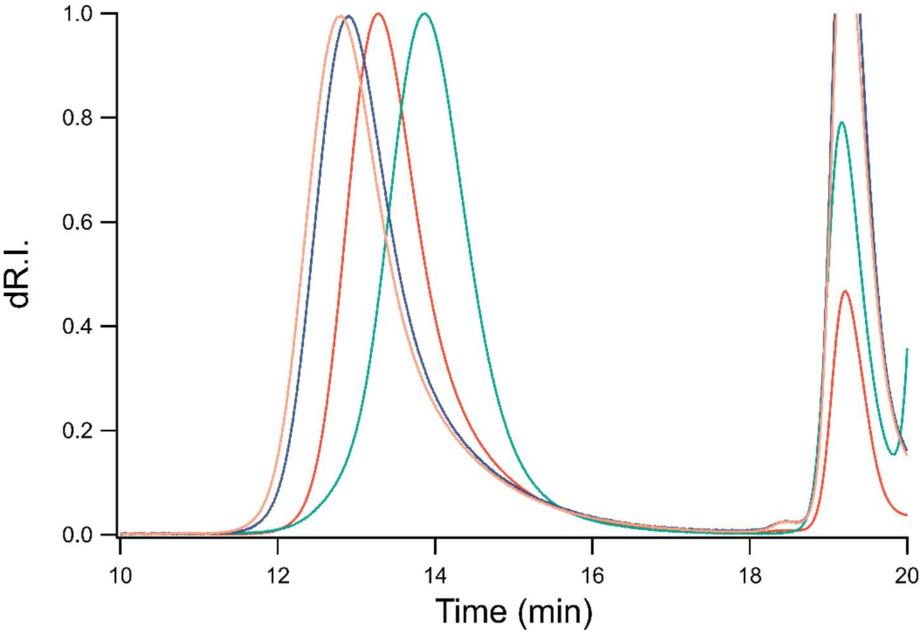

Mon1	Mon2	M_n (th)	%Mon1 (th)	%Mon1 (NMR)	M_n_Boc (SEC)	M_n (SEC)	*Đ* (SEC)
AEMA	BMA	45,000	50%	42%	53,700	42,200	1.28
AEMA	BMA	45,000	70%	70%	41,800	29,200	1.20
AEMA	BMA	45,000	80%	80%	63,400	41,400	1.40
AEMA	BMA	45,000	90%	92%	68,800	41,900	1.39

14.45 kg/mol poly(AEMA-*s*-TEGMA)

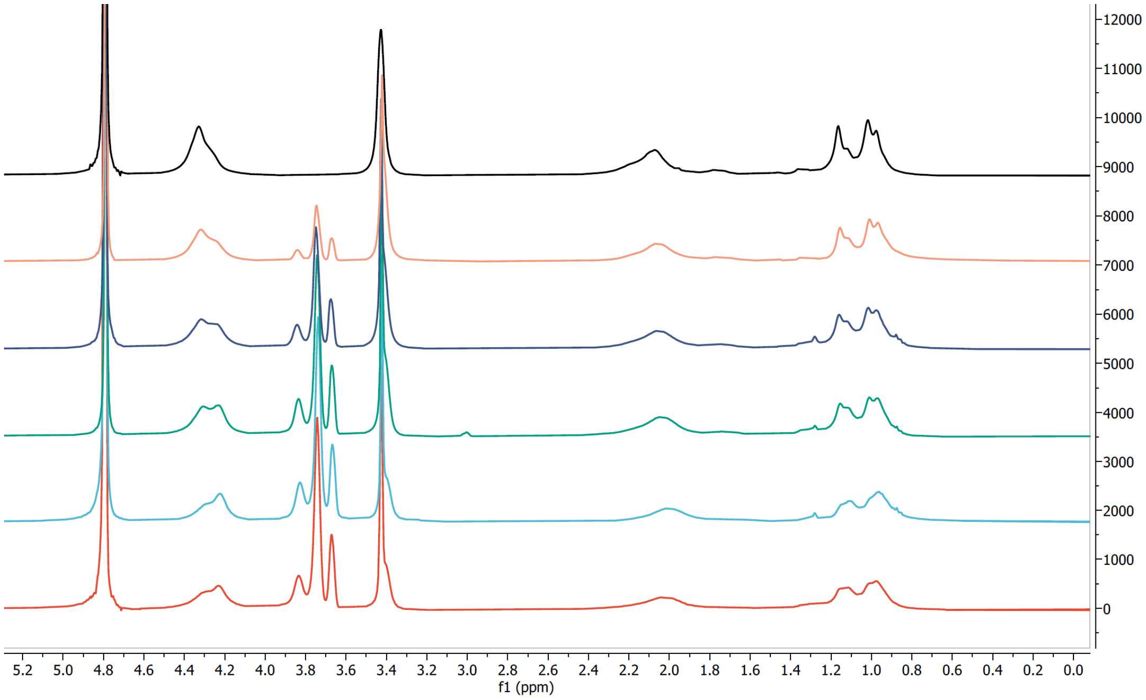

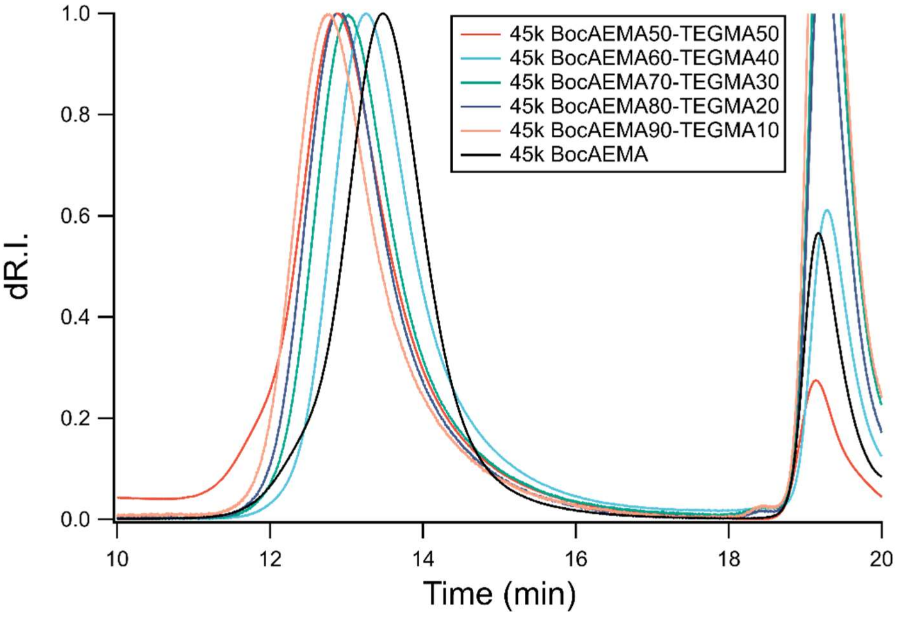

Mon1	Mon2	M_n (th)	%Mon1 (th)	%Mon1 (NMR)	M_n_Boc (SEC)	M_n (SEC)	*Đ* (SEC)
AEMA	TEGMA	45,000	50%	53%	72,600	53,800	1.40
AEMA	TEGMA	45,000	60%	57%	52,800	41,500	1.34
AEMA	TEGMA	45,000	70%	72%	55,800	38,900	1.47
AEMA	TEGMA	45,000	80%	81%	71,200	46,500	1.29
AEMA	TEGMA	45,000	90%	91%	76,800	46,800	1.32
AEMA	N/A	45,000	100%	100%	58,900	33,300	1.18

15.45 kg/mol poly(DMAEMA-s-BMA)

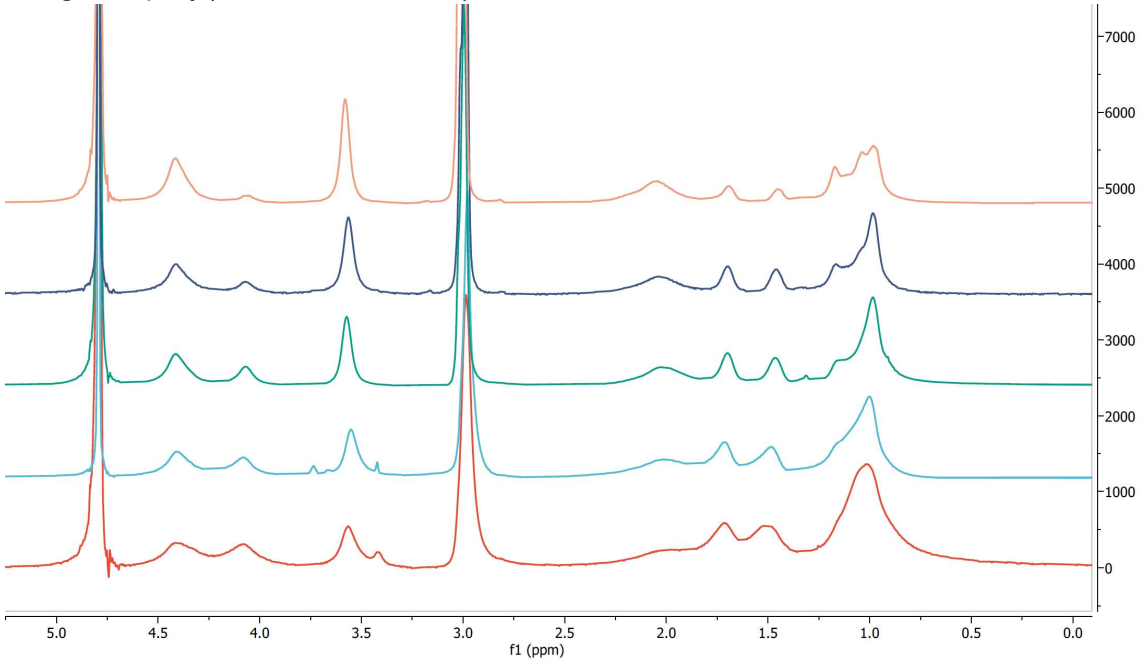

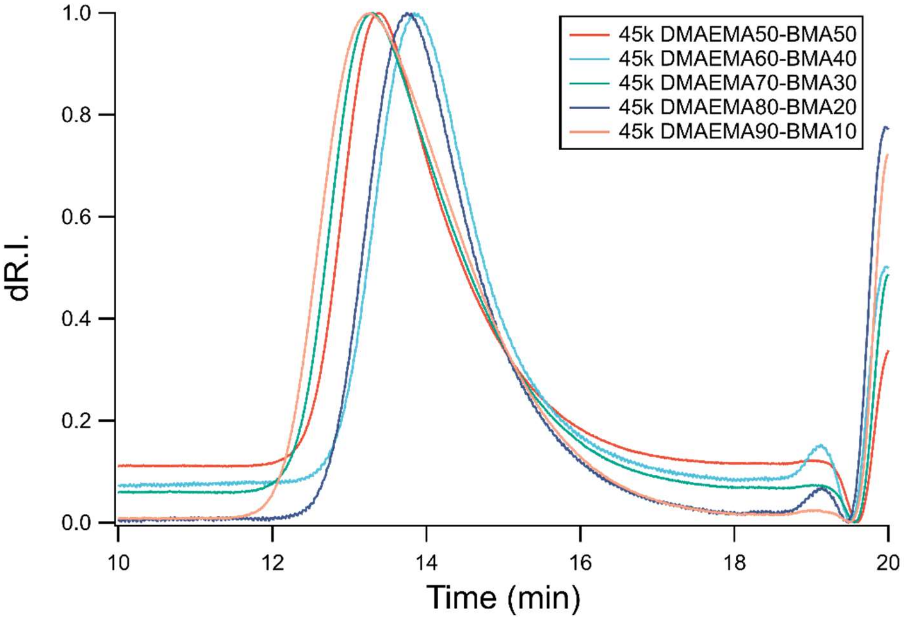

Mon1	Mon2	M_n (th)	%Mon1 (th)	%Mon1 (NMR)	M_n (SEC)	$Đ$ (SEC)
DMAEMA	BMA	45,000	50%	48%	39,600	1.46
DMAEMA	BMA	45,000	60%	61%	34,400	1.32
DMAEMA	BMA	45,000	70%	67%	38,500	1.58
DMAEMA	BMA	45,000	80%	82%	32,000	1.47
DMAEMA	BMA	45,000	90%	91%	38,200	1.66

16.45 kg/mol poly(DMAEMA-*s*-TEGMA)

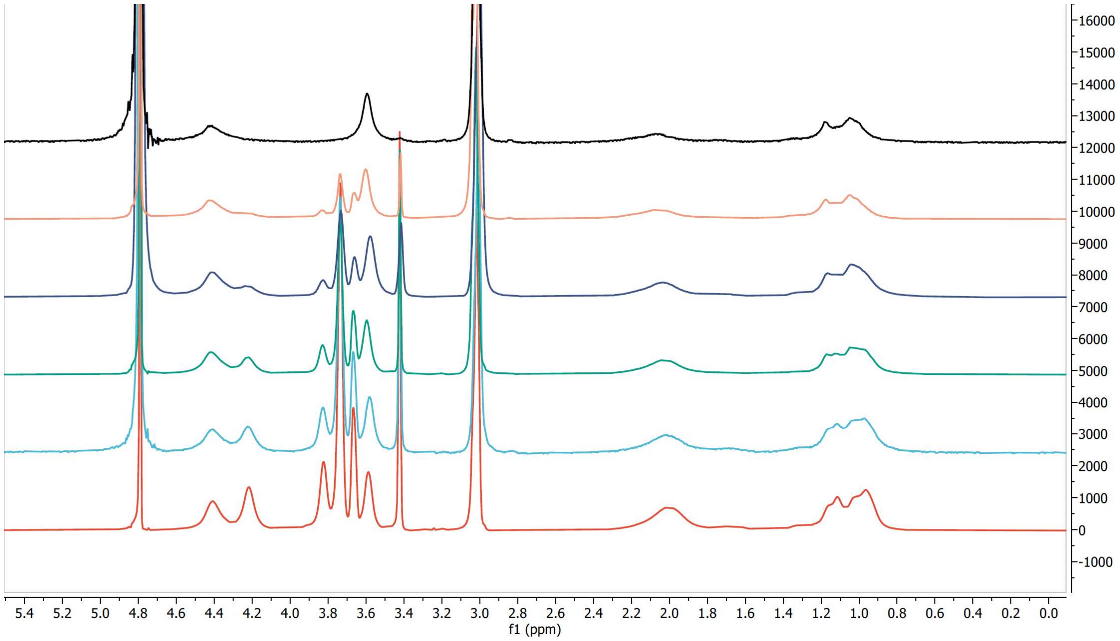

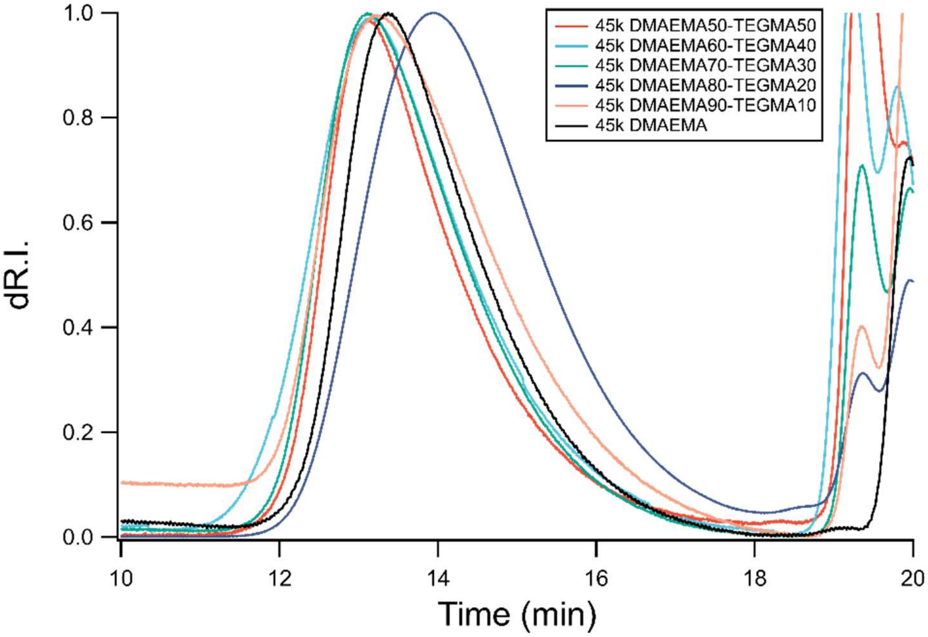

Mon1	Mon2	M_n (th)	%Mon1 (th)	%Mon1 (NMR)	M_n (SEC)	*Đ* (SEC)
DMAEMA	TEGMA	45,000	50%	51%	42,900	1.65
DMAEMA	TEGMA	45,000	60%	54%	46,100	1.70
DMAEMA	TEGMA	45,000	70%	68%	42,800	1.66
DMAEMA	TEGMA	45,000	80%	76%	35,100	1.43
DMAEMA	TEGMA	45,000	90%	83%	42,500	1.57
DMAEMA	N/A	45,000	100%	100%	40,000	1.51

17. 60 kg/mol poly(AEMA-*s*-BMA)

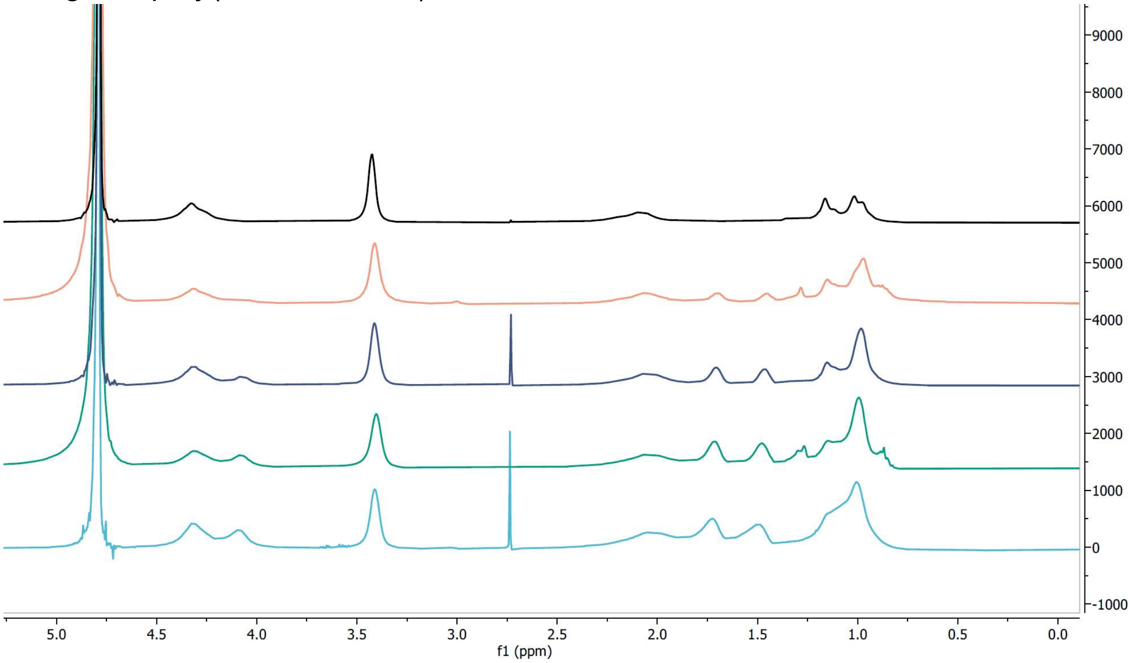

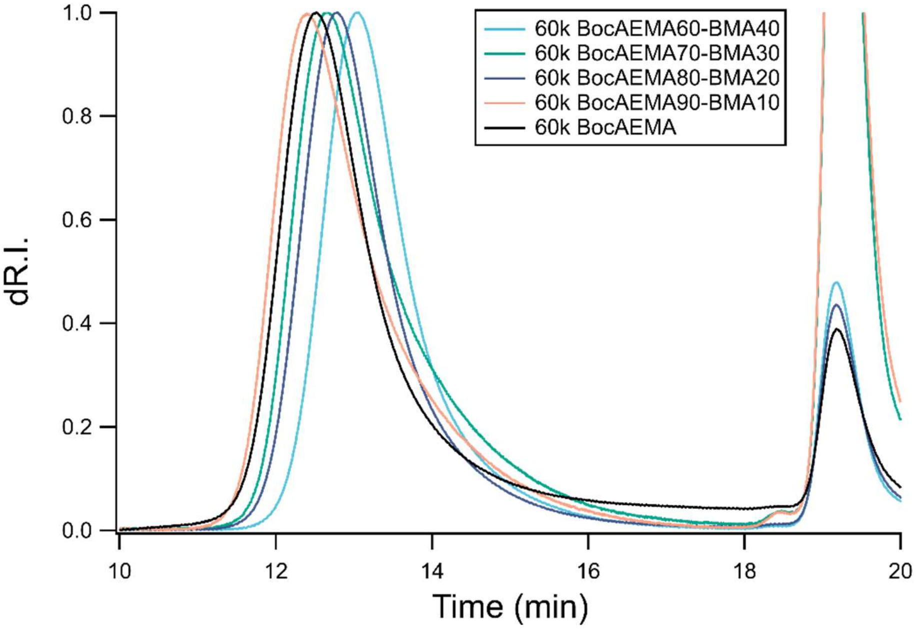

Mon1	Mon2	M_n (th)	%Mon1 (th)	%Mon1 (NMR)	M_n_Boc (SEC)	M_n (SEC)	$Đ$ (SEC)
AEMA	BMA	60,000	60%	60%	66,900	49,500	1.25
AEMA	BMA	60,000	70%	67%	76,800	53,500	1.35
AEMA	BMA	60,000	80%	82%	81,100	52,900	1.26
AEMA	BMA	60,000	90%	88%	76,100	46,300	1.58
AEMA	N/A	60,000	100%	100%	90,100	50,900	1.23

18. 60 kg/mol poly(AEMA-*s*-TEGMA)

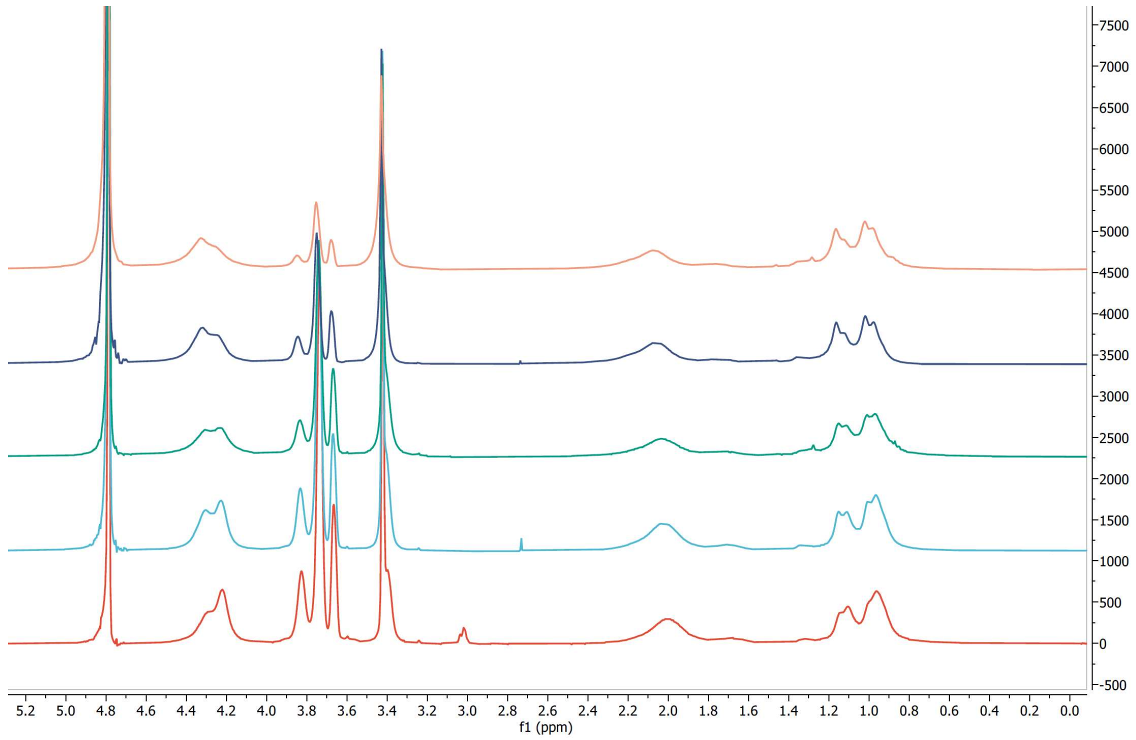

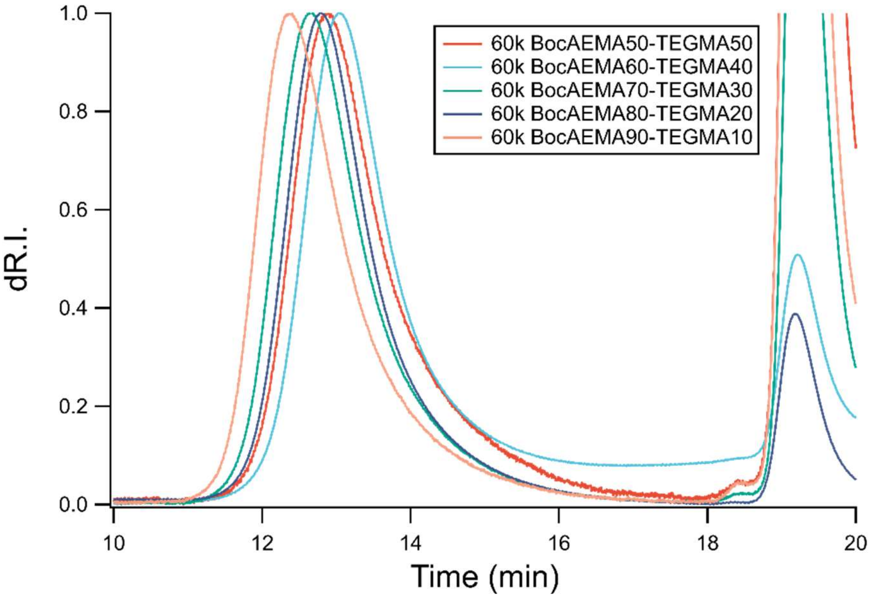

Mon1	Mon2	M_n (th)	%Mon1 (th)	%Mon1 (NMR)	M_n_Boc (SEC)	M_n (SEC)	*Đ* (SEC)
AEMA	TEGMA	60,000	50%	57%	68,800	54,000	1.31
AEMA	TEGMA	60,000	60%	66%	66,700	49,300	1.26
AEMA	TEGMA	60,000	70%	72%	85,000	59,100	1.31
AEMA	TEGMA	60,000	80%	82%	73,000	47,600	1.37
AEMA	TEGMA	60,000	90%	90%	106,200	64,600	1.27

19.60 kg/mol poly(DMAEMA-*s*-BMA)

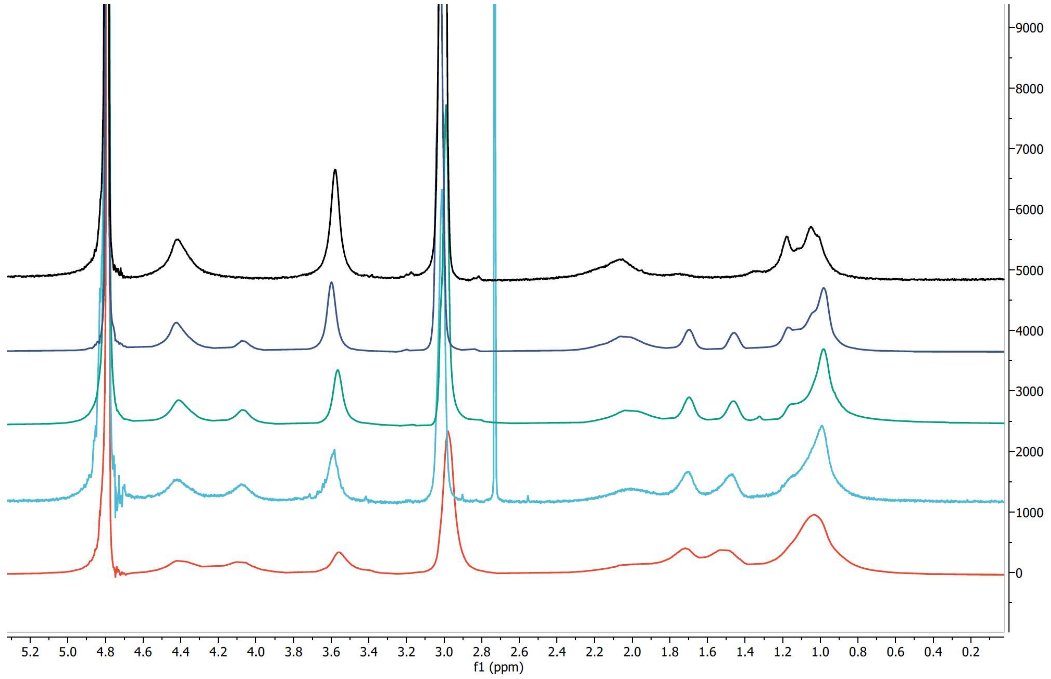

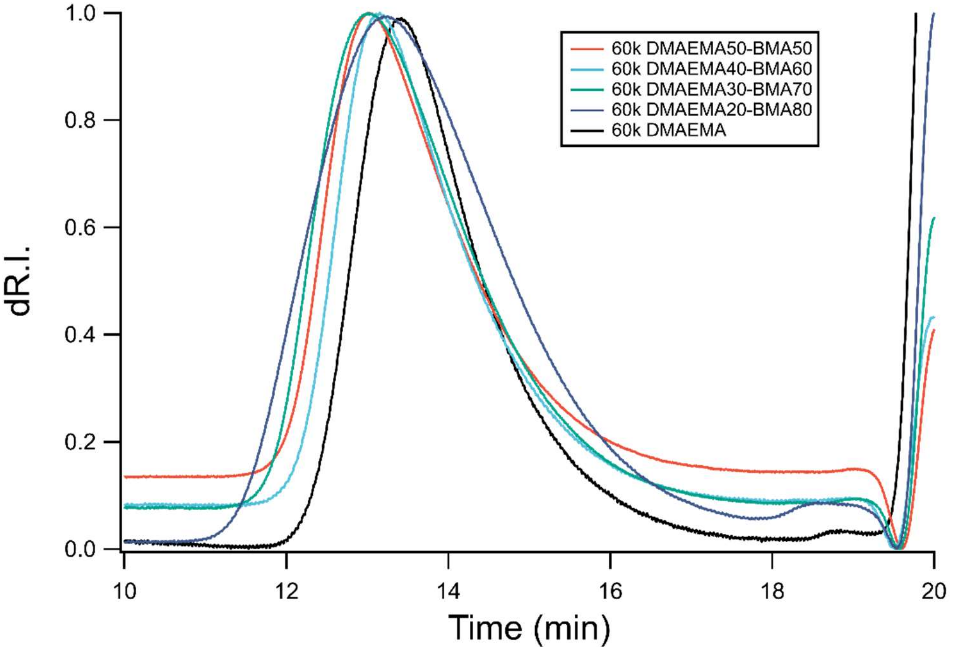

Mon1	Mon2	M_n (th)	%Mon1 (th)	%Mon1 (NMR)	M_n (SEC)	$Đ$ (SEC)
DMAEMA	BMA	60,000	50%	54%	46,400	1.61
DMAEMA	BMA	60,000	60%	67%	48,700	1.44
DMAEMA	BMA	60,000	70%	67%	47,900	1.65
DMAEMA	BMA	60,000	80%	82%	46,900	1.73
DMAEMA	N/A	60,000	100%	100%	45,400	1.36

20.60 kg/mol poly(DMAEMA-*s*-TEGMA)

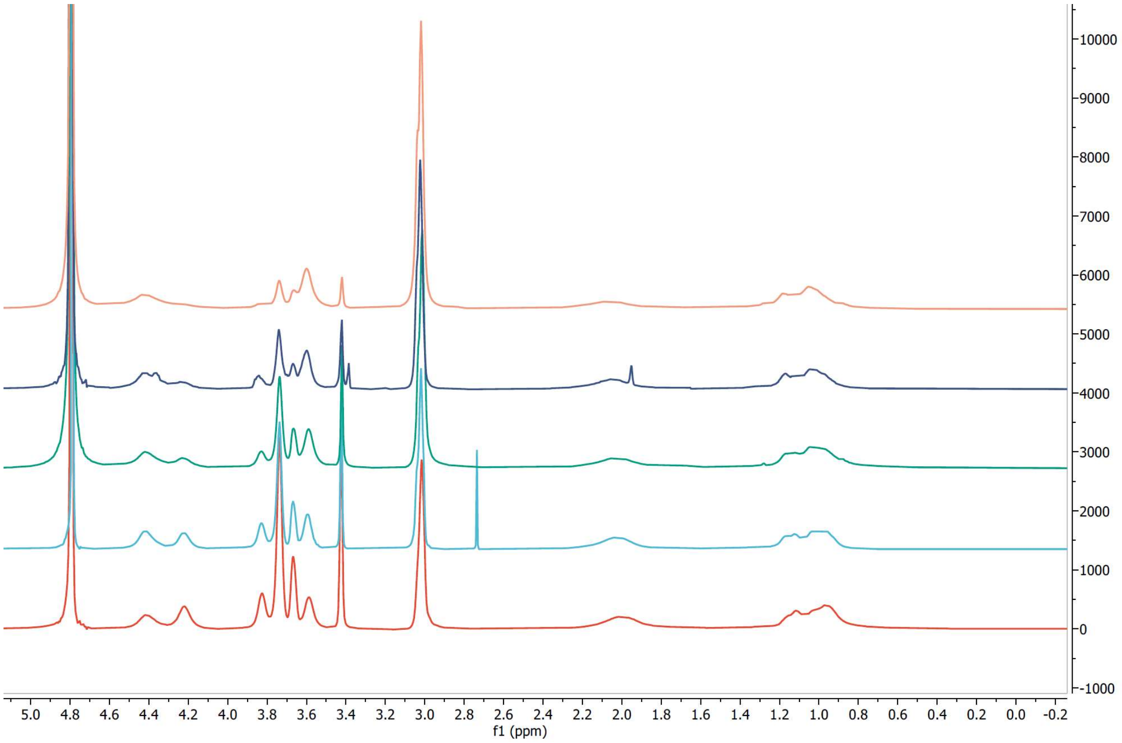

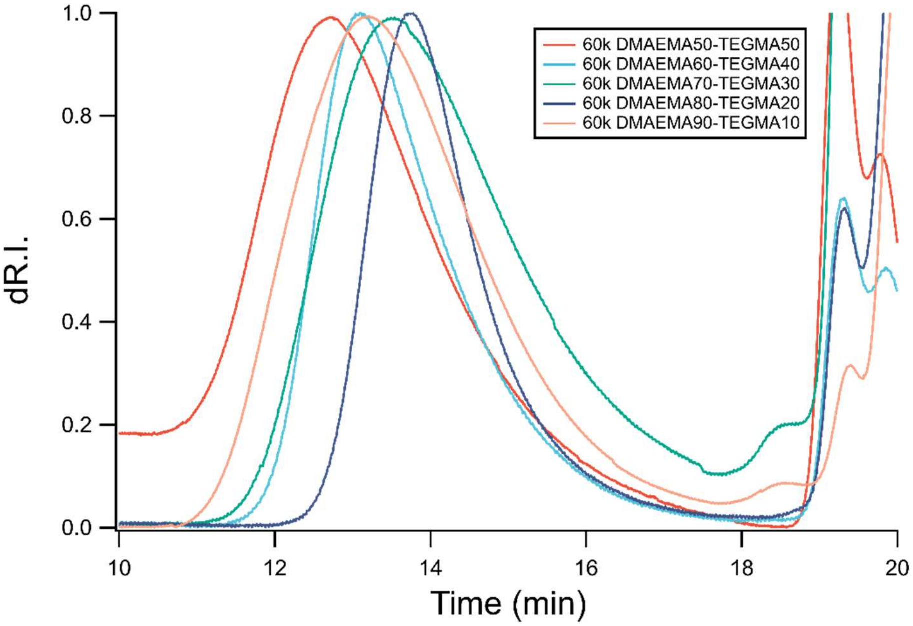

Mon1	Mon2	M_n (th)	%Mon1 (th)	%Mon1 (NMR)	M_n (SEC)	*Đ* (SEC)
DMAEMA	TEGMA	60,000	50%	48%	78,800	1.58
DMAEMA	TEGMA	60,000	60%	59%	47,200	1.57
DMAEMA	TEGMA	60,000	70%	67%	48,300	1.88
DMAEMA	TEGMA	60,000	80%	79%	50,800	1.24
DMAEMA	TEGMA	60,000	90%	88%	48,200	1.85

21. DMAEMA + 5 mol.% AEMA co-polymers (See **Figure S15** for accompanying data)

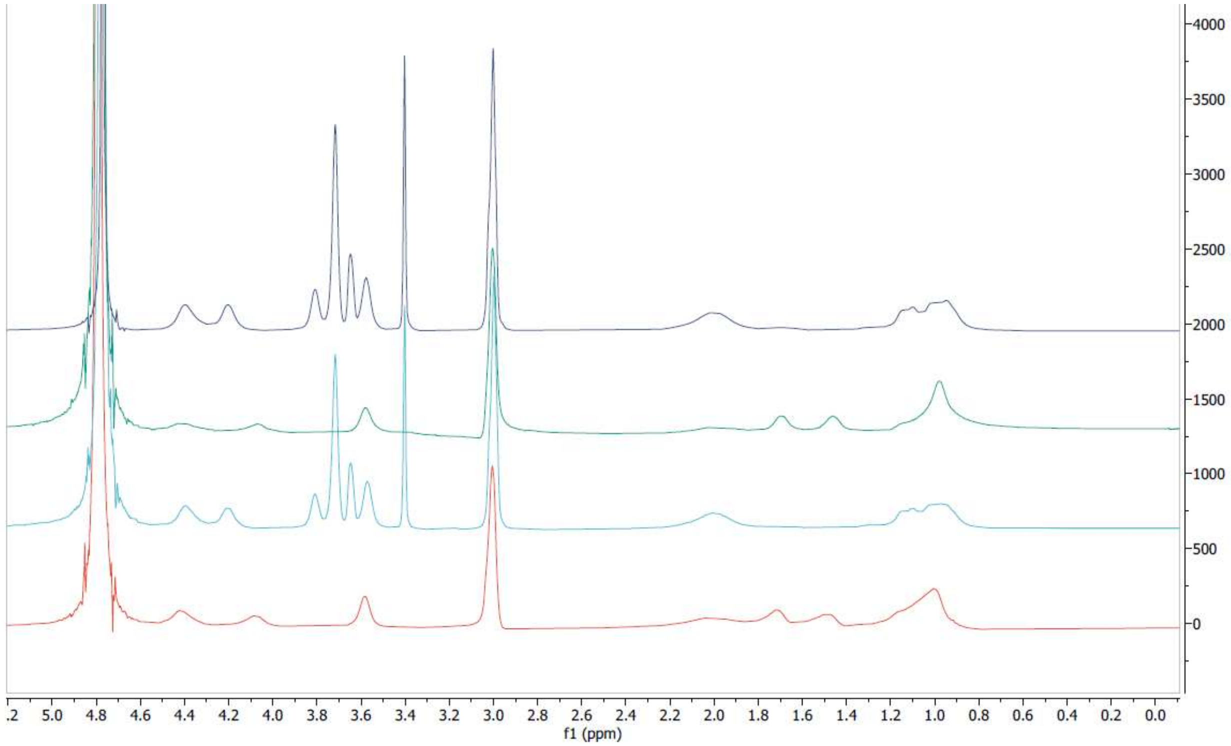

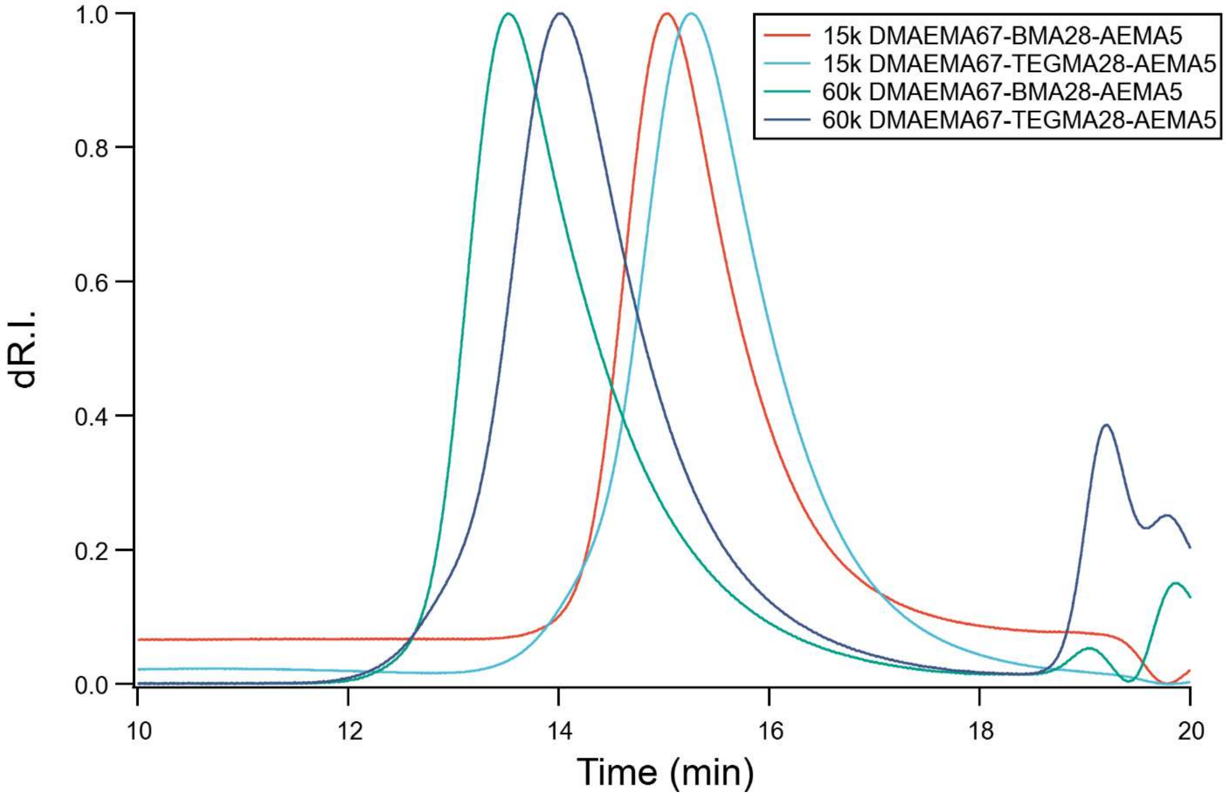

Descriptions of Supplementary Videos.

Supplementary Video 1: Time-lapse Video of Lysosensor Green Assay in WT THP-1 Cells. THP-1 cells were stained with LysoSensor Green DND-189, Propidium Iodide, and Hoechst 33342 and then treated with 100 µg/mL of the indicated polymers. Cells were imaged every 2 min for 1 h using confocal microscopy (40x oil lens) to evaluate lysosomal pH and cellular morphology.

Supplementary Video 2: Time-lapse Video of Lysosensor Green Assay in NLRP3-KO THP-1 Cells. NLRP3-KO THP-1 cells were stained with LysoSensor Green DND-189, Propidium Iodide, and Hoechst 33342 and then treated with 100 µg/mL of the indicated polymers. Cells were imaged every 2 min for 1 h using confocal microscopy (40x oil lens) to evaluate lysosomal pH and cellular morphology.

Supplementary Video 3: Time-lapse Video of Lysosensor Green Assay in Other Cell Lines. Murine bone marrow derived dendritic cells (BMDCs), HeLa cells, and A549 cells were stained with LysoSensor Green DND-189, Propidium Iodide, and Hoechst 33342 and then treated with 100 µg/mL of the indicated polymers. Cells were imaged every 2 min for 1 h using confocal microscopy (40x oil lens) to evaluate lysosomal pH and cellular morphology.

Supplementary Video 4: Time-lapse Video of THP-1 ASC-GFP Speck Formation. THP-1 ASC-GFP cells were stained with LysoView 633, Propidium Iodide (PI), and Hoechst 33342 and then treated with 100 µg/mL of the indicated polymers. Cells were then imaged every 2 min for 1 h using confocal microscopy (40x oil lens) to evaluate speck formation and cell death. Speck containing cells and PI-stained cells in each frame were counted by a single blind observer.

Supplementary Video 5: Time-lapse Video of THP-1 ASC-GFP Speck Formation with Cytochalasin D. THP-1 ASC-GFP cells were pre-treated with Cytochalasin D (2.5 μg/mL), stained with LysoView 633, Propidium Iodide (PI), and Hoechst 33342, and treated with 100 μg/mL of the indicated polymers. Cells were then imaged every 2 min for 1 h using confocal microscopy (40x oil lens) to evaluate speck formation and death. Speck containing cells and PI-stained cells in each frame were counted by a single blind observer.

Supplemental References

S1. Manna, S.; Howitz, W. J.; Oldenhuis, N. J.; Eldredge, A. C.; Shen, J.; Nihesh, F. N.; Lodoen, M. B.; Guan, Z.; Esser-Kahn, A. P., Immunomodulation of the NLRP3 Inflammasome through Structure-Based Activator Design and Functional Regulation via Lysosomal Rupture. *ACS Cent Sci* **2018**, *4* (8), 982-995.

S2. Fu, L.; Liu, L.; Ruan, Z.; Zhang, H.; Yan, L., Folic acid targeted pH-responsive amphiphilic polymer nanoparticles conjugated with near infrared fluorescence probe for imaging-guided drug delivery. *RSC Adv* **2016**, *6* (46), 40312-40322.

S3. Chen, A.; Wu, D.; Johnson, C. S., Determination of Molecular Weight Distributions for Polymers by Diffusion-Ordered NMR. *J Am Chem Soc* **1995**, *117* (30), 7965-7970.
